# Genome-wide survey of heat shock factors and heat shock protein 70s and their regulatory network under abiotic stresses in *Brachypodium distachyon*

**DOI:** 10.1371/journal.pone.0180352

**Published:** 2017-07-06

**Authors:** Feng Wen, Xiaozhu Wu, Tongjian Li, Mingliang Jia, Xinshen Liu, Peng Li, Xiaojian Zhou, Xinxin Ji, Xiaomin Yue

**Affiliations:** 1 School of Pharmacy and Life Science, Jiujiang University, Jiujiang, China; 2 Shanghai Chenshan Plant Science Research Center, Shanghai Institutes for Biological Sciences, Chinese Academy of Sciences (CAS). Shanghai Chenshan Botanic Garden, Songjiang, Shanghai, China; National Taiwan University, TAIWAN

## Abstract

The heat shock protein 70s (Hsp70s) and heat shock factors (Hsfs) play key roles in protecting plant cells or tissues from various abiotic stresses. *Brachypodium distachyon*, recently developed an excellent model organism for functional genomics research, is related to the major cereal grain species. Although *B*. *distachyon* genome has been fully sequenced, the information of *Hsf* and *Hsp70* genes and especially the regulatory network between Hsfs and Hsp70s remains incomplete. Here, a total of 24 BdHsfs and 29 BdHsp70s were identified in the genome by bioinformatics analysis and the regulatory network between Hsfs and Hsp70s were performed in this study. Based on highly conserved domain and motif analysis, BdHsfs were grouped into three classes, and BdHsp70s divided into six groups, respectively. Most of Hsf proteins contain five conserved domains: DBD, HR-A/B region, NLS and NES motifs and AHA domain, while Hsp70 proteins have three conserved domains: N-terminal nucleotide binding domain, peptide binding domain and a variable C-terminal lid region. Expression data revealed a large number of *BdHsfs* and *BdHsp70s* were induced by HS challenge, and a previous heat acclimation could induce the acquired thermotolerance to help seedling suffer the severe HS challenge, suggesting that the BdHsfs and BdHsp70s played a role in alleviating the damage by HS. The comparison revealed that, most *BdHsfs* and *BdHsp70s* genes responded to multiple abiotic stresses in an overlapping relationship, while some of them were stress specific response genes. Moreover, co-expression relationships and predicted protein-protein interaction network implied that class A and B Hsfs played as activator and repressors, respectively, suggesting that BdHsp70s might be regulated by both the activation and the repression mechanisms under stress condition. Our genomics analysis of BdHsfs and BdHsp70s provides important evolutionary and functional characterization for further investigation of the accurate regulatory mechanisms among Hsfs and Hsp70s in herbaceous plants.

## Introduction

Abiotic stresses, such as heat, cold, drought, and salinity, are the most harmful factors concerning the growth and productivity of crops, which represent seriously threat to agriculture and cause the huge loss of crop yield worldwide by more than 50% annually [1]. For instance, temperature is a major environmental factor that affects plant growth and productivity. If plant expose to high temperature beyond a threshold level for a period of time, it will cause significant adverse impact on almost all aspects of plant development and growth, including leaf damage, accelerated leaf senescence, ROS burst, and reduced photosynthesis capacity, and may drastically reduce plant biomass production and economic yield [2,3]. Since plants are especially dependent on environmental factors because of a sessile lifestyle, they have to evolve a spectrum of molecular programs to help them adapt to changing environmental conditions. In plants, the heat stress (HS) response is highly conserved and refers to multiple pathways and regulatory networks [4]. The plant cells respond to HS by inducing the transcription of genes encoding heat shock proteins (Hsp), which are involved in preventing or repairing the damage caused by elevated temperature and thus confer increased thermotolerance [5]. For instance, *Hsp70* gene family is a type of Hsps, plays key roles in protecting plant cells or tissues from heat stress as well as other environmental stresses, by function in degradation of misfolded and truncated proteins as molecular chaperones [6]. One of the regulatory networks that control the expression of Hsps is the heat shock factors (Hsfs) network. Hsfs are transcription factors (TFs), which can regulate the expression of Hsps by recognizing heat shock elements (HSEs) within the promoters of Hsps.

It is well-known that Hsps are ubiquitous proteins found in plant and animal cells, which were described in involve in heat shock [7]. Now, Hsps are known to be induced by a wide variety of stresses, including cold, drought, salt, UV-light, wound, and biotic stresses [8,9]. In plants and animals, Hsps are mainly located in the cytoplasm, could be classified into six major families based on their molecular masses, such as Hsp100, Hsp90, Hsp70, Hsp60, Hsp40 and small heat shock proteins (sHsps) [8,10]. Hsp70 family has long been recognized as one of the most conserved protein families among these six major Hsp families, and which is present constitutively and up-regulated in response to series of stressors like heat, cold, drought, salt and oxidation [11–13]. Hsp70s are ATP-dependent chaperones containing a ~44kD conserved N-terminal nucleotide binding domain (acting as a ATPase domain; NBD), a ~18kD substrate binding domain (SBD), and a ~10kD variable C-terminal lid region that covers the SBD [14]. In generally, Hsp70s has four major cognates, such as cytosolic Hsc70 (HspA8), endoplasmic reticulum Bip (HspA5), mitochondrial mHsp70 (HspA9) and related Hsp110s (HspHs), which have high sequence and structural homology to Hsp70 and are therefore included in Hsp70 superfamily [15,16]. The diverse biological functions of Hsp70s have been investigated in several plant systems. The most important biological function of Hsp70s is linked to acquired thermotolerance under HS, and function as a negative feedback regulator of HSF activity [17–20]. For example, as molecular chaperones, Hsp70s bind and release unfolded/non-native proteins, thereby helping polypeptides undergo productive folding, and then play a primary autoregulatory role by negatively regulating Hsf1 transcriptional activity during HS [21]. Hsp70 proteins in plants are also involved in additional specific functions. For example, knockout mutants of cpHsc70s showed defective phenotypes, such as variegated cotyledons and malformed leaves in plant development and the thermotolerance of germinating seeds [22,23]. A cytosolic/nuclear molecular chaperone (Hsc70-1) was the major stable SGT1 interactors, and the SGT1–Hsc70 association is important for the regulation of plant responses to biotic and abiotic stresses [24]. Elevated levels of BiP conferred tobacco seedling tolerance to tunicamycin during germination and tolerance to water deficit during plant growth, while it acts an important role in disrupting the fusion of polar nuclei in the proliferation of endosperm nuclei [25,26]. Also, Hsp70s show various functions in plant immunity, for instance, CaHsp70a interacts with the type III effector AvrBsT and is required for cell death and immunity in plants [27]. It was reported that host Hsp70 was necessary for rice stripe virus infection and probably played a role in viral replication by interacting with viral RdRp, and also was required for pathogens-induced cell death in tobacco, which was at least partially similar to HR response [28,29].

A multitude of *Hsf* family genes is extensively distributed among different eukaryotic organisms, role in regulating the expression of Hsps, which contribute to the increased tolerance against HS [30]. Similar to many other TFs, the Hsf family contains highly conserved domains, including DNA binding domain (DBD, also known as the HSF domain), the hydrophobic oligomerization domain and the nuclear localization signal domain (NLS) [31]. Also, some Hsfs have a nuclear export signal (NES) and the C-terminal activation domains (CTAD), which are characterized by short peptide motifs (AHA motifs), playing crucial activated function in many cases [32]. Members of this family are divided into 3 classes, A, B, and C, based on the peculiarities of their oligomerization domain (HR-A/B regions) [33]. Hsfs of class A and C contain insertions of 21 (class A) and seven (class C) amino acid residues between the HR-A and HR-B region, respectively, while those of class B are comparatively compact without an insertion [30,31,34]. Over the last two decades, numerous publications report that Hsfs had been integrated into the complex stress signaling and response networks of plants. Based on the previous studies, HsfA1 subfamily is defined as a master regulator of HS responses, which is responsible for triggering the HS response and later on, by interaction with Hsfs A2 and B1 in a functional triad [35–37]. For instance, tomato HsfA1a appears to have unique role as master regulator of thermotolerance, and cannot be replaced by any other Hsfs [35,38]. HsfA2 shows a similar structure and function to HsfA1 [39], but it is mainly retained in the cytoplasm unless it is co-expressed with HsfA1 in tomato, because the efficient nuclear transport of HsfA2 evidently requires interaction with HsfA1 [35]. Interestingly, all class B Hsfs, except HsfB5, contain a tetrapeptide -LFGV- in the C-terminal domain, which is speculated to function as a repressor motif by interaction with a hitherto unknown co-repressor [40,41]. For example, HsfB1/B2b may interact with class A-Hsf in regulating the shut-off of the heat shock response [41]. Also, large numbers of work show plants frequently suffer from various abiotic stresses simultaneously, Hsfs play an important role in HS, as well as in responses to other abiotic stresses such as cold, salt, and drought. The Arabidopsis HsfA1 mediated the induction of *Hsp* genes under salt, osmotic and oxidative stresses and conferred tolerance during plant growth and development [42]. Bechtold et al demonstrated that over-expression AtHsfA1b in Arabidopsis seedling showed increased water productivity and drought tolerance [43].

*Brachypodium distachyon*, commonly called purple false brome, recently developed an attractive model organism for functional genomics research due to a number of advantageous features, including its small genome size, simple growth conditions, short lifecycle, and genetic tractability [44,45]. The whole-genome sequence of *B*. *distachyon* was completed and it helps scientists better understand grass genome evolution [46]. Based on the genome sequencing data, the genus *B*. *distachyon* is related to the major cereal grain species such as wheat, barley, *Sorghum bicolor* and *Triticum aestivum* [46]. The available of its whole genome sequence makes it a promising model for functional genomic studies of crops and herbaceous, and mechanisms of gene controlled physiological processes in Poaceae. Here, we further identify *B*. *distachyon Hsf* and *Hsp70* genes and analyze their expression profiles under different abiotic stresses. Totally, 24 *Hsfs* and 29 *Hsp70s* were identified from *B*. *distachyon* Bd21 genome, and the genetic characterizations of *B*. *distachyon Hsfs* and *Hsp70s*, including phytogenetic analysis, chromosomal localization and gene duplication, have been systematically investigated. Subsequently, a heat-induced expression profile was carried out to investigate the function of heat-induced BdHsfs in response to HS. Further, an expression heatmap of *BdHsfs* and *BdHsp70s* in response to other abiotic stresses were also exhibited. The whole-genome expression profile analysis showed that the BdHsfs and BdHsp70s played a role in alleviating the damage by HS, and these *B*. *distachyon* Hsfs and Hsp70s responded to different stresses in an overlapping relationship. Our analysis also indicated that some *Hsf* and *Hsp70* genes exhibited specific expression patterns in response to distinct stresses. And predicted regulatory network between *B*. *distachyon Hsfs* and *Hsp70s* was also discussed.

## Materials and methods

### Sequence retrieval and genetic characterizations analysis

Published Arabidopsis and rice Hsf and Hsp70 sequences were used as queries in BLASTP searches against 32255 sequences of the protein database of *B*. *distachyon* from MIPS (http://mips.helmholtz-muenchen.de/plant/brachypodium/), which resulted in 101 hits as subject sequences [46]. A self BLAST of these sequences followed by manual editing to remove the redundancy finally resulted in identification of 24 *Hsf* and 29 *Hsp70* genes (S1 Table). To verify the reliability of our results, all putative non-redundant sequences were assessed with UniProt and SMART (http://smart.embl-heidelberg.de/) analysis, respectively. WoLF PSORT (http://psort.hgc.jp/) was used to predict their protein subcellular localizations [47]. The theoretical pI (isoelectric point) and Mw (molecular weight) were estimated using the Compute pI/Mw tool from ExPASy (http://web.expasy.org/compute_pi). The phylogenetic trees were constructed using the neighbor-joining method in the MEGA version 5 software, with bootstrap values from 1,000 replicates indicated at each node with the following parameters: p-distance and pairwise deletion [48].

### Chromosomal locations and conserved motif analysis

The chromosomal locations of the Hsf and Hsp70 genes were determined using the *B*. *distachyon* genome browser (https://phytozome.jgi.doe.gov). The gene duplications within the *Hsf* and *Hsp70* gene family in *B*. *distachyon* genomes were based on the information from the Plant Genome Duplication Database (http://chibba.agtec.uga.edu/duplication/index/locus). SMART, PredictNLS, and NetNES1.1 were used to check DBD domains and coiled-coil structures and NLS and NES domains of BdHsfs, and protein domains of BdHsp70s were predicted using InterProScan and Conserved Domain Database (CDD) against protein databases (S2 Table) [49–52]. The MEME web server was used to analyze motifs in *B*. *distachyon* Hsp70 and Hsf proteins (S1 Fig) [53]. The parameters were set as follows: maximum numbers of different motifs, 18 and 15 for Hsf and Hsp70 proteins, respectively; minimum motif width, 6; maximum motif width, 50. The results were then downloaded and submitted to Expasy to generate the pictures [54].

### Publicly available microarray data analyses

The *B*. *distachyon* expression data under abiotic stresses were downloaded from the Plant Expression Database (www.plexdb.org), which has been reported by using Affymetrix Brachypodium Genome Array (BradiAR1b520742) [55]. Venn diagram showed the overlap of the numbers of up-regulated genes in response to different abiotic stresses, which has been reported previously [55].

### Analysis of expression data

The 2-week old seedlings (Bd21) were used for tissue-specific expression analysis and abiotic stresses treatment according with the previous work with some modifies [56]. For tissue-specific expression analysis, different tissues including roots, stems and leaves were collected and used for RNA extraction. For HS, 2-week-old seedlings were treated with normal HS (group I, 37°C 2 h), normal HS with RP (group II, 37°C 2 h, following 2 h 22°C recovery), severe HS with RP (group III, 45°C 2h, following 2 h 22°C recovery) and severe HS with a CT (group IV, seedling was first pretreatment 2 h at 37°C, return to 22°C for 2 h, heated to 45°C for 2 h, and then allowed to 22°C for 2 h) respectively (S2 Fig). For abiotic stress treatment, 2-week-old seedlings were treated in MS liquid medium containing 20% PEG, 200 mM NaCl and 10 mM H_2_O_2_ for 2 h, respectively. Cold and heat treatments were achieved by placing 2-week-old seedlings in MS liquid medium at 4°C or 37°C for 2 h, respectively. Two-week-old seedlings in MS liquid medium growth at 22°C were set as the control group. The samples of each treatment were collected three replications. The *Hsf* and *Hsp70* genes array constituted of 53 primer-sets representing all members of the *B*. *distachyon* Hsf and Hsp70 gene families. The primer-sets were listed in S4 Table. The expression of *Hsf* and *Hsp70* genes was assessed upon the qPCR result analysis. The expression profiles of tissue-specific analysis and stress treatment analysis were calculated from the –Δ*C*T value [–Δ*C*T = –(*C*Tgene–*C*Tactin)] and –ΔΔ*C*T value [–ΔΔ*C*T = (*C*Tcontrol.gene–*C*Tcontrol.actin)–(*C*Ttreat.gene–*C*Ttreat.actin)], respectively, obtained by PermutMatrixEN version 1.9.3 software, and shown by a green-red gradient. The data were statistically analyzed using OriginPro 8.0 software. The up-regulated genes were defined as a fold-change greater than 2 with *p-value* < 0.05 and a fold change of 0.5 or less was used to define down-regulated genes when the *p-value* < 0.05 (S3 Table). Expression correlation of *Hsfs* and *Hsp70s* (*Hsp110s* subgroup was separately analyzed) between any two of heat, cold, salt, drought and oxidative stress has been analyzed by using OriginPro 8.0 software. Pearson correlation coefficient (R) represented the degree of co-regulation between two stresses.

### Regulatory network construction

The expression data of *Hsfs* and *Hsp70s* were clustered together to form an integrated expression profile by Cluster 3.0 software and visualized by using TreeView software. The *Hsf* and *Hsp70* genes, whose correlation coefficients of expression profiles were greater than 0.8, were clustered together as a set of co-expression regulatory *Hsf* and *Hsp70* genes under different kinds of treatment conditions, including heat, cold, drought, salt and oxidative stresses. The network picture which represented the co-expression regulatory network was created using Cytoscape [57]. The predicted protein-protein interaction (PPI) network was generated by STRING V10.0 software online (http://string.embl.de/newstring_cgi/).

## Results and discussion

### Overview of genome-wide response to abiotic stresses in *B*. *distachyon*

**Fig 1:**
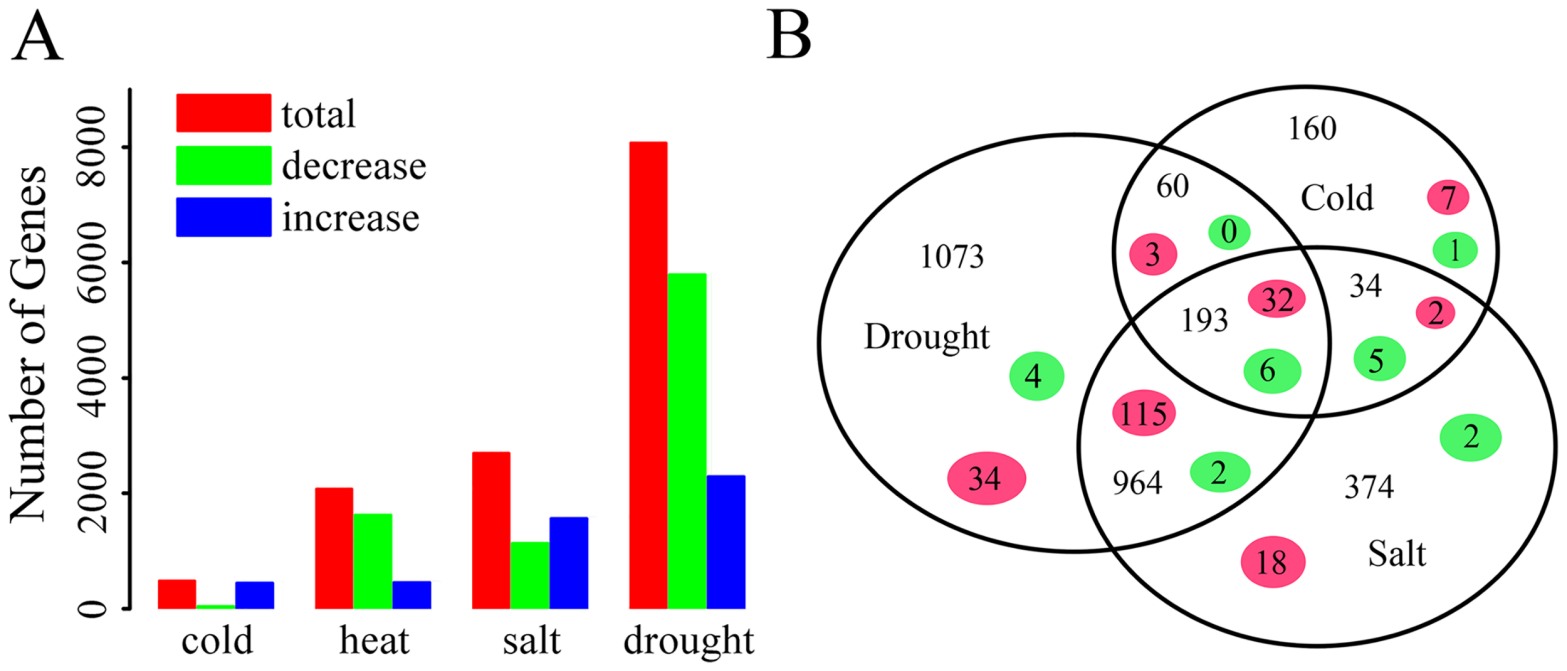
A summary of differentially expressed genes of *B*. *distachyon* seedlings under heat, cold, drought and salt stresses. (A) The number of up-/down-regulated genes under four abiotic stresses. Vertical axis indicated the differentially expressed gene numbers. Horizontal axis indicates four stresses. Red, green, and blue bars represented the total, decreased, and increased probe sets, respectively. (B) Venn diagram of differentially expressed genes under four abiotic stresses. Three circles indicate the differentially expressed genes under cold, drought and salt stresses. Numbers in red circles indicated gene numbers co-regulated by heat stress and other stresses, while numbers in green circles indicated the number of *BdHsf* and *BdHsp70* genes co-regulated by heat stress and other stresses in our qPCR experiment.

Although the genome-wide transcript-level gene expression changes in response to abiotic stresses treatment in *B*. *distachyon* has been reported by using Affymetrix Brachypodium Genome Array (BradiAR1b520742) [55], the characterization of the response network between heat and other abiotic stresses remained unclear. In this study, we comprehensively compared *B*. *distachyon* gene expression patterns under HS with that under other stresses. The *B*. *distachyon* expression data under these abiotic stresses were downloaded from the Plant Expression Database (www.plexdb.org) [55]. Gene numbers that were up- or down-regulated by each stress were shown in Fig 1A. As reported in previous paper, the number of responsive genes under heat, cold, drought and salt treatments were 2079, 487, 8080 and 2702, respectively. Among them, the number of genes that were up-regulated by heat, cold, drought and salt was 458, 447, 2290 and 1565, respectively, while the download-regulated genes were 1621, 40, 5790 and 1137, respectively [55]. The number of overlapped genes which were response to two or more stresses was analyzed. Venn diagram showed that a large number of genes were expressed under two or more stresses (Fig 1B). The number of overlapping up-regulated genes between HS and each of cold, drought and salt stresses were 44, 184 and 167, respectively. These results showed that the numbers of overlapping genes between HS and drought/salt were much more than cold, suggesting that plant may share some of responding mechanism in respond to heat, drought and salt stress, rather than in respond to cold. Although there were 211 genes responding to both heat and each of other stress, expression data showed genome-wide expression patterns under heat stress were different from cold, drought and salt, which was consistent with previous studies in Arabidopsis and rice [1,58]. Since plant was simultaneously faced with wide variety of stresses during their growth period, multiple stresses response mechanism was urgent to study. Therefore it would be helpful to identify co-regulators from the 211 genes that responded to multiple stresses.

### Identification of *B*. *distachyon Hsp70s* and *Hsfs* and their distribution on chromosomes

*B*. *distachyon Hsp70s* and *Hsfs* were identified by similarity searching against the *B*. *distachyon* genome sequence data using *Hsf* and *Hsp70* genes in Arabidopsis and rice followed by manual check [1,59]. This analysis has revealed that 24 *Hsf* and 29 *Hsp70* genes were identified from the *B*. *distachyon* genome (S1 Table). A protein subcellular localization prediction has been executed by WoLF PSORT online analysis, and analyses of biochemical properties (e.g. length, molecular weight and isoelectric point) of these proteins were also performed (S2 Table). The results revealed that most of the Hsfs were located in nuclear except for BdHsf-05, -06, and -14, which were presumptively present in chloroplast or cytoplasm. The Hsp70s were mostly located in cytoplasm, while Bips, cpHsp70s and mtHsp70s were present in ER, chloroplast and mitochondria, respectively (S2 Table).

**Fig 2:**
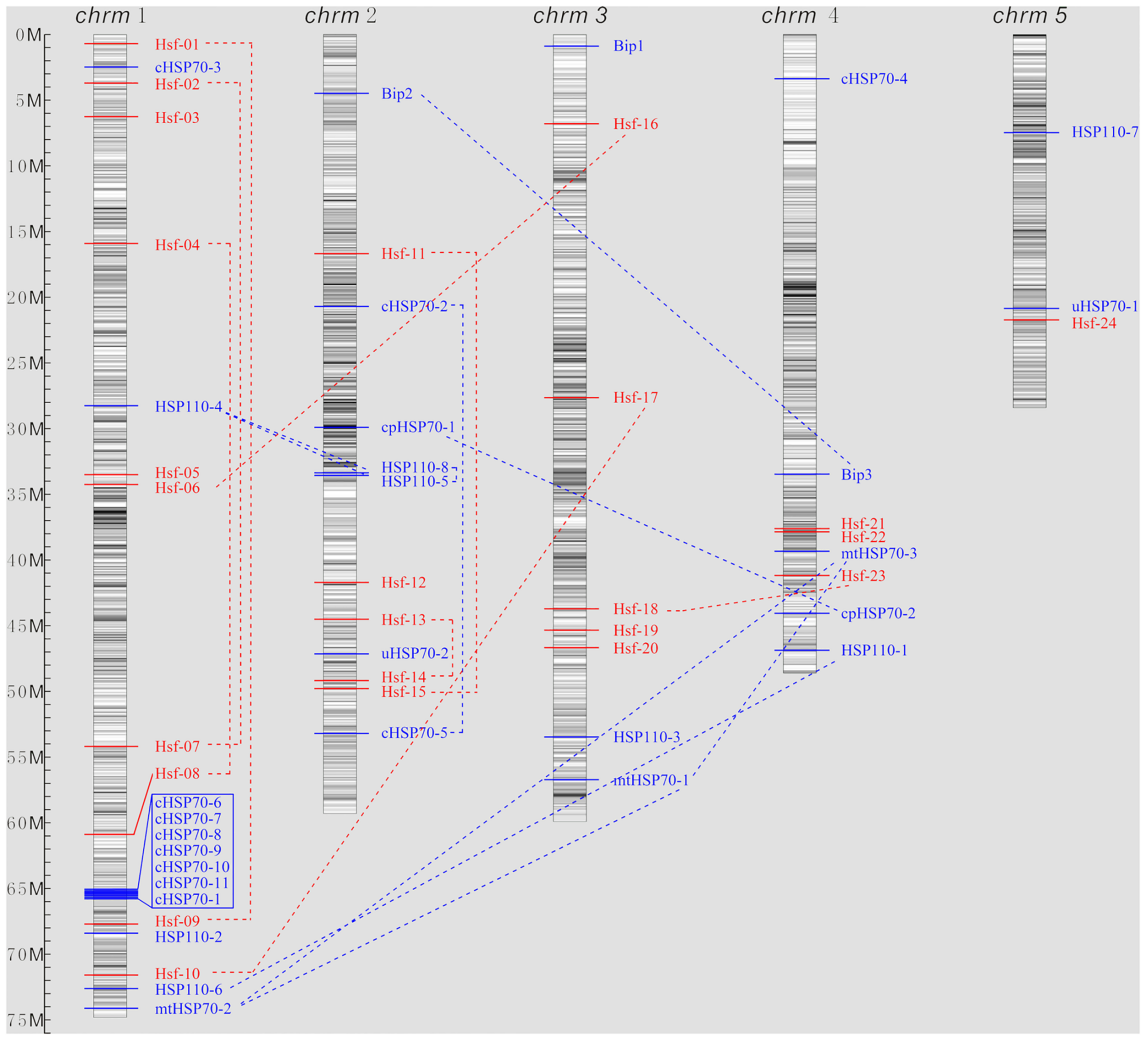
Chromosomal locations and gene duplication for *B*. *distachyon Hsf* and *Hsp70* genes. The chromosomal position of each *BdHsf* and *BdHsp70* gene was mapped according to the *B*. *distachyon* genome. The chromosome number is indicated at the top of each chromosome. The CpG island distribution maps shown in each chromosome depended on the CpG density in *B*. *distachyon* genome. The dotted line showed the gene duplication events among these *BdHsf* and *BdHsp70* genes. The scale bar was show on the left.

To further investigate the genomic distribution and gene duplication of these gene families, *Hsp70* and *Hsf* genes were plotted on chromosomes based on the information from the *B*. *distachyon* genomic database (https://phytozome.jgi.doe.gov). The physical locations of the *Hsp70* and *Hsf* genes on five chromosomes and the CpG island distribution map were depicted in Fig 2. It was found that 24 *Hsfs* and 29 *Hsp70s* were mainly mapped on chromosomes 1–4, whereas chromosome 5 only encoded 2 *Hsp70* and 1 *Hsf* gene. Genome-wide epigenetic studies showed that although CpG islands were thought to have a major role in control of gene expression by DNA methylation [60], more recent data suggested that the lower density region of CpG islands may be more important for distal regulation of gene expression [61–63]. It has been considered that high density CpG regions appear to regulate genome activity in house keeping and tissue specific genes [64]. Interestingly, our data showed that all members of *Hsf* and *Hsp70* genes were located at the low density region of CpG islands on the all of the 5 chromosomes, suggesting that the expression of these genes can be regulated by other genes or component. As shown in Fig 2, 17 pairs of duplication genes were identified, including 7 duplication events within the same chromosome and 10 segmental duplication events between chromosomes. Moreover, it was represented that plastid and mitochondrial *Hsp70s* are highly conserved, which was consistent with which in Arabidopsis [12]. As is known to all, many genes from different origins could be recombined into another organism or chromosome to form a new member of the large gene family, by base substitutions, deletions and insertions. These results revealed that members of *Hsp70* and *Hsf* family genes might be the result of genomic rearrangements and expansions during the process of evolution, particularly *cpHsp70s* and *mtHsp70s*. Interestingly, seven *Hsp70* copies were present in a single chromosomal locus at the end of the chromosome 1. The nucleotide sequence and spacing of the *Hsp70* copies were consistent with tandem duplication of the *Hsp70* genes in Drosophila, suggesting that tandem duplication played an important role in the expansion of *Hsp70s* [65].

### Genetic characterizations of *B*. *distachyon* Hsfs and Hsp70s and their tissue specific expression

**Fig 3:**
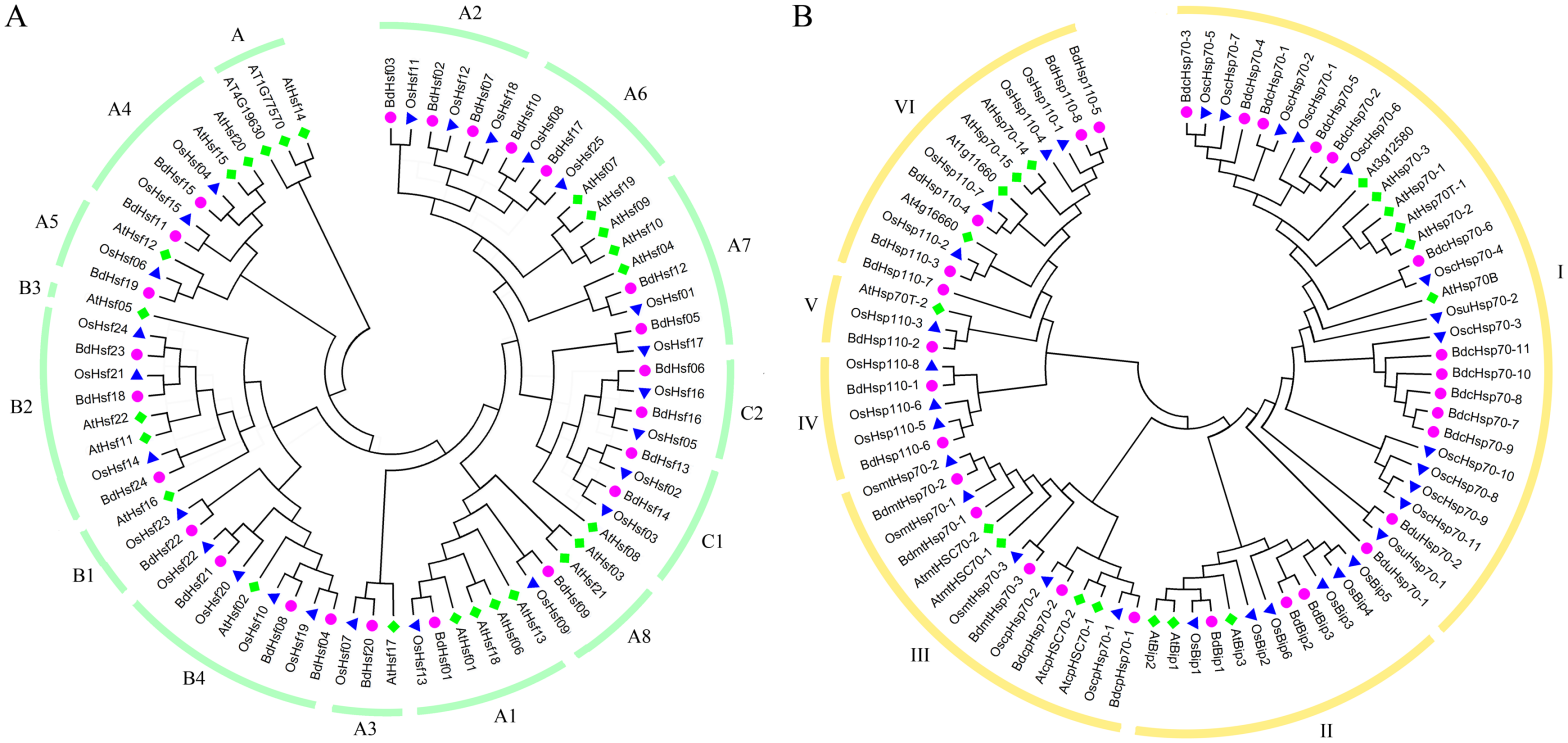
Neighbor-Joining phylogenetic trees of Hsf (A) and Hsp70 (B) proteins from Arabidopsis, rice and *B*. *distachyon*. The phylogenetic trees were constructed using the neighbor-joining method in the MEGA version 5 software, with bootstrap values from 1,000 replicates indicated at each node with the following parameters: p-distance and pairwise deletion.

To examine the evolutionary relationships of Hsfs and Hsp70s in *B*. *distachyon*, *A*. *thaliana* and *O*. *sativa*, phylogenetic trees were constructed from alignments of the full Hsfs and Hsp70s amino acid sequences using the Neighbor-Joining (NJ) method by MEGA5.0 (Fig 3). The gene model and amino acid sequences of Hsfs and Hsp70s in *B*. *distachyon*, *A*. *thaliana* and *O*. *sativa* were shown in S1 File. The phylogenetic analysis indicated that BdHsfs can be divided into three major subgroups corresponding with *A*. *thaliana* and *O*. *sativa*, which were consistent with the previous report [33]. Based on the phylogenetic tree, class HsfA had the maximum number of subclasses among the three classes, and included eight smaller clusters of which five (A1, A2, A6, A7, and A8) were closer to class HsfC than the other cluster of class HsfA (A3, A4 and A5). Unlike *A*. *thaliana*, class HsfB in *B*. *distachyon* included three smaller cluster (B1, B2 and B4), without HsfB3, which was consistent with those in *O*. *sativa*. Our phylogenetics analysis with 32 rice, 18 Arabidopsis and 29 *B*. *distachyon* Hsp70 proteins revealed six subfamilies (Fig 3) [66]. Cluster I has 13 *B*. *distachyon* Hsp70 members, and 5 of them form a cluster were defined as a *B*. *distachyon* divergent group, which were almost exactly the *Hsp70* members in the tandem duplication region at the end of the chromosome 1. Members in cluster II were BiP gene family, two of which were predicted to be localized in the ER lumen, while another one might be also present in cytoplasm. Cluster III consisted of five members with rice orthologs, two in the chloroplast and three in the mitochondria. Cluster IV, V and VI were belonged to Hsp110s, which were also included in Hsp70 superfamily. Compared with Arabidopsis, rice Hsfs and Hsp70s were closer to *B*. *distachyon* Hsf and Hsp70 proteins, which was coincident with the botanical classification.

**Fig 4:**
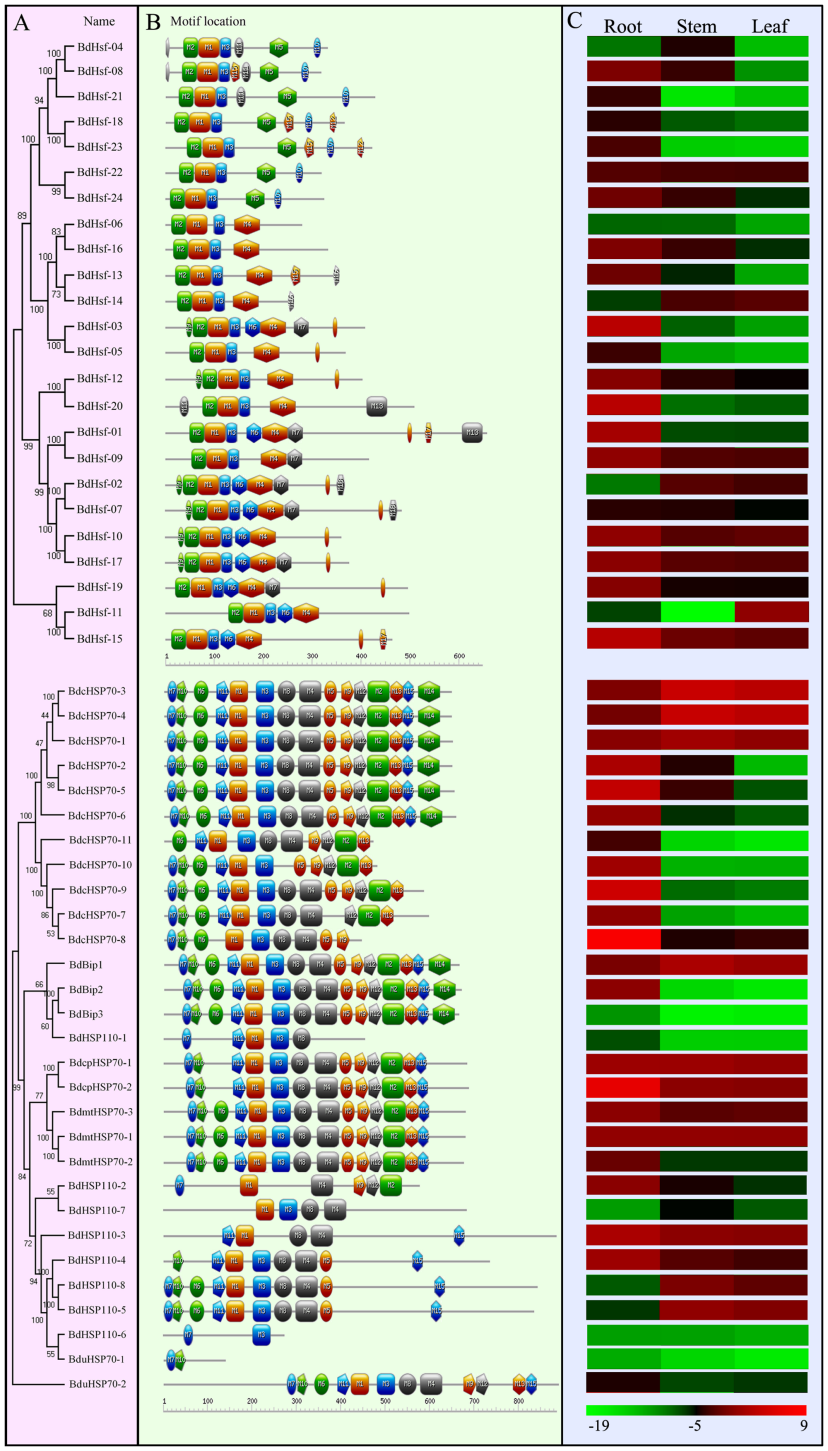
Phylogenetic relationships, protein structures and tissue specific expression heatmap of *Hsfs* and *Hsp70s* in *B*. *distachyon*. (A) The phylogenetic tree was constructed from the nucleotide sequences using the NJ program from MEGA 5, representing relationships among BdHsfs and BdHsp70s, respectively. The numbers beside the branches represent bootstrap support values (>50%) from 1000 replications. (B) Structure of Hsf and Hsp70 proteins were analyzed by MEME web server, and pictures were generated by Expasy web server. The details of sequence logo of motifs were shown in S1 Fig. (C) Quantitative RT-PCR analysis of the expression levels of *BdHsf* and *BdHsp70* genes in different tissues. The expression profile was shown by a green-red gradient using the PermutMatrix program. Results were normalized using *BdActin* (*Bradi2g24070*) gene expression as the internal control. Numbers under heatmap indicated the –Δ*C*T value [–Δ*C*T = –(*C*Tgene–*C*Tactin)].

Moreover, typical conserved domains of BdHsf and BdHsp70 families were further investigated. SMART, PredictNLS, and NetNES1.1 were used to check DBD domains and coiled-coil structures and NLS and NES domains of BdHsfs, and protein domains of BdHsp70s were predicted using InterProScan and Conserved Domain Database (CDD) against protein databases (S2 Table) [49–52]. The MEME web server was used to analyze motifs in *B*. *distachyon* Hsp70 and Hsf proteins (S1 Fig) [53]. The results were then downloaded and submitted to Expasy to generate the pictures (Fig 4) [54]. Phylogenetic trees constructed from alignments of *Hsp70* and *Hsf* nucleotide sequences were showed in the left of the protein structural drawing (Fig 4). Generally, the *Hsp70* and *Hsf* genes clustered together by phylogenetic analysis shared a similar protein structure, respectively (Fig 4). A typical Hsf protein in the plant kingdom contained five conserved domains: DBD, HR-A/B region, NLS and NES motifs and AHA domain. These domains enabled Hsf proteins to perform the functions associated with stress tolerance efficiently. All 24 BdHsf proteins contained a same combination, composed by motif 1, 2 and 3, which formed a DNA binding domain (DBD). Motif 4, which was considered as the region of HR-A/B, was present in class A and C Hsfs, instead of a compact HR-A/B region (i.e., motif 5) in class B Hsfs. Most Hsfs of class A contained a motif 6 followed by a motif 4, however, motif 6 was absent in class C Hsfs (Fig 4). As is well known, an Hsp70 protein had three conserved domains: N-terminal nucleotide binding domain (acting as an ATPase domain), peptide binding domain (also named substrate binding domain), and a variable C-terminal lid region. As expected, the protein structural schematic revealed that BdHsp70s was highly conserved. The amino acid sequences of BdHsp70s were very similar, particularly in the ATPase domain, and within the same subcellular groups (Fig 4B). Interesting, five of BdHsp70s (cHsp70-7, -8, -9, -10 and -11) were C-terminal deficiency compared with the other two BdHsp70s (cHsp70-1 and -6), which were also cluster in the tandem duplication region locus at the end of the chromosome 1. These result suggested that the terminal deficient Hsp70s might be originating from cHsp70-1 or cHsp70-6, and some nucleotide deletion events might occur during the gene family expansion. It is presumed that these members of *Hsp70* gene family might share a common ancestor as a result of a duplication event, which was denoted as being paralogs.

It has been reported previously that the different members of large gene families were diverse in sequence and function displaying various levels of expression among different tissues with separate regulation controls [67]. The function diversity of gene family members in different physiological process has been considered to be a result of differential expression in the tissues and/or at different developmental stages [68]. Thus, gene tissue specific expression analysis can provide important information for gene functions and gene regulations, particularly for members of large gene family. The expression levels of *B*. *distachyon Hsp70s* and *Hsfs* in different tissues (including root, stem and leaf) were detected by real-time PCR (Fig 4). The tissue-specific expression patterns of *B*. *distachyon Hsp70s* and *Hsfs* indicated that *BdHsfs* and *BdHsp70s* might be widely involved in the development of various organs and tissues, which was helpful to further understanding the functions of *BdHsf* and *BdHsp70* genes in *B*. *distachyon* developmental biology [69]. Generally, the results revealed that most of *Hsf* and *Hsp70s* genes were highly expressed in root, which was consistent with the expression data from JGI database. As shown in the Fig 4A, *Hsf01*, *-03*, *-15* and *-20* showed higher expression levels than other members in root. In Fig 4B, data showed that the expression levels of most *Hsp70* genes were extremely low in leafs, except *cHsp70-1*, *-3* and *-4*. Particularly, it has been found that several *BdHsp70* genes (e.g. *cHsp70-1*, *cHsp70-3*, *cHsp70-4*, *cpHsp70-2*, *Bip1* and *mtHsp70-1*) were expressed in all three tissues, suggesting that these *Hsp70* genes might be involved in some physiological processes in these three tissues. The expression pattern of *BdHsf* and *BdHsp70* genes suggested that *Hsf* and *Hsp70s* genes were involved in the growth and development of organs or tissues under specific conditions. Interestingly, many pairs of paralogs were clustered together with similar expression patterns, such as *Hsf18/Hsf23*, *Hsf10/Hsf17*, *cHsp70-2/cHsp70-5*, *cHsp70-3/cHsp70-4*, *cpHsp70-1/cpHsp70-2*, *Hsp110-5/Hsp110-8*, and so on, suggesting that these pairs of paralogs, which has more similarities in protein structure and shared similar expression patterns, might be functionally redundant (Fig 4B). Also, some pairs of paralogs showed distinct expression patterns (e.g. *Hsf11/Hsf15*, *Bip2/Bip3*, and *mtHsp70-1/mtHsp70-2*), suggesting a functionally diversity.

### Expression profile of *Hsp70* and *Hsf* genes upon multiple abiotic stresses

**Fig 5:**
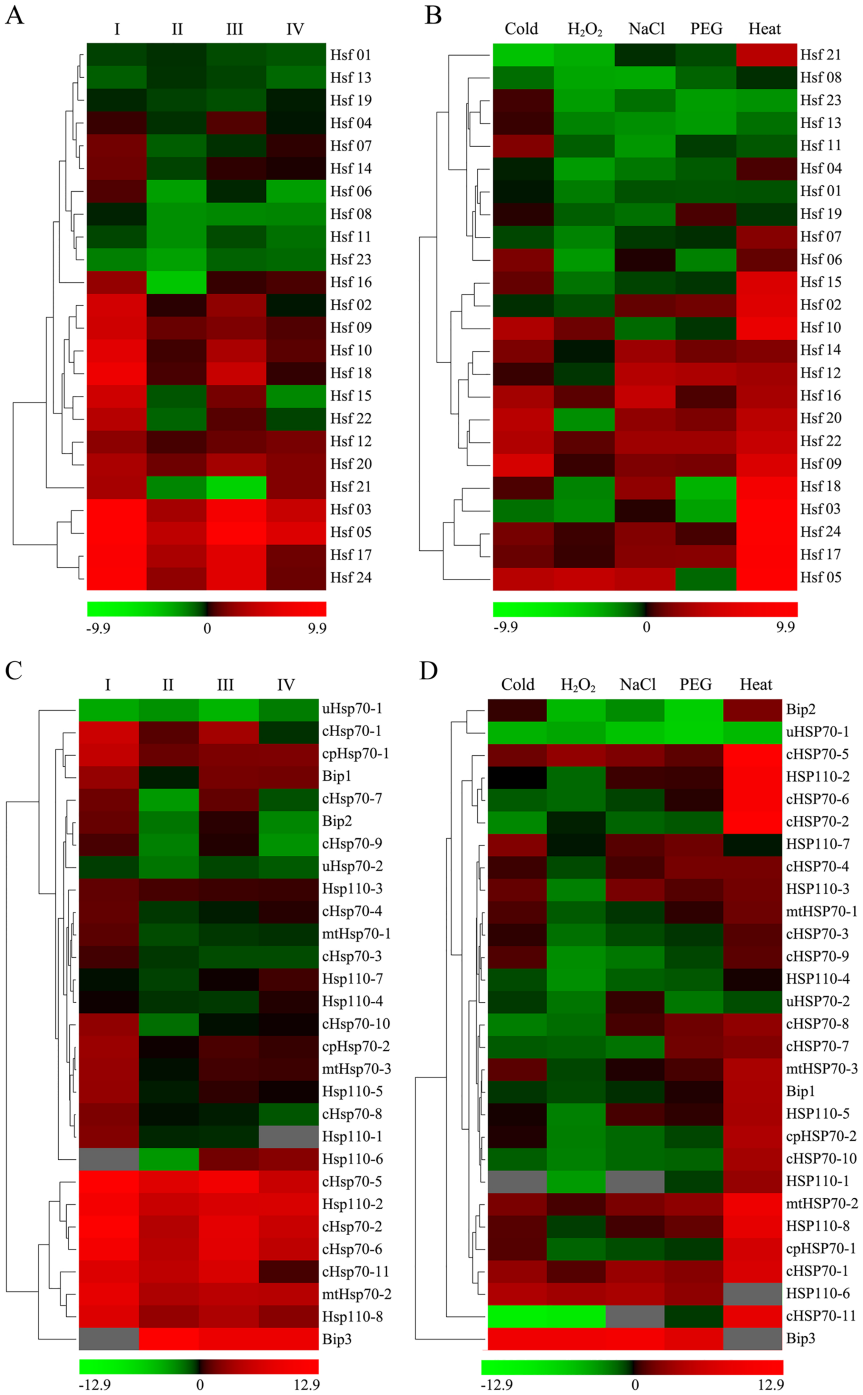
Expression profiles of *BdHsf* and *BdHsp70* genes. (A-B) Expression patterns of *Hsf* genes in *B*. *distachyon* in response to HS challenge (A) and multiple abiotic stresses (B). (C-D) Expression patterns of *Hsp70* genes in *B*. *distachyon* in response to HS challenge (C) and multiple abiotic stresses (D). The expression values of the *BdHsf* and *BdHsp70* genes were assessed upon the qPCR result analysis. The expression profile was shown by a green-red gradient using the PermutMatrix program. Numbers under heatmap indicated the –ΔΔ*C*T value [–ΔΔ*C*T = (*C*Tcontrol.gene–*C*Tcontrol.actin)–(*C*Ttreat.gene–*C*Ttreat.actin)].

It has been demonstrated that the most important function of *Hsf* and *Hsp70* genes was acting crucial roles in the control of plant response to multiple environmental stimuli and enhancing stress tolerance [4,14,32,42]. Firstly, to investigate the role of heat-inducible Hsfs and Hsp70s in thermotolerance, the expression profiles of *Hsf* and *Hsp70* genes under a series of HS challenge condition were examined using the qRT-PCR in our study (S2 Fig and S3 Table). As shown in Fig 5A, most of Hsf genes were rapidly and significantly up-regulated after normal HS challenge (37°C, 2 h), such as *Hsf02*, *-03*, *-05*, *-09*, *-10*, *-17*, *-18*, *-24*, and so on. However, after a 2-h recovery period (RP) from the 37°C normal HS challenge, the induced transcription levels of the Hsf genes were relative lower than those without RP, suggesting that the Hsf genes might be rapidly expressed in the initial phases responding to HS, and then regulated the expression of downstream gene to trigger the plant acquired resistance pathway. Moreover, the expression levels of the detected genes after the severe HS challenge with RP (45°C 2h, following 2 h 22°C recovery) were relatively high compared with that of normal HS challenge with RP. Even more interestingly, the expression levels of all heat inducible *Hsf* genes was significantly lower after severe HS challenge following a conditioning treatment (CT, 37°C, 2 h) than that of without a CT. This result indicated that a previous heat acclimation could up-regulate the *Hsf* genes expression, and then induced the plant to acquire thermotolerance at seedling level, which was reported previously [5,70]. The acquired thermotolerance induced by CT could protect the *B*. *distachyon* seedling against the severe HS challenge, which might result in lower expression level of *Hsf* genes. On the other hand, the accumulation of Hsp70s regulated by Hsfs, which induced by CT, might also be a reason of decreased *Hsf* gene expression, since Hsp70s might function as a negative feedback regulator of Hsfs [20]. *BdHsp70* genes showed similar expression profile to *BdHsf* genes under HS challenge condition (Fig 5C). Similar, the expression of *Hsp70s* could be also down-regulated to their initial levels when plants recovered 2 h from normal HS challenge [71]. A large number of *BdHsp70* genes were induced by HS challenge, and the expression levels of these genes was much low in the severe HS treated seedling which has been performed a previous heat acclimation than those without it (Fig 5C). The similar patterns of *Hsfs* and *Hsps* gene expression in response to HS challenge indicated that the response of these genes might have same motifs in their promoters [1,72].

Further, the expression profiles of the *BdHsf* and *BdHsp70* family genes under different abiotic stress conditions were also performed using the qRT-PCR (S3 Table). Including HS, a total of five stress types, i.e. heat, cold, NaCl, PEG, and H_2_O_2_, were tested in this study. Firstly, the expression levels of five genes, including *BdWRKY36*, *BdCBF1*, *BdCBF2*, *BdP5CS1* and *BdAPX1*, were determined as marker genes to evaluate the abiotic stresses treatment (S3 Fig). Results showed that *BdCBF1* was up-regulated rapidly after cold treatment, while *BdCBF2* showed an obviously increased expression under salt and drought stresses, which was consistent with previous studies on *BdCBF* gene functional characterization [73]. *BdWRKY36* expression was up-regulated by cold and drought stresses, similar results were reported in previous paper [74]. APXs were the key enzymes in a major hydrogen peroxide-detoxifying system in plant, which was also called ascorbate-glutathione cycle [75]. In our study, *BdAPX1* not only showed a high up-regulation in response to oxidative stress, but also in the response to other three stresses. Heatmap representation of expression profiles of these *BdHsf* and *BdHsp70* family genes under multiple abiotic stresses were shown in Fig 5B and 5D. The data revealed that 70% of *BdHsf* and *BdHsp70* genes were up-regulated under heat stress conditions. Many *BdHsf* and *BdHsp70* genes were up-regulated under more than one stress conditions, suggesting that BdHsfs and BdHsp70s not only played a critical role in response to heat stress, but also in the response to other stresses. For examples, *Hsf05* and *cHsp70*-5 showed a high up-regulation under four stresses except drought. The extent of overlapped genes response to two or more stresses among heat, cold, NaCl and PEG was examined and showed in the Venn diagram (Fig 1B). There were 6 genes (*Hsf09*, *Hsf14*, *Hsf20*, *Hsf22*, *cHsp70-1* and *mtHsp70-2*) that responded to all four stresses, indicating that these genes may be involved in multiple stresses responding. The number of overlapping responsive genes between heat stress and each of cold, drought and salt stresses were 12, 12 and 15, respectively, while the number of overlapping responsive genes between drought/cold, salt/cold and drought/salt were 6, 11 and 8, respectively. Moreover, a list of BdHsf and *BdHsp70* genes, including *Hsf02*, *Hsf03*, *Hsf15*, *cHsp70-2*, *cHsp70-6*, *cHsp70-11*, *cpHsp70-1*, *Bip1*, *Hsp110-2* and *Hsp110-8*, were only up-regulated under HS, suggesting these genes were specific heat stress-related. Although some pairs of paralogs were clustered together with similar expression patterns and exhibited redundant roles in response to stress, it was believed that at least several *Hsf* and *Hsp70* paralogs showed function diversity in signal network, such as *Hsf02/Hsf07*, *Hsf01/Hsf09*, *Bip2/Bip3* and *mtHsp01/mtHsp02*.

### Expression correlation of *Hsp70* and *Hsf* genes between any two of abiotic stresses

**Fig 6:**
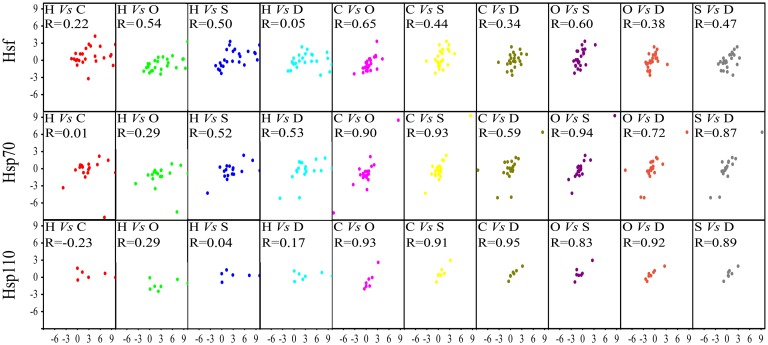
Expression correlations of *Hsf* and *Hsp70* genes between any two of heat, cold, drought, salt and oxidative stresses in *B*. *distachyon*. For each family of *Hsf* and *Hsp70* (*Hsp110s* were separately analyzed), responsive expression levels of gene members under any two stress were plotted. At each top right of subfigure, H stands for heat stress, C for cold stress, O for oxidative stress, D for drought stress, S for salt stress. For example, ‘H Vs C’ meant that x-axis and y-axis represented the response fold change under heat and cold stresses, respectively. Pearson correlation coefficient (R) represents the expression pattern similarity of a given gene family under two stresses.

Plant *Hsf* and *Hsp70* genes were well known as modulators implicated in a variety of biological processes [1,59]. Hsp70s, as molecular chaperones, were induced in response to various stresses in order to confer protection against such stressors. Hsfs, as transcriptional factors of Hsp70s, cooperated with Hsp70s to form a network responding to various stresses. Both of these two family members played a crucial role in improving plant tolerance in response to multiple environmental stresses apart from HS. Studying on the role of Hsfs and Hsp70s under HS and other stress conditions, such as cold, salt, drought and oxidative stress, may therefore provide insight into multiple stress tolerance mechanisms [59]. Therefore, we further compared gene expression patterns of *Hsfs* and *Hsp70s* responding five stresses mentioned above (Fig 6). Expression correlation of *Hsfs* and *Hsp70s* (*Hsp110s* subgroup was separately analyzed) between any two of heat, cold, salt, drought and oxidative stress has been shown in Fig 6. Pearson correlation coefficient (R) represented the degree of co-regulation between two stresses. For the ten stress pairs, *Hsf* and *Hsp70* gene families exhibited a good expression correlation under oxidative stress and other three stresses except under HS. This indicated that the similar response network existed between oxidative stress and other three stresses in seedling, implying that oxidative stress might be an accompanied stress when plant was expose to the other stressors. Hsp70 subfamily members had the similar expression pattern under eight of ten stress pairs, except under heat/cold and heat/ oxidative stress treatments, which was consistent with the that in rice [1]. Hsf family members showed a diversity of expression pattern under most of these ten stress pairs, suggesting that Hsfs act various roles responding to different stress. Other co-regulated response patterns were *Hsp70* subfamily and *Hsp110* subfamily under cold/salt, cold/drought, salt/drought stresses. Because the most of abiotic stress reports studied in the laboratory can not reflect the actual conditions occurred in the field, in which crops and other plants are routinely subjected to a combination of different abiotic stresses [76]. Investigating the correlated expression patterns under multiple stresses in *B*. *distachyon*, it might be of considerable importance for enhancing the tolerance of agriculturally important crop species to field conditions, which were a combination of several different types of stress, rather than just a single stress in isolation [76]. The response of Hsfs and Hsp70s to multiple abiotic stresses overlapped extensively, suggesting that Hsfs and Hsp70s were important in responding to multiple environmental stresses.

### Regulatory networks of *B*. *distachyon* Hsp 70s and Hsfs

**Fig 7:**
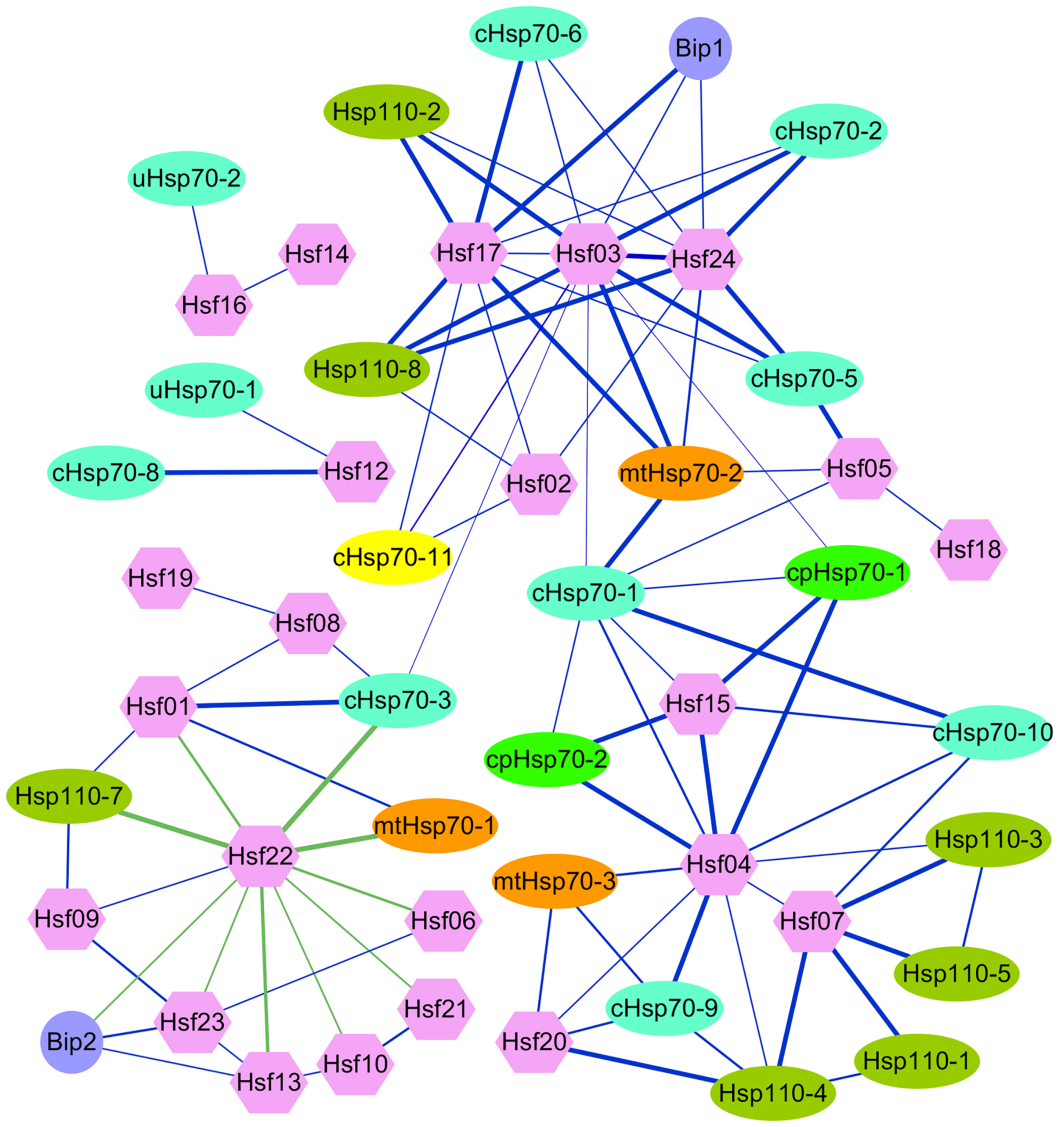
Co-expression networks of Hsfs and Hsp70s in *B*. *distachyon*. Nodes represent Hsfs and Hsp70s in *B*. *distachyon*, edges indicate pairwise correlation constructed by Cluster 3.0 software. Blue edges indicate the positive correlation and green edges indicate the negative correlation, while thin edges indicate moderate co-expressions and thick edges indicate strong co-expressions between the two nodes. The network picture which represented the co-expression regulatory network was created using Cytoscape.

In general, members of Hsp and Hsf families formed an expansive regulation network responding to multiple environmental stresses. The dramatic transcriptional up-regulation of Hsps performed chaperone function by helping to refold proteins which were damaged by environmental stresses, and was induced primarily by Hsf, which were binding to cis-acting sequences, designated as HSEs, in the promoter of Hsps [77,78]. On other hand, a physical interaction between Hsps and Hsfs formed a feedback loop led to repression of the activity of Hsfs at normal temperature [17]. To explore the potential regulatory network between BdHsfs and their downstream BdHsp70s, we constructed the co-expression regulatory network between *Hsfs* and *Hsp70s* upon different stress treatments by using hierarchical clustering analysis of expression data under abiotic stress for both *Hsf* and *Hsp70* genes at a time in *B*. *distachyon*. For investigating the co-expressed network, the expression data of *BdHsf* and *BdHsp70* genes were clustered together to form integrated expression profiles, which indicated that specific clusters of co-expressed *BdHsf* and *BdHsp70* genes were involved in response to a range of applied stress conditions. As shown in Fig 7, there were many co-expression relationships between *B*. *distachyon Hsfs* and *Hsp70s*. Results revealed that one *Hsf* gene could be simultaneously co-expressed with several *Hsp70* genes, suggesting that some of BdHsp70s, which performed same or different physiological function, might be regulated by same *BdHsf* gene. Moreover, six *BdHsfs* belonged five subgroups, such as A2 (*Hsf03* and *Hsf07*), A6 (*Hsf17*), B1 (*Hsf22*), B2 (*Hsf24*) and B4 (*Hsf04*), showed high co-expression levels with the most of *BdHsp70s*, indicating that these BdHsfs might be the key regulators among the 24 BdHsfs. As previously reported by Nishizawa *et al*, a large number of heat shock proteins were highly expressed in *HsfA2*-overexpressing Arabidopsis plants compared with those in the wild-type plants, suggesting that Arabidopsis HsfA2 is a key regulator in the induction of the defense system under environmental stresses [79]. In our study, *BdHsf03* (a member of class A2), which was highly expressed under HS, showed good co-expression correlation with 10 of 29 *BdHsp70s*, suggesting that BdHsf03 might play an important role in Hsp70s transcriptional activation. *BdHsf17* and *BdHsf24*, as well as *BdHsf03*, shared good co-expression correlation with a set of *BdHsp70s* under various stresses, indicating that these BdHsps were generally regulated by these three BdHsfs, which might be key regulators under multiple stresses in *B*. *distachyon*. Further, we found that chloroplast Hsp70, such as cpHsp70-1 and cpHsp70-2, were mainly regulated by BdHsf04 and BdHsf15, while most BdHsp110s were mostly regulated by BdHsf07, member of class A2. In plant, the heat stress response is finely regulated by activation and repression activities of Hsfs [80]. Generally, the expression of a particular BdHsfs could induce the expression of a different set of Hsp70s, implying an intricate transcriptional regulatory network between BdHsfs and BdHsp70s. In Arabidopsis, Ikeda *et al*. found that AtHsfB1 and AtHsfB2, members of class B, showed as transcriptional repressors and negatively regulated the expression of heat-inducible Hsfs and several heat shock protein genes [80]. Consistently, *BdHsf22*, a member of class B, showed a strong negative co-expression correlation with a set of *BdHsfs* (*BdHsf01*, *BdHsf06*, *BdHsf10*, *BdHsf13*, *BdHsf21*, and *BdHsf23*) and several *BdHsp70s* (*BdBip2*, *BdcHsp70-3*, *BdmtHsp70-1*, and *BdHsp110-7*), indicating that BdHsf22 might be a transcriptional repressors to negatively regulate the stress response in *B*. *distachyon*. It has been found few class B Hsfs were linked Hsp70s as a Hsf-Hsp70 pairs, for instance, Hsf04 (a member of HsfB4) and cHsp70-1 might be linked together to regulate the expression of *Hsf15*, *cHsp70-10*, *cpHsp70-1* and *cpHsp70-2*. Furthermore, predicted PPI network has been generated by STRING V10.0 software online, the results showed that BdHsp110-3, -4, -5 and -8 were related to the largest number of BdHsp70s (S4 Fig). Interestingly, BdcHsp70-1 and BdcHsp70-2 were interacted with several class B Hsfs, including BdHsf18, BdHsf23 and BdHsf24, suggesting that BdcHsp70-1 and BdcHsp70-2 might act as feedback regulators to suppress the signaling pathway of stress response by binding to class B Hsfs under non-HS conditions and in the attenuating period [80]. Moreover, members of class B Hsfs were correlated to a set of Hsfs (such as BdHsf01, BdHsf12, BdHsf15, and BdHsf21), indicating that Hsfs in class B serve as transcriptional repressors or coactivators that cooperate with class A Hsfs (S4 Fig) [81,82]. These results also revealed that the diagrammatic co-expression regulatory network and predicted PPI network could provide a possibility to deduce a possible signaling pathway of stress response in *B*. *distachyon*, which showed that the Hsp70s might be regulated by both the activation and the repression mechanisms. However, the accurate regulatory mechanisms among Hsfs and Hsps of herbaceous plants during development and stress responses required further investigation.

## Conclusions

Identification and characterization of *Hsf* and *Hsp70* genes in a grass model-species would help to better understand the evolutionary processes and functions of these gene families. In this study, 53 members of *B*. *distachyon Hsf* and *Hsp70* gene families were identified. Genetic characterizations analyses (phytogenesis, chromosomal localization, gene duplication, protein structure) and abiotic stresses induced expression profile have been systematically investigated. Phylogenetic tree revealed that BdHsfs and BdHsp70s can be divided into three and six subfamilies, respectively. Most of subfamilies contained members from rice, Arabidopsis and *B*. *distachyon*, suggesting that the functions of most of Hsfs and Hsp70s were conserved during evolution. In addition, gene duplication analysis implied that *Hsfs* and *Hsp70s* might be the result of genomic rearrangements and expansions during the process of evolution, for instance, five of *cHsp70s*, which were located at the end of chromosome I in tandem duplication region, might be originating from *BdcHsp70*-1 and/or *BdcHsp70*-6. A heat-induced expression profile showed that HS-induced *BdHsf* expression can induce the acquired-thermotolerance to prevent plant from severe HS challenge. Expression heatmap and correlation analysis of BdHsfs and BdHsp70s showed that the response of Hsfs and Hsp70s to multiple abiotic stresses exhibited extensively overlapped and distinct expression pattern, suggesting that some genes were important in responding to multiple environmental stresses, and others were stress specific response genes. Moreover, the co-expression network implied that there was a complex transcriptional regulatory network between *B*. *distachyon* Hsfs and Hsp70s, and BdHsp70s might be regulated by both the activation and the repression mechanisms. Our study provided genetic characterizations and expression analysis of *Hsfs* and *Hsp70s* genes in *B*. *distachyon* under multiple stresses conditions which could improved our understanding for further investigating the accurate regulatory mechanisms among Hsfs and Hsps in herbaceous plants.

## Supporting information

S1 Table List of *Hsf* and *Hsp70* genes in *B*. *distachyon*. (DOC)

S2 Table Characteristics of BdHsfs and BdHsp70s. (DOC)

S3 Table Expression data of *BdHsf* and *BdHsp70* genes after abiotic stresses. (DOC)

S4 Table The list of qRT-PCR primers of *BdHsf* and *BdHsp70* genes. (DOC)

S1 File The peptide sequences of Hsf and Hsp70 members in *B*. *distachyon*, *A*. *thaliana* and *O*. *sativa*. (DOC)

S1 Fig Sequence logos for the conserved motifs of Hsf (A) and Hsp70 (B) proteins in *B*. *distachyon*. (TIF)

S2 Fig Diagram of HS treatment protocol. (TIF)

S3 Fig The expression analysis of *BdWRKY36*, *BdCBF1*, *BdCBF2*, *BdP5CS1*, and *BdAPX1* under different stress treatments by real-time RT-PCR analysis. (TIF)

S4 Fig Predicted protein-protein interaction network of BdHsfs and BdHsp70s identified in *B*. *distachyon*. (TIF)
