# Supplementary material for: Genome-wide survey of heat shock factors and heat shock protein 70s and their regulatory network under abiotic stresses in Brachypodium distachyon
Source: PLoS One. 2017 Jul 6;12(7):e0180352. doi: 10.1371/journal.pone.0180352 (PMC5500289; doi:10.1371/journal.pone.0180352)
Supplement: S1 Table — (DOC) [file pone.0180352.s001.doc]

S1 Table A List of Hsf genes in *B. distachyon*.

| **Gene name** | **Locus** | **class** | **Intron** | **Chr** | **Location** | **a.a. Length** |
| --- | --- | --- | --- | --- | --- | --- |
| **BdHsf-01** | Bradi1g01130 | A1a | 2 | 1 | 755335 - 760135 | 621 |
| **BdHsf-02** | Bradi1g05550 | A2a | 1 | 1 | 3777047 - 3779557 | 347 |
| **BdHsf-03** | Bradi1g08891 | A2e | 1 | 1 | 6287441 - 6290883 | 385 |
| **BdHsf-04** | Bradi1g19900 | B4b | 1 | 1 | 15943546 - 15946059 | 313 |
| **BdHsf-05** | Bradi1g37720 | A7b | 1 | 1 | 33623352 - 33624526 | 348 |
| **BdHsf-06** | Bradi1g38140 | C2b | 1 | 1 | 34296765 - 34297797 | 264 |
| **BdHsf-07** | Bradi1g55630 | A2b | 1 | 1 | 54140758 - 54143900 | 457 |
| **BdHsf-08** | Bradi1g61620 | B4d | 1 | 1 | 60967400 - 60969340 | 300 |
| **BdHsf-09** | Bradi1g69407 | A8 | 1 | 1 | 67836946 - 67840540 | 393 |
| **BdHsf-10** | Bradi1g74350 | A6b | 2 | 1 | 71592465 - 71594730 | 340 |
| **BdHsf-11** | Bradi2g18980 | A4d | 2 | 2 | 16735523 - 16737272 | 470 |
| **BdHsf-12** | Bradi2g41530 | A7a | 1 | 2 | 41768002 - 41770456 | 380 |
| **BdHsf-13** | Bradi2g44050 | C1a | 2 | 2 | 44543658 - 44545038 | 335 |
| **BdHsf-14** | Bradi2g48990 | C1b | 1 | 2 | 49157121 - 49158267 | 247 |
| **BdHsf-15** | Bradi2g49860 | A4a | 1 | 2 | 49859810 - 49862297 | 438 |
| **BdHsf-16** | Bradi3g08870 | C2a | 1 | 3 | 6966824 - 6968528 | 314 |
| **BdHsf-17** | Bradi3g26920 | A6a | 1 | 3 | 27699259 - 27702662 | 356 |
| **BdHsf-18** | Bradi3g42130 | B2b | 1 | 3 | 43857313 - 43858451 | 345 |
| **BdHsf-19** | Bradi3g43710 | A5 | 1 | 3 | 45298993 - 45302044 | 468 |
| **BdHsf-20** | Bradi3g44700 | A3 | 2 | 3 | 46678236 - 46681202 | 481 |
| **BdHsf-21** | Bradi4g32050 | B4c | 1 | 4 | 37779171 - 37780616 | 404 |
| **BdHsf-22** | Bradi4g32130 | B1 | 1 | 4 | 37846831 - 37850530 | 302 |
| **BdHsf-23** | Bradi4g35780 | B2c | 1 | 4 | 41114697 - 41116660 | 399 |
| **BdHsf-24** | Bradi5g18680 | B2a | 1 | 5 | 21774260 - 21775504 | 307 |

S1 Table B List of Hsp70 genes in *B. distachyon*.

| **Gene Name** | **Locus** | **Group** | **Intron** | **Chr** | **Location** | **a.a. Length** |
| --- | --- | --- | --- | --- | --- | --- |
| **BdcHsp70-1** | Bradi1g66590 | I | 1 | 1 | 65554917-65557354 | 651 |
| **BdcHsp70-2** | Bradi2g23250 | I | 1 | 2 | 20785428-20789075 | 649 |
| **BdcHsp70-3** | Bradi1g03720 | I | 1 | 1 | 2509979-2514089 | 648 |
| **BdcHsp70-4** | Bradi4g04220 | I | 1 | 4 | 3416511-3420431 | 648 |
| **BdcHsp70-5** | Bradi2g54570 | I | 1 | 2 | 53215909-53219322 | 654 |
| **BdcHsp70-6** | Bradi1g66470 | I | 1 | 1 | 65475610-65479556 | 658 |
| **BdcHsp70-7** | Bradi1g66520 | I | 2 | 1 | 65509315-65511651 | 597 |
| **BdcHsp70-8** | Bradi1g66527 | I | 2 | 1 | 65514260-65516211 | 446 |
| **BdcHsp70-9** | Bradi1g66540 | I | 1 | 1 | 65521241-65525328 | 587 |
| **BdcHsp70-10** | Bradi1g66550 | I | 2 | 1 | 65532141-65535382 | 481 |
| **BdcHsp70-11** | Bradi1g66560 | I | 1 | 1 | 65317307-65327140 | 472 |
| **BduHsp70-1** | Bradi5g17520 | I | 2 | 5 | 20928722-20930144 | 141 |
| **BduHsp70-2** | Bradi2g46937 | I | 1 | 2 | 47116565-47121795 | 891 |
| **BdBip1** | Bradi3g01477 | II | 7 | 3 | 942486-946497 | 665 |
| **BdBip2** | Bradi2g06050 | II | 0 | 2 | 4552208-4554412 | 671 |
| **BdBip3** | Bradi4g28250 | II | 1 | 4 | 33475723-33477885 | 666 |
| **BdcpHsp70-1** | Bradi2g30560 | III | 7 | 2 | 29970721-29976931 | 684 |
| **BdcpHsp70-2** | Bradi4g39470 | III | 7 | 4 | 44013534-44019523 | 688 |
| **BdmtHsp70-1** | Bradi3g57450 | III | 5 | 3 | 56830508-56835760 | 681 |
| **BdmtHsp70-2** | Bradi1g77637 | III | 5 | 1 | 74120315-74124675 | 677 |
| **BdmtHsp70-3** | Bradi4g33878 | III | 4 | 4 | 39455295-39458393 | 680 |
| **BdHsp110-1** | Bradi4g43170 | IV | 1 | 4 | 46926508-46928269 | 454 |
| **BdHsp110-2** | Bradi1g69700 | V | 1 | 1 | 68409946-68413499 | 578 |
| **BdHsp110-3** | Bradi3g53100 | VI | 13 | 3 | 53635932-53642191 | 886 |
| **BdHsp110-4** | Bradi1g32770 | VI | 8 | 1 | 28264761-28270249 | 736 |
| **BdHsp110-5** | Bradi2g33676 | VI | 9 | 2 | 33563118-33568562 | 836 |
| **BdHsp110-6** | Bradi1g75681 | IV | 3 | 1 | 72759995-72761214 | 274 |
| **BdHsp110-7** | Bradi5g05900 | VI | 5 | 5 | 7472967-7488406 | 684 |
| **BdHsp110-8** | Bradi2g33682 | VI | 8 | 2 | 33570255-33576686 | 843 |
