# Supplementary material for: Genome-wide survey of heat shock factors and heat shock protein 70s and their regulatory network under abiotic stresses in Brachypodium distachyon
Source: PLoS One. 2017 Jul 6;12(7):e0180352. doi: 10.1371/journal.pone.0180352 (PMC5500289; doi:10.1371/journal.pone.0180352)
Supplement: S2 Table — (DOC) [file pone.0180352.s002.doc]

S2 Table A Characteristics of BdHsfs.

| **Gene Name** | **DBD** | **HR-A/B** | **NLS** | **NES** | **AHA** | **PI** | **MW** | **Subcellular Localization** |
| --- | --- | --- | --- | --- | --- | --- | --- | --- |
| **BdHsf-01** | 48-138 | 183-233 | NKKRR(257) |  | DSFWEQFL(468) | 4.92 | 67716.32 | Nuclear |
| **BdHsf-02** | 36-127 | 155-205 | MRKELEDAISNKRRRR(220) | LKRDKQLLM(155) | DDFWEDLLHQ(311) | 5.50 | 39021.62 | Nuclear |
| **BdHsf-03** | 55-145 | 180-230 | SKKRR(268) | LGL(334) | ESFWMELLSL(325) | 5.49 | 41598.08 | Nuclear |
| **BdHsf-04** | 36-128 | 208-237 | TKRAR(296) |  |  | 6.68 | 34736.96 | Nuclear |
| **BdHsf-05** | 49-139 | 167-217 | KKKRK(242) |  | MVWYELLGEE(292) | 4.69 | 38014.59 | Chloroplast/Cytoplasmic |
| **BdHsf-06** | 13-113 | 144-180 | EKRAR(206) | LTV(177) |  | 6.22 | 28332.74 | Chloroplast |
| **BdHsf-07** | 55-145 | 176-266 | MRKQLHDTISKKRRRR(241) |  | DNFWDDLLNE(414) | 4.88 | 50304.49 | Nuclear |
| **BdHsf-08** | 33-125 | 190-219 | SKKRR(273) |  |  | 6.00 | 34051.18 | Nuclear |
| **BdHsf-09** | 52-144 | 181-231 | NKKRR(256) | LKRDKALLMQQL(181) | DWDIELFFDD(345) | 4.83 | 43389.32 | Nuclear |
| **BdHsf-10** | 40-130 | 161-211 | LKRRR(233) |  | DDFWEELLSE(310) | 5.25 | 38973.15 | Nuclear/Cytoplasmic |
| **BdHsf-11** | 123-214 | 243-292 | SKKRR(312) | LALVSL(364) |  | 8.45 | 51978.41 | Nuclear |
| **BdHsf-12** | 74-166 | 194-244 | VKRQR(267) |  | QQIWLNYGN(251) | 5.94 | 42777.24 | Nuclear/Cytoplasmic |
| **BdHsf-13** | 21-112 | 168-204 | KKRQR(220) |  |  | 5.23 | 36787.18 | Nuclear |
| **BdHsf-14** | 25-117 | 141-177 | QKRRR(208) |  |  | 9.07 | 26933.61 | Chloroplast/Cytoplasmic |
| **BdHsf-15** | 13-103 | 133-183 | RKKRR(200) |  | DGFWQQFLTE(375) | 5.39 | 48860.88 | Nuclear |
| **BdHsf-16** | 17-109 | 143-179 | VKRPR(223) |  |  | 6.00 | 33332.93 | Nuclear |
| **BdHsf-17** | 40-131 | 161-211 | SKKRRR(235) |  | NEFWAELFDD(312) | 5.16 | 40236.04 | Nuclear |
| **BdHsf-18** | 18-109 | 184-213 | RKRMR(279) |  |  | 8.10 | 36641.45 | Nuclear |
| **BdHsf-19** | 21-112 | 139-189 | HKKRR(213) | LSLTL(338) | DKFWEQFLTE(417) | 4.93 | 51677.42 | Nuclear |
| **BdHsf-20** | 73-165 | 199-249 | KRKFLK(273) | LDDGDLHL(455) | EKFWELDFED(445) | 5.66 | 53183.02 | Nuclear |
| **BdHsf-21** | 27-120 | 225-254 | ARKK(276) | LLLECDDLSL(378) |  | 8.97 | 42982.25 | Nuclear |
| **BdHsf-22** | 29-120 | 185-214 | AKRAR(265) | VRQLDLRLLM(216) |  | 9.82 | 32790.17 | Nuclear |
| **BdHsf-23** | 44-135 | 224-253 | QKRSR(321) |  |  | 5.05 | 41873.27 | Nuclear |
| **BdHsf-24** | 11-102 | 163-192 | SKRSR(220) | LNVLAL(268) |  | 5.29 | 32975.19 | Nuclear |

S2 Table B Characteristics of BdHsp70s.

| **Gene Name** | **ATPase Domain, NBD** | **Peptide Binding Domain, SBD** | **C-terminal Lid** | **PI** | **MW** | **Subcellular Localization** |
| --- | --- | --- | --- | --- | --- | --- |
| **BdcHsp70-1** | 9-387 | 392-549 | 543-622 | 5.08 | 71294.61 | Cytoplasmic |
| **BdcHsp70-2** | 8-386 | 391-548 | 542-620 | 5.16 | 71065.58 | Cytoplasmic |
| **BdcHsp70-3** | 10-388 | 393-550 | 544-623 | 5.08 | 71299.8 | Cytoplasmic |
| **BdcHsp70-4** | 9-387 | 392-549 | 543-622 | 5.07 | 71266.72 | Cytoplasmic |
| **BdcHsp70-5** | 9-387 | 392-549 | 543-622 | 5.17 | 71444.15 | Cytoplasmic |
| **BdcHsp70-6** | 15-393 | 399-555 | 549-627 | 5.18 | 72054.42 | Cytoplasmic |
| **BdcHsp70-7** | 11-362 | 368-519 |  | 6.61 | 66362.2 | Cytoplasmic |
| **BdcHsp70-8** | 9-377 | 383-440 |  | 8.01 | 48896.14 | Cytoplasmic/Chloropalst |
| **BdcHsp70-9** | 11-391 | 397-544 |  | 8.48 | 64823.75 | Cytoplasmic/Mitochondrial |
| **BdcHsp70-10** | 11-320 | 325-472 |  | 5.11 | 52358.94 | Cytoplasmic |
| **BdcHsp70-11** | 14-314 | 319-467 |  | 5.14 | 52075.55 | Cytoplasmic |
| **BduHsp70-1** | 8-59 |  |  | 8.87 | 15707.36 | Cytoplasmic/Mitochondrial |
| **BduHsp70-2** | 280-651 | 673-828 |  | 8.63 | 96544.71 | Cytoplasmic/Nuclear |
| **BdBip1** | 33-407 | 416-572 | 566-649 | 5.09 | 73359.07 | ER |
| **BdBip2** | 43-422 | 429-585 | 579-651 | 5.28 | 73321.49 | Cytoplasmic/ER |
| **BdBip3** | 41-421 | 430-584 | 578-651 | 5.18 | 72597.77 | ER |
| **BdcpHsp70-1** | 46-422 | 424-580 | 574-651 | 5.04 | 73076.54 | Chloroplast |
| **BdcpHsp70-2** | 49-425 | 427-583 | 550-649 | 5.04 | 73168.82 | Chloroplast |
| **BdmtHsp70-1** | 51-427 | 429-585 | 578-647 | 5.66 | 73117.88 | Mitochondrial |
| **BdmtHsp70-2** | 53-427 | 429-585 | 578-647 | 5.47 | 72907.59 | Mitochondrial |
| **BdmtHsp70-3** | 54-428 | 430-586 | 579-648 | 5.65 | 72478.11 | Mitochondrial |
| **BdHsp110-1** | 43-421 |  |  | 7.67 | 49828.02 | Chloroplast |
| **BdHsp110-2** | 29-414 | 429-529 |  | 5.8 | 62441.92 | Cytoplasmic/Mitochondrial |
| **BdHsp110-3** | 27-413 | 423-554,598-627 | 660-741 | 5.32 | 97637.12 | Cytoplasmic |
| **BdHsp110-4** | 2-381 | 384-490,520-572 | 541-638 | 5.47 | 81835.88 | Cytoplasmic |
| **BdHsp110-5** | 2-381 | 384-491,563-615 | 584-681,689-783 | 5.08 | 92728.15 | Cytoplasmic |
| **BdHsp110-6** | 51-274 |  |  | 9.17 | 29766.18 | Mitochondrial |
| **BdHsp110-7** | 36-442 | 453-559 |  | 6.34 | 76321.43 | Cytoplasmic |
| **BdHsp110-8** | 2-381 | 384-491,571-622 | 589-688,696-790 | 5.07 | 93343.71 | Cytoplasmic |
