# Supplementary material for: Genome-wide survey of heat shock factors and heat shock protein 70s and their regulatory network under abiotic stresses in Brachypodium distachyon
Source: PLoS One. 2017 Jul 6;12(7):e0180352. doi: 10.1371/journal.pone.0180352 (PMC5500289; doi:10.1371/journal.pone.0180352)
Supplement: S3 Table — (DOC) [file pone.0180352.s003.doc]

S3 Table A Expression data of Hsf genes under HS challenge in *B. distachyon*.

| **Gene Name** | **Group I** | | |  | **Group II** | | |  | **Group III** | | |  | **Group IV** | | |
| --- | --- | --- | --- | --- | --- | --- | --- | --- | --- | --- | --- | --- | --- | --- | --- |
|  | fold-change | p-value | up/down |  | fold-change | p-value | up/down |  | fold-change | p-value | up/down |  | fold-change | p-value | up/down |
| **Hsf01** | 0.7320428 | 0.0421 |  |  | 0.838956 | 0.1220 |  |  | 0.649169 | 0.2203 |  |  | 0.591862 | 0.0219 |  |
| **Hsf02** | 28.11627 | 0.0001 | up |  | 1.159364 | 0.0896 |  |  | 4.150639 | 0.0023 | up |  | 0.972655 | 0.2889 |  |
| **Hsf03** | 643.59085 | 0.0000 | up |  | 6.711646 | 0.0126 | up |  | 206.5003 | 0.0000 | up |  | 18.80877 | 0.0003 | up |
| **Hsf04** | 1.2923528 | 0.5049 |  |  | 0.840896 | 0.7263 |  |  | 1.643381 | 0.3205 |  |  | 0.950439 | 0.6902 |  |
| **Hsf05** | 968.76303 | 0.0000 | up |  | 13.64216 | 0.0050 | up |  | 546.2181 | 0.0000 | up |  | 42.6163 | 0.0000 | up |
| **Hsf06** | 1.6021398 | 0.4446 |  |  | 0.170361 | 0.0401 | down |  | 0.890899 | 0.8228 |  |  | 0.176369 | 0.0313 |  |
| **Hsf07** | 2.5256709 | 0.0000 | up |  | 0.537126 | 0.0884 |  |  | 0.82932 | 0.0139 |  |  | 1.191958 | 0.0001 |  |
| **Hsf08** | 0.9096184 | 0.6975 |  |  | 0.239816 | 0.0204 | down |  | 0.275476 | 0.0052 | down |  | 0.295248 | 0.0111 | down |
| **Hsf09** | 23.80732 | 0.0000 | up |  | 2.12874 | 0.0003 | up |  | 3.038445 | 0.0000 | up |  | 1.624505 | 0.0003 |  |
| **Hsf10** | 61.109863 | 0.0005 | up |  | 1.375542 | 0.0087 |  |  | 7.691864 | 0.0000 | up |  | 1.802501 | 0.3559 |  |
| **Hsf11** | 0.6861837 | 0.2298 |  |  | 0.239816 | 0.0793 |  |  | 0.658231 | 0.1475 |  |  | 0.408951 | 0.1341 |  |
| **Hsf12** | 4.0185267 | 0.0016 | up |  | 1.443929 | 0.0117 |  |  | 2.123828 | 0.0040 | up |  | 2.744735 | 0.0013 | up |
| **Hsf13** | 0.5236471 | 0.3484 |  |  | 0.82169 | 0.8489 |  |  | 0.705475 | 0.4506 |  |  | 0.467596 | 0.3700 |  |
| **Hsf14** | 2.4060501 | 0.0230 | up |  | 0.716978 | 0.9124 |  |  | 1.189207 | 0.5310 |  |  | 1.081725 | 0.1687 |  |
| **Hsf15** | 23.752377 | 0.0000 | up |  | 0.585064 | 0.1078 |  |  | 2.7007 | 0.0002 |  |  | 0.273573 | 0.0002 | down |
| **Hsf16** | 4.5315355 | 0.0001 | up |  | 0.056458 | 0.0701 |  |  | 1.259921 | 0.6209 |  |  | 1.512219 | 0.0000 |  |
| **Hsf17** | 397.09306 | 0.0000 | up |  | 7.656402 | 0.0001 | up |  | 53.19923 | 0.0000 | up |  | 2.356534 | 0.0005 | up |
| **Hsf18** | 121.37549 | 0.0001 | up |  | 1.470867 | 0.1247 |  |  | 18.54982 | 0.0019 | up |  | 1.225468 | 0.4028 |  |
| **Hsf19** | 0.8929595 | 0.3794 |  |  | 0.735433 | 0.7821 |  |  | 0.643197 | 0.0022 |  |  | 0.93088 | 0.2658 |  |
| **Hsf20** | 7.7274906 | 0.0001 | up |  | 2.394957 | 0.0009 | up |  | 6.468061 | 0.0002 | up |  | 3.271608 | 0.0002 | up |
| **Hsf21** | 7.0128458 | 0.0010 | up |  | 0.287175 | 0.2072 |  |  | 0.037508 | 0.0006 | down |  | 3.348078 | 0.0006 | up |
| **Hsf22** | 10.678719 | 0.0048 | up |  | 0.484085 | 0.3784 |  |  | 1.725084 | 0.3253 |  |  | 0.713672 | 0.8433 |  |
| **Hsf23** | 0.3223426 | 0.0000 | down |  | 0.160799 | 0.0003 | down |  | 0.4954 | 0.0107 | down |  | 0.457973 | 0.0149 | down |
| **Hsf24** | 452.98935 | 0.0000 | up |  | 4.356995 | 0.0068 | up |  | 51.98415 | 0.0001 | up |  | 2.19365 | 0.0191 | up |

S3 Table B Expression data of Hsp70 genes under HS challenge in *B. distachyon*.

| **Gene Name** | **Group I** | | |  | **Group II** | | |  | **Group III** | | |  |  | **Group IV** |  |
| --- | --- | --- | --- | --- | --- | --- | --- | --- | --- | --- | --- | --- | --- | --- | --- |
|  | fold-change | p-value | up/down |  | fold-change | p-value | up/down |  | fold-change | p-value | up/down |  | fold-change | p-value | up/down |
| **cHsp70-1** | 53.941859 | 0.0000 | up |  | 2.089754 | 0.0012 | up |  | 11.63178 | 0.0000 | up |  | 0.802923 | 0.7699 |  |
| **cHsp70-2** | 3532.9722 | 0.0000 | up |  | 19.29293 | 0.0007 | up |  | 239.9628 | 0.0000 | up |  | 49.52208 | 0.0000 | up |
| **cHsp70-3** | 1.5404302 | 0.0015 |  |  | 0.737135 | 0.4933 |  |  | 0.581023 | 0.0003 |  |  | 0.571701 | 0.0278 |  |
| **cHsp70-4** | 2.4566063 | 0.0000 | up |  | 0.737135 | 0.5951 |  |  | 0.907519 | 0.3892 |  |  | 1.180993 | 0.0008 |  |
| **cHsp70-5** | 7912.9504 | 0.0000 | up |  | 170.4652 | 0.0000 | up |  | 760.0761 | 0.0000 | up |  | 48.61513 | 0.0000 | up |
| **cHsp70-6** | 1021.6368 | 0.0000 | up |  | 24.93327 | 0.0001 | up |  | 216.2665 | 0.0000 | up |  | 32.14821 | 0.0000 | up |
| **cHsp70-7** | 3.0384453 | 0.0337 | up |  | 0.117169 | 0.0021 | down |  | 2.434007 | 0.0068 | up |  | 0.530957 | 0.4026 |  |
| **cHsp70-8** | 4.1219681 | 0.0000 | up |  | 0.963707 | 0.0214 |  |  | 0.90125 | 0.2743 |  |  | 0.511687 | 0.0062 |  |
| **cHsp70-9** | 1.6586391 | 0.0049 |  |  | 0.222725 | 0.0015 | down |  | 1.117287 | 0.5985 |  |  | 0.140958 | 0.0002 | down |
| **cHsp70-10** | 6.6345564 | 0.0002 | up |  | 0.332171 | 0.1871 |  |  | 0.986233 | 0.9628 |  |  | 1.023374 | 0.5754 |  |
| **cHsp70-11** | 138.14121 | 0.0000 | up |  | 30.48442 | 0.0000 | up |  | 112.986 | 0.0000 | up |  | 1.60956 | 0.2148 |  |
| **uHsp70-1** | 0.073133 | 0.0015 | down |  | 0.147624 | 0.0082 |  |  | 0.047476 | 0.0006 | down |  | 0.242603 | 0.0487 | down |
| **uHsp70-2** | 0.7022224 | 0.2891 |  |  | 0.289841 | 0.0124 | down |  | 0.629961 | 0.2558 |  |  | 0.476319 | 0.1403 |  |
| **Bip1** | 7.498836 | 0.0000 | up |  | 0.909618 | 0.2749 |  |  | 3.563595 | 0.0001 | up |  | 3.340352 | 0.0005 | up |
| **Bip2** | 2.6026837 | 0.0032 | up |  | 0.274841 | 0.0106 | down |  | 1.200249 | 0.5949 |  |  | 0.197967 | 0.1219 |  |
| **Bip3** | N/A | N/A |  |  | 4299.64 | 0.0000 | up |  | 433.5336 | 0.0001 | up |  | 448.8221 | 0.0000 | up |
| **cpHsp70-1** | 38.407957 | 0.0000 | up |  | 2.763826 | 0.0021 | up |  | 4.169863 | 0.0001 | up |  | 4.3873 | 0.0000 | up |
| **cpHsp70-2** | 8.8560703 | 0.0000 | up |  | 1.025741 | 0.0558 |  |  | 1.713168 | 0.0002 |  |  | 1.3692 | 0.0006 |  |
| **mtHsp70-1** | 2.1584565 | 0.0037 | up |  | 0.582367 | 0.3539 |  |  | 0.754364 | 0.0935 |  |  | 0.80107 | 0.8183 |  |
| **mtHsp70-2** | 276.92151 | 0.0000 | up |  | 15.70697 | 0.0009 | up |  | 25.57508 | 0.0003 | up |  | 21.55574 | 0.0003 | up |
| **mtHsp70-3** | 7.8898616 | 0.0000 | up |  | 0.981686 | 0.0198 |  |  | 1.52979 | 0.0220 |  |  | 1.453973 | 0.0002 |  |
| **Hsp110-1** | 4.616075 | 0.0056 | up |  | 0.866537 | 0.0222 |  |  | 0.825496 | 0.2641 |  |  | N/A | N/A |  |
| **Hsp110-2** | 1000.6118 | 0.0000 | up |  | 47.17661 | 0.0000 | up |  | 111.1733 | 0.0000 | up |  | 117.241 | 0.0000 | up |
| **Hsp110-3** | 2.3619853 | 0.0124 | up |  | 1.628263 | 0.4221 |  |  | 1.457336 | 0.0455 |  |  | 1.41095 | 0.5378 |  |
| **Hsp110-4** | 1.0328757 | 0.8852 |  |  | 0.773782 | 0.0042 |  |  | 0.705475 | 0.0285 |  |  | 1.114709 | 0.2065 |  |
| **Hsp110-5** | 7.3955973 | 0.0003 | up |  | 0.903335 | 0.0054 |  |  | 1.24545 | 0.0020 |  |  | 1.009285 | 0.0005 |  |
| **Hsp110-6** | N/A | N/A |  |  | 0.124712 | 0.0966 |  |  | 3.309622 | 0.0013 | up |  | 5.133704 | 0.0003 | up |
| **Hsp110-7** | 0.9704102 | 0.4087 |  |  | 0.679871 | 0.8403 |  |  | 1.021012 | 0.6846 |  |  | 1.512219 | 0.0114 |  |
| **Hsp110-8** | 150.81819 | 0.0000 | up |  | 7.429851 | 0.0001 | up |  | 16.07411 | 0.0000 | up |  | 5.265851 | 0.0000 | up |

S3 Table C Expression data of Hsf genes under multiple abiotic stresses in *B. distachyon*.

| **Gene Name** | **Cold** | | | **H_2_O_2_** | | | **NaCl** | | | **PEG** | | | **Heat** | | |
| --- | --- | --- | --- | --- | --- | --- | --- | --- | --- | --- | --- | --- | --- | --- | --- |
|  | fold-change | p-value | up/down | fold-change | p-value | up/down | fold-change | p-value | up/down | fold-change | p-value | up/down | fold-change | p-value | up/down |
| **Hsf01** | 0.990801 | 0.7718 |  | 0.461158 | 0.0006 | down | 0.732043 | 0.0181 |  | 0.721965 | 0.0025 |  | 0.732042 | 0.0421 |  |
| **Hsf02** | 0.911722 | 0.8244 |  | 0.747425 | 0.1209 |  | 1.620756 | 0.0573 |  | 1.905276 | 0.0993 |  | 28.11627 | 0.0001 | up |
| **Hsf03** | 0.535887 | 0.4128 |  | 0.387786 | 0.2490 |  | 1.064370 | 0.8297 |  | 0.243164 | 0.0504 |  | 643.5908 | 0.0000 | up |
| **Hsf04** | 0.974905 | 0.9710 |  | 0.278034 | 0.0694 |  | 0.487452 | 0.1880 |  | 0.683020 | 0.3393 |  | 1.292352 | 0.5049 |  |
| **Hsf05** | 6.821079 | 0.0004 | up | 9.871938 | 0.0207 | up | 6.468061 | 0.0004 | up | 0.597358 | 0.5272 |  | 968.7630 | 0.0000 | up |
| **Hsf06** | 2.168454 | 0.1561 | up | 0.285191 | 0.0575 |  | 1.042466 | 0.9497 |  | 0.429283 | 0.1600 |  | 1.602139 | 0.4446 |  |
| **Hsf07** | 0.801070 | 0.0179 |  | 0.416580 | 0.0000 | down | 0.850667 | 0.0659 |  | 0.913831 | 0.1100 |  | 2.525670 | 0.0000 | up |
| **Hsf08** | 0.558644 | 0.1816 |  | 0.221698 | 0.0049 | down | 0.207809 | 0.0048 | down | 0.629961 | 0.1018 |  | 0.909618 | 0.6975 |  |
| **Hsf09** | 18.67884 | 0.0000 | up | 1.167428 | 0.3664 |  | 2.188587 | 0.0000 | up | 2.032610 | 0.0016 | up | 23.80732 | 0.0000 | up |
| **Hsf10** | 5.502167 | 0.0003 | up | 1.761332 | 0.7301 |  | 0.571701 | 0.0078 |  | 0.892960 | 0.0468 |  | 61.10986 | 0.0005 | up |
| **Hsf11** | 2.313376 | 0.0098 | up | 0.658231 | 0.1009 |  | 0.302149 | 0.0045 | down | 0.835088 | 0.4664 |  | 0.686183 | 0.2298 |  |
| **Hsf12** | 1.164734 | 0.1453 |  | 0.880666 | 0.0816 |  | 6.483023 | 0.0001 | up | 5.051342 | 0.0010 | up | 4.018526 | 0.0016 | up |
| **Hsf13** | 1.159364 | 0.3473 |  | 0.414660 | 0.0125 | down | 0.343885 | 0.1282 |  | 0.280616 | 0.0013 | down | 0.523647 | 0.3484 |  |
| **Hsf14** | 2.168454 | 0.0207 | up | 0.979420 | 0.4346 |  | 3.723519 | 0.0103 | up | 1.874709 | 0.0792 |  | 2.406050 | 0.0230 | up |
| **Hsf15** | 1.647182 | 0.0010 |  | 0.512871 | 0.0002 |  | 0.821690 | 0.0703 |  | 0.882703 | 0.0177 |  | 23.75237 | 0.0000 | up |
| **Hsf16** | 4.247655 | 0.0135 | up | 1.470867 | 0.4469 |  | 9.940603 | 0.0000 | up | 1.307369 | 0.8197 |  | 4.531535 | 0.0001 | up |
| **Hsf17** | 1.681793 | 0.0069 |  | 1.170128 | 0.8241 |  | 2.572789 | 0.0021 | up | 2.608704 | 0.0022 | up | 397.0930 | 0.0000 | up |
| **Hsf18** | 1.334840 | 0.3506 |  | 0.412748 | 0.0732 |  | 2.982799 | 0.0270 | up | 0.163799 | 0.0628 |  | 121.3754 | 0.0001 | up |
| **Hsf19** | 1.081725 | 0.5241 |  | 0.662810 | 0.0022 |  | 0.528509 | 0.0253 |  | 1.283426 | 0.7388 |  | 0.892959 | 0.3794 |  |
| **Hsf20** | 7.029068 | 0.0001 | up | 0.341510 | 0.0006 | down | 3.010493 | 0.0008 | up | 2.178497 | 0.0337 | up | 7.727490 | 0.0001 | up |
| **Hsf21** | 0.108067 | 0.1625 |  | 0.190782 | 0.1683 |  | 0.905425 | 0.8704 |  | 0.773782 | 0.7012 |  | 7.012845 | 0.0010 | up |
| **Hsf22** | 5.643799 | 0.0098 | up | 1.529790 | 0.4911 |  | 3.837056 | 0.0208 | up | 3.784231 | 0.1406 |  | 10.67871 | 0.0048 | up |
| **Hsf23** | 1.225468 | 0.0102 |  | 0.270431 | 0.0000 | down | 0.527289 | 0.0062 |  | 0.271057 | 0.0145 | down | 0.322342 | 0.0000 | down |
| **Hsf24** | 1.977028 | 0.4303 |  | 1.194715 | 0.9791 |  | 2.313376 | 0.0297 | up | 1.265757 | 0.9177 |  | 452.9893 | 0.0000 | up |

S3 Table D Expression data of Hsp70 genes under multiple abiotic stresses in *B. distachyon*.

| **Gene Name** | **Cold** | | | **H_2_O_2_** | | | **NaCl** | | | **PEG** | | | **Heat** | | |
| --- | --- | --- | --- | --- | --- | --- | --- | --- | --- | --- | --- | --- | --- | --- | --- |
|  | fold-change | p-value | up/down | fold-change | p-value | up/down | fold-change | p-value | up/down | fold-change | p-value | up/down | fold-change | p-value | up/down |
| **cHsp70-1** | 4.33691 | 0.0001 | up | 1.58374 | 0.0488 |  | 4.95883 | 0.0001 | up | 3.34808 | 0.0005 | up | 53.9418 | 0.0000 | up |
| **cHsp70-2** | 0.29186 | 0.0222 | down | 0.96148 | 0.3806 |  | 0.52003 | 0.0448 |  | 0.61700 | 0.1116 |  | 3532.97 | 0.0000 | up |
| **cHsp70-3** | 1.13813 | 0.0557 |  | 0.43528 | 0.0002 | down | 0.72363 | 0.0020 |  | 0.83896 | 0.0047 |  | 1.54043 | 0.0015 |  |
| **cHsp70-4** | 1.25992 | 0.0027 |  | 0.72363 | 0.0034 |  | 1.36289 | 0.0069 |  | 2.43401 | 0.0001 | up | 2.45660 | 0.0000 | up |
| **cHsp70-5** | 2.15348 | 0.0010 | up | 4.28709 | 0.0004 | up | 2.82843 | 0.0396 | up | 1.71713 | 0.2085 |  | 7912.95 | 0.0000 | up |
| **cHsp70-6** | 0.59186 | 0.0017 |  | 0.49655 | 0.0002 | down | 0.77200 | 0.0302 |  | 1.08925 | 0.7830 |  | 1021.63 | 0.0000 | up |
| **cHsp70-7** | 0.59460 | 0.5570 |  | 0.56383 | 0.1445 |  | 0.42730 | 0.2475 |  | 2.27626 | 0.1846 |  | 3.03844 | 0.0337 | up |
| **cHsp70-8** | 0.34949 | 0.0005 | down | 0.47303 | 0.0011 |  | 1.32562 | 0.0826 |  | 2.17850 | 0.0007 | up | 4.12196 | 0.0000 | up |
| **cHsp70-9** | 1.48452 | 0.0581 |  | 0.25822 | 0.0001 | down | 0.39685 | 0.0006 | down | 0.73204 | 0.0935 |  | 1.65863 | 0.0049 |  |
| **cHsp70-10** | 0.57834 | 0.3970 |  | 0.33915 | 0.0980 |  | 0.51169 | 0.1641 |  | 0.54588 | 0.1413 |  | 6.63455 | 0.0002 | up |
| **cHsp70-11** | 0.00262 | 0.0000 | down | 0.00465 | 0.0000 | down | N/A | N/A |  | 0.83124 | 0.0097 |  | 138.141 | 0.0000 | up |
| **uHsp70-1** | 0.09473 | 0.0798 |  | 0.14392 | 0.0068 | down | 0.05065 | 0.0271 | down | 0.02950 | 0.0045 | down | 0.07313 | 0.0015 | down |
| **uHsp70-2** | 0.82169 | 0.5628 |  | 0.40990 | 0.0064 | down | 1.18099 | 0.6700 |  | 0.39777 | 0.0072 | down | 0.70222 | 0.2891 |  |
| **Bip1** | 0.86654 | 0.3439 |  | 0.71367 | 0.0095 |  | 0.89296 | 0.3507 |  | 1.05214 | 0.4704 |  | 7.49883 | 0.0000 | up |
| **Bip2** | 1.16205 | 0.4487 |  | 0.07856 | 0.0019 | down | 0.26609 | 0.0071 | down | 0.03132 | 0.0013 | down | 2.60268 | 0.0032 | up |
| **Bip3** | 356.230 | 0.0055 | up | 357.054 | 0.0247 | up | 618.801 | 0.0039 | up | 83.4785 | 0.0029 | up | N/A | N/A |  |
| **cpHsp70-1** | 1.56917 | 0.0049 |  | 0.51763 | 0.0016 |  | 0.69576 | 0.0137 |  | 0.81979 | 0.0215 |  | 38.4079 | 0.0000 | up |
| **cpHsp70-2** | 1.06191 | 0.3626 |  | 0.34949 | 0.0003 | down | 0.49769 | 0.0002 | down | 0.74915 | 0.0279 |  | 8.85607 | 0.0000 | up |
| **mtHsp70-1** | 1.43065 | 0.0332 |  | 0.60012 | 0.0075 |  | 0.86254 | 0.2889 |  | 1.12246 | 0.7198 |  | 2.15845 | 0.0037 | up |
| **mtHsp70-2** | 2.66352 | 0.0224 | up | 1.32562 | 0.7694 |  | 2.66968 | 0.0265 | up | 3.85483 | 0.0149 | up | 276.921 | 0.0000 | up |
| **mtHsp70-3** | 1.64338 | 0.0003 |  | 0.73884 | 0.0007 |  | 1.05946 | 0.6907 |  | 1.32562 | 0.1670 |  | 7.88986 | 0.0000 | up |
| **Hsp110-1** | N/A | N/A |  | 0.18903 | 0.0174 |  | N/A | N/A |  | 0.80292 | 0.0030 |  | 4.61607 | 0.0056 | up |
| **Hsp110-2** | 1.00000 | 0.7752 |  | 0.49769 | 0.2076 |  | 1.25992 | 0.0245 |  | 1.20860 | 0.0959 |  | 1000.61 | 0.0000 | up |
| **Hsp110-3** | 1.89649 | 0.0162 |  | 0.34628 | 0.0140 | down | 2.51984 | 0.1387 | up | 1.54756 | 0.6023 |  | 2.36198 | 0.0124 | up |
| **Hsp110-4** | 0.72699 | 0.1440 |  | 0.24599 | 0.0824 | down | 0.55994 | 0.0006 |  | 0.61273 | 0.0034 |  | 1.03287 | 0.8852 |  |
| **Hsp110-5** | 1.01162 | 0.8502 |  | 0.33837 | 0.0191 | down | 1.35974 | 0.0005 |  | 1.15136 | 0.0099 |  | 7.39559 | 0.0003 | up |
| **Hsp110-6** | 9.02129 | 0.0001 | up | 6.04887 | 0.0229 | up | 7.74537 | 0.0008 | up | 3.85483 | 0.0019 | up | N/A | N/A |  |
| **Hsp110-7** | 3.01746 | 0.0000 | up | 0.97041 | 0.2233 |  | 1.60214 | 0.0018 |  | 2.20891 | 0.0015 | up | 0.97041 | 0.4087 |  |
| **Hsp110-8** | 1.62450 | 0.0006 |  | 0.80107 | 0.0011 |  | 1.30134 | 0.0053 |  | 1.82344 | 0.0038 |  | 150.818 | 0.0000 | up |
