# Supplementary material for: Genome-wide survey of heat shock factors and heat shock protein 70s and their regulatory network under abiotic stresses in Brachypodium distachyon
Source: PLoS One. 2017 Jul 6;12(7):e0180352. doi: 10.1371/journal.pone.0180352 (PMC5500289; doi:10.1371/journal.pone.0180352)
Supplement: S4 Table — (DOC) [file pone.0180352.s004.doc]

S4 Table The list of qRT-PCR primers

| **Gene name** | **Forward Primer** | **Reward Primer** |
| --- | --- | --- |
| **BdHsf-01** | CTGGAGGGAGCTTTGAAATG | GCCCATCCATTATCCAGTTG |
| **BdHsf-02** | ACTGAGCAGAAGCAACAGCA | ACGGAGAGCCATTGATGAAC |
| **BdHsf-03** | AGCTTACTACGCAGCGGAAG | GAAGTTGCCGTGCTTGAAGT |
| **BdHsf-04** | GCTTTCCTTGTGGAGAGGTG | AGCTGGAGAAGTTGTTGTGC |
| **BdHsf-05** | ACCCGAACTTCCTGGACAA | CAGCTCGTACCACACCATGT |
| **BdHsf-06** | GATGCTAGCCTTCCTCCTCA | GAAACCAAACGAGGAACCAG |
| **BdHsf-07** | CTTGAGGTAGGGCACTTTGG | GGAGTTGCTTCCTCATCTCG |
| **BdHsf-08** | ACTTCCTGCAGAACCACGTC | TGTTCATCCCCATGGTCAC |
| **BdHsf-09** | GCAAAGCTCGAATCTTGAGG | TTCTGTCCCTCCACCAGAAG |
| **BdHsf-10** | TGATCAGCAGAGCACGAGAG | TCGACTTGTGTTGCTCGTTC |
| **BdHsf-11** | TCCTTGTACCCCACAGAACC | GCCTGATGAAGCTGGAGAAG |
| **BdHsf-12** | TTGTGGATGCGCTATTGAAG | CAAGGCCTTCCAGTGAACTC |
| **BdHsf-13** | GCAACTTCTCCAGCTTCGTC | TCCAAGTCCAATTCGTCTCC |
| **BdHsf-14** | GAGCAACACGTTCCTCGTC | TCTTCTTCCTCCTCGCACAG |
| **BdHsf-15** | GGGAGTTTGCAAATGAGGAA | GTCTTCCAAAGCCTGCATTC |
| **BdHsf-16** | CCAACTTCTCCAGCTTCGTC | CTCGATGGCCTTCTGCTC |
| **BdHsf-17** | GCAACTTCTCCAGCTTCGTC | TCTTGTCACGCTTCAACCTG |
| **BdHsf-18** | GGAGCTCGGACACATGAAG | ATCGCGCTCATCTTCTTCTC |
| **BdHsf-19** | ACGTCCATATGTCCCTCCAA | GTGTCGCTGCATCTGCTTTA |
| **BdHsf-20** | CATTCAACACGCAGCAAGAT | TCTGCTCATCATCTGCATCC |
| **BdHsf-21** | TCCTGACGAAGACGTACCAG | GCTTCTCCCCTTTCCTGAAG |
| **BdHsf-22** | ACGGTTTTCGTAAGGTGGTG | GACGAAGAGCTGTGGTCGTT |
| **BdHsf-23** | GTGATTTGGGCGAAGAGAAC | CGAGGGCATTAGGTCCAGTA |
| **BdHsf-24** | AGTTCGCCAACGAGTGTTTC | GCGAAATCTCCTCCTCCAG |
| **BdcHsp70-1** | GCTCGTCGGAGGATCTACAC | GTCCTGCACTTTCTCGTTGC |
| **BdcHsp70-2** | GTCATCCCTGGACCTGGC | ACGGCGTTCTTGATGGAGTT |
| **BdcHsp70-3** | GGTTCTCTGATGCCCCTGTC | CAGCAATCTCACGCATCTTGATA |
| **BdcHsp70-4** | GCCCTTCAAGGTTGTTGCTG | ACGGCATTCTTCACAGTGGT |
| **BdcHsp70-5** | CTCGACACAAACCAACTCGC | GAGGGCGAATCCTCATCCAT |
| **BdcHsp70-6** | CAGCAACAACAAGGGCGAG | GTCGCCGATGAGCCTCTC |
| **BdcHsp70-7** | CCAGACCAGCATTCTCTTCCC | ACCTTCAGGATGCCATTTGCT |
| **BdcHsp70-8** | AATCCCCAAGGTGCAGAACC | GGAGTGACGTCGAGCAAGAT |
| **BdcHsp70-9** | GCTCTCGCTCAGGATTGAGG | GCGTGAACTTTCCAAGCAGG |
| **BdcHsp70-10** | CGGTCTACTTCAACGGCTCA | TATCCAAGGTACCGCCACCA |
| **BdcHsp70-11** | TGCAGGGTTGGTGCCATAAT | GCTGGGACCTGAGATGACTT |
| **BduHsp70-1** | ACCTGGGGACGACCTACTC | AATCGAACATGCCACAACTGG |
| **BduHsp70-2** | TCGTCGCACCACCTGAAG | GTCGTAAGGCTCCTCGTCC |
| **BdBip1** | GGTTCACCGATAGCGAGAGG | AAGGCTTCCCATCCCTGTTG |
| **BdBip2** | TGTTCCTCGTGGGGTTTCTG | GGGTGATGCGGTTGCCTT |
| **BdBip3** | CGGGGGAGAAGGAACAAGC | GACGGCGTGATGCGGTTA |
| **BdcpHsp70-1** | AGAGAGACGCAATCGACACC | CGGCATCTTTGAGTTCCACC |
| **BdcpHsp70-2** | ACAGGGTGGGGTTTTAGCTG | ACTTGTCTGTCCATCAGCGG |
| **BdmtHsp70-1** | CGGAGATTGCCACCGAGATT | TTGTGATCCTTCCTGCGGAC |
| **BdmtHsp70-2** | TCCCGCAGAGGTCGTGTC | TCCACCCTGCGATCCTGA |
| **BdmtHsp70-3** | ACCAGACGCAGGTTGGAATC | CCTTTGCAGACACCCTGACA |
| **BdHsp110-1** | AGAGGCTCGTGAGGGACTAT | CATCTACGGTTGTCGCACCA |
| **BdHsp110-2** | ACTCTCAGGTTGCACTGTCG | GTGGTCAGTTTGTGAACCGC |
| **BdHsp110-3** | GGTTGGACGAGAAGGAAGCA | ACTCTTCTTCCGTTGGAGGC |
| **BdHsp110-4** | GTGGGCAATGTGAAGGCAAG | GGCTCGACAACTCTCTCCAG |
| **BdHsp110-5** | AGTTCCGGTTACGAGTGAGG | GCTCGACCTTTGTGTCCGT |
| **BdHsp110-6** | GGCGACTCGTCTGCTCTAC | TTGGTGTTGCCGATGTGGAT |
| **BdHsp110-7** | GTTGTTGCGAAGAGTGCGAG | AGTTCTCCAACCGCAACACA |
| **BdHsp110-8** | ACAACGAGACCCTGCTTCTG | TCAGCGATAGGCACATTGGT |
| **BdActin** | GTGTCCTGAAGTGCTGTTCC | TCTCCTTGCTCATGCGATCA |
| **BdWRKY36** | GATGCCACTGCTCCAAGAGA | GCACTTGTAGTATCCCCGTG |
| **BdCBF1** | ACATGACTGTGTCTTCCGCC | ATGTCCCGAGCCATATCCTC |
| **BdCBF2** | CTACGAAGATGACGGCGGAG | TTCTTGTTGGGCTCCCTGAC |
| **BdP5CS1** | CAAGCGCATCGTCATCAAGG | GTACTTGAGCCTCTGCCTCC |
| **BdAPX1** | CCTGAGTGGCGAGAAGGAAG | TACCCGCCAAATCCCAACTC |
