# Supplementary material for: Genome-wide survey of heat shock factors and heat shock protein 70s and their regulatory network under abiotic stresses in Brachypodium distachyon
Source: PLoS One. 2017 Jul 6;12(7):e0180352. doi: 10.1371/journal.pone.0180352 (PMC5500289; doi:10.1371/journal.pone.0180352)
Supplement: S1 File — (DOC) [file pone.0180352.s005.doc]

S1 file. The peptide sequences of Hsf and Hsp70 members in *B. distachyon*, *A. thaliana* and *O. sativa*.

>Bradi1g01130 (BdHsf01)

MEGGVVAAAAAAAAAAATVTTAVSPPPAPPAAASNGGGGAAAAAPPPFLMKTYEMVDDPGTDAVVSWGPGNNSFIVWNTPEFARDLLPKYFKHNNFSSFVRQLNTYGFRKVDPDRWEFANEGFLRGQKHLLKTINRRKPLHGNNQMQVQQQQQQQQQPQLQNAPIPACVEVGKFGMEEEIEMLKRDKNVLMQELVRLRQQQQTTDHQLQTLGKRLQGMEQRQQQMMSFLAKAMQSPGFLAQFVQQNENSRRRIVAANKKRRLPKQDDGLDSESASLDGQIIKYQPMINEAATAMLRKILQQDSSHRYESMGNSDNFLLENSMPTAQAFDSSSSTRNSAVTLAEVPGNSSIPYMATSSGLSAICSSSTPAEIQHPVLDNILSKELPNMSSAPSVPTAMAPGPNDIGIPGFPDLHDIITEDVVGIPGGSFEMPGPECIFPLPEDGDDSIPIDTDEILSSDDTQKLPAIIDSFWEQFLVTSPLSVDNDEVDSGLLDTREAQLDNGWARTDNLANLTEQMGLLSSNHRGKHTENCLSTDDMWEDSSVGPARNMVLLRGYYLLVSKVFAGVRRKSGSMVTCYVCIMASLQLCVVTADSFGCAIWLQLTCGNTAGQLYSQPSVVELA

>Bradi1g05550 (BdHsf02)

MMMNPVKVESRASSVAANGAAPRPMDALADAGPTPFLAKTYDMVDDPTTDAVVSWSATNNSFVVWDPRMFGTVLLPRYFKHNNFSSFVRQLNTYGFRKVDPDRWEFASEGFLRGQRHLLKNIKRRKPPQASPNQQSRGSYLEVGHFGYDGEIDRLKRDKQLLMAEVVKLRQEQQNTRAGLHAMEERLQGTEQKQQQMMSFLARVMHNPEFIHQLVSQSEMRKELEDAISNKRRRRIDQGPEAVDSTDTNSSLEQGSQIMFEPPDPVDAFINGSPSDLESSSVETKGAEVQQGAASGSSPPLKGRPSRELNDDFWEDLLHQGGLTGDEAGDPVVPDDMNFFAQKMNFP

>Bradi1g08891 (BdHsf03)

MYPFSGIVKEEEFDFAGAYYAAEDGGSPSSWAAGAGASELPRPMDGLGEAGPTPFLTKTYDVVSDHSTDTVVSWSVAGNSFVVWDAHAFSRVLLPRYFKHGNFSSFVRQLNTYGFRKVDPDRWEFAAEGFLRGQKELLKTIRRRRPLSSSSSAQQQQQQGAAAGCLEVGQFGHEGEVHRLKRDKGVLISEVVKLRQEQQATRAQMQAMEARIVATEQKQQQMTVFLARAMKSPGFLQMLIDRQQGQGPQGHLGPGQAQLRRELEDALSKKRRRPIDYLLPRNGDTSAASYSAAAAAAAARDYVPGLADGHARAEDGRSGGGEDTESFWMELLSLGLEEKQGGGAGGGSGSGEGGGAETDNEVDDEVDVLVQSLYHLSPGGAHNAE

>Bradi1g19900 (BdHsf04)

MAFLVERCGEMVVSMESSAHGGGGGVGGGKAVPAPFLSKTYELVDDPCTDHIVSWGEDEATFVVWRPPEFARDLLPNYFKHNNFSSFVRQLNTYGFRKIVADRWEFANEFFRKGAKHLLAEIHRRKSSQPPPPLPHHHHAYHHLHHHHHHLGTAFSPPPPLAAHHHHPMSSYHFQEDPPAPAIGSNNNGGQGSGGEFLAALSEDNRELRRRNSVLLSELAHMKKLYNDIIYFLQNHVAPVSLPTPQTPSNLISGIVHGGAGAAANTNSSCRLMELDEEGEDETASVKLFGVALKRTKRARRPEERFCDLGSEA

>Bradi1g37720 (BdHsf05)

MEYSAILTDVKQEEPELVVLDDAGDEDDGCLLVPPTPMDLSASAAVAPFLAKTFDMVEDPATDSVVSWGAARNSFVVWDPHAFAARLLPLHFKHANFSSFLRQLNTYGFRKVNPDRWEFANAGFLGGQRHLLAGIRRRRGADRRPACPSSSSAAEVGGVVEGELERLRRDREALARELARLKRQQEESRAALLDMERRVQGTERRQEQCKAFLARAVRNPNFLDNLASRNGIGIAPVEDGCKKKRKMLDAGMTPTPADGITFEEMALAAGVDVIATSQSAVDGGGSGVTTDMVWYELLGEEQTEIDVELDELVAAAAAADAEPWEEIGDEEVQE LVHQIDCFAGSPSS

>Bradi1g38140 (BdHsf06)

MSVGGGGAAAAPFVWKTYRMVEDPGTDGVIGWGPANNSFVVADPFVFSQTLLPTHFKHNNFSSFVRQLNTYVSQLLHFLLKSEGFRKVDPDRWEFAHGSFLRGQTHLLRNIVRGGGGGSKRKDAAAADATDQDMTMVATEVVRLKKEQRTIDDRVAAMWRRVQETERRPKQMLAFLLTVVGDRDTLLRLVSGNDGAGDEEPVEGGEKRARLRLDGPEAADFAGFYGVGGDAFVNQLAVDVAAGAGSSVGGAAGSSFGFGGDSGY

>Bradi1g55630 (BdHsf07)

MDPLLSLVKEEQEGHGGGGGSPPAAVAAEDGPSTVAAAAVPRPMEGLHDVGPPPFLTKTYDMVDDQNSNHVVSWSPTNNSFVVWDPHAFATSLLPRHFKHSNFSSFVRQLNTYGFRKVDPDRWEFANEGFLRGQRHLLKNIRRRKPPAHPASNQQSFGSSYLEVGHFGNDAEIDRLKRDKELLMAQVVKLRQEQQDTKARLKAMEDRLHGNEQKQQQMVTFLARVLRNPEFLKQLIAKNEMRKQLHDTISKKRRRRIDQGTEADHMGASSSLQQESPILFDPHVSMELLADGSVQLLADDIPPDLEGSGGFVADGIPPDMESSVEHVADGILPGLEGSVELLADGIPPDLEGSLDLLADGIPLNLEGSMELLADGIPSGLEGPGIKTNGVMEPHGFDLGICGMQQNRAQGVLKDNFWDDLLNEELGDEDDNPVIMEDMNLLSEKMGYLVSDTPTPTK

>Bradi1g61620 (BdHsf08)

MAFLVERCSGEMVVSSSSMEHMAAAAKPVPAPFLTKTYQLVDDPCTDHVVSWGEDEATFVVWRPPEFARDLLPNYFKHNNFSSFVRQLNTYGFRKIVADRWEFANEFFRKGAKHLLSEIHRRKSSCSSSQPLPPPPPQPYLSLFSPPPHHHPQLAQGAYHRFQEEEEYSSSPADSGGDLLATLSEDNRQLRRRNSLLLSELAHMRKLYNDIIYFLQNHVEPVPRQQPASCYTRLVELDHAGGPVVVPQAQAQARQRGDDDAAVKLFGVRLDDSKKRRVLLVEEGDHGDHGDEHLEISNEA

>Bradi1g69407 (BdHsf09)

MGSKKASPPPSGAASASRAAGLNEASSDVGASTGNGTAPVGAVPKPPDVPPFLTKVYDMVSDPATDKVISWTQAGSSFVISDSHAFERDLLRRHFKHSNFSSFIRQLNTYGFRKVDPDRWEWANEGFLRGQKHLLKTIKRKKRSPQEAGSELEQAPVKTPPGTENIEIGKYGGLVKEVETLKRDKALLMQQLVDLRHYQQSSNLEVQNLVQRLQVMEQNQQQMMALLAIVVQNPSFLNQLVQQQQRRSNWWNADGNKKRRFPALEQGPVTEQETSGGGTEIIQYLPPVPETSGQGIPDEAFCSATAQTTSSPPLDMPMDIDTKTTSDNLDNVGSFGDFLTDTPADWDIELFFDDDGEPLIAPLENNGQVDPPLSVQDYDFPQSEQDCQMEAQA

>Bradi1g74350 (BdHsf10)

MMEHAVLPVPGRVKEEWSPEEEEEAPRPMEGLHETGPPPFLTKTFDLVADPATDGVVSWGRAGNSFVVWDPHVFAAVLLPRSFKHNNFSSFVRQLNTYGFRKIDPDRWEFANEGFIRGQRQLLKMIKRRKPLPYLPSSQQQVLGSCLEVGQFGMDEEIEILKRDKNALLAEVVKLRHDQQSTRADMRAMEERLHLAEQKQLQMMGFLARAMQNPDLFLQLIEQQDKWKDDASLKRRRSIDMAPFLSPREATQNEQHKSTILSEPREFAVPNQPGFSELENLALSIQGIGKGTKDDKGCQNQVSGEVELTDDFWEELLSEGMKDESGMPEPETRRPRYVDA

>Bradi2g18980 (BdHsf11)

MHGSELNRCFRSGPGRASGVSPGSVSRSTKPTPAFIHCISLWVDPVSQTSHATTSPGDARTKPRKAPSPRRGGRCRRSSIKRPPATGGAASLYPTEPSRFVRFRGNMESCQSSQGGGGGPPPFLIKTYEMVEDPATNRVVSWGPGGASFVVWDPPEFSRDMLPKYFKHNNFSSFIRQLNTYGFRKIDPERWEFGNEDFVRGHMHLLKNIHRRKPVHSHSLQNQANGPLAEAERRDLEDEISRLKHEKSVLLADLQRQAQQQCGINWHMQSLEDRLVVMEQRQENVVAYLGDILQRRRGTVSGSVLLETDHFSKKRRVPRIDFFAQEPAVEEQRVPYLPAMVAETPGVLPPVSGANAEPFEKMELALVSLEKLFQRAGNYAPYEDVYNAAAAPSSALALGNLQAAPMEGSINLQPSAELAEPPGYSQSPVLPSAYIHEDMGKTITGVDMNSEASTSGTSQDETDCRNQSFP

>Bradi2g41530 (BdHsf12)

MASSSRCALFVGSKRTRTSGGAGGAGGEEAMAWGSAVVKREVKPEEQEEEEAREARAAWPRPIEGLGEPGPAPFVGKTYEMVADAATDAVVSWAGRGSSFVVWDPLALAAAVLPRFFKHANFASFVRQLNTYGFRKVNQERWEFANEDFLAGQKHLLKNIRRRRASRHHMKSQLRNGSSVCYRQPESLSEVENLKRDHTALRAEAVKLKQQYSICKSQLLAMEQRVLSNERKQQQIITFFVKSLSNPVFLQQIWLNYGNKKELGSTVKRQRLMENEEQHVVDALLKKGTDAAFEKEVSISAGSSDCGTVENDEPMRKWNDQNIGDMCDDVWEELDAIPGIEMKQEGKADIGFDVEEFTGRPCSWVDDCPYLVEPLQFVEH

>Bradi2g44050 (BdHsf13)

MDGLHTELALGLIGQLQTAPFVAKTYQMVCEPRTDALIRWGGENNSFVVADVAGFSQLLLPCFFKHGNFSSFVRQLNTYGFRKVHPDRWEFAHESFLRGQTHLLPRIVRRKKRGEGGGSASCSSATIDSGHEPQHVASASSTGDELDLDDDEEEEGSEAVLLEEVQRLRREQTAIGEQLARMSRRLQATERRPDQLMSFLTRLADEDSSVQLLEQAAAEKKRQRMQQMQMQFTPSRDFAAPSSPSPIALRPPPPRPPAVIDDAAMVDGVWRWAEEQKPARRSITTSEQPAASSGAQQVPEFEGGGGSGVGVGLTDDGGAAAETPFPFCLLGQCFF

>Bradi2g48990 (BdHsf14)

MGSECKGHHQVQDDEAGGPGAIAPFVAKTFHMVSDPATDGVVRWGGASNTFLVLDPAAFSDLLLPSYFKHRNFASFVRQLNTYGFRKVDPDSWEFAHESFLRGQAKLLPLIVRKKKKAGARGELCEEEEEVRGTIRAVQRLRDERKGMEEELQAMDRRLRAAENRPGQMMAFLGKLADDPGVVLRAMVAKKEELSAAAGGGKDSSSPQKRRRIIGAEAGRGGAVSAADAAESRAVPFPFSALGQVFY

>Bradi2g49860 (BdHsf15)

MDPGGGAASLPPFLTKTYEMVDEPATDAVVSWTPSGTSFVVASQADFCRDLLPKYFKHNNFSSFVRQLNTYGFRKVDPEQWEFANEEFIRGQRHRLKNIHRRKPIFSHSSHTQGAGPLVDSERRDYEEEIERLKCDNAALTSELEKNAEKKIDMEKRMQALEDKLFAVEDQQTNLICYVRDIVKEPGFLSSFVQQSDHSRKKRRLPKPISFHEDTSTQGNQIMHRDLTNSPAHELSRESFDKMESSLNSLENFLREATEAFGNGISYDCDIPGPSAVVLTELHLSGESDPHAPSPPSMMHTSSAGVGDSHSSRGIAESTSCTESPPLPQTHSRADSRAKVSEIDVNLEPAVTETGPSRDQPGQDPPATAAAGANDGFWQQFLTEQPGSSVAHQEAQSERRDREADQAKNGDRANCWWGKKNIEQMTEKLGHLTSAEKT

>Bradi3g08870 (BdHsf16)

MMTGGGGGGEAAGVAPFVAKTYGMVEDPATNGVIAWGSGSNSFVVIDPFVFSQTLLPTHFKHSNFSSFVRQLNTYGFRKVDPDKWEFAHVSFLRGQTHLLRQIVRRSSSSGKRKDDGGCAGASGADDHDDDSTTMVAMEVMRLKQEQKAIEDRVAAMWRRVQETERRPKQMLAFLLKVVGDPQVLRRLVANTSSSNSNSSSGGSDRSEVAFHGGGGPAEGAEVKRPRLLLERDHQHGGKMSSSPAADGVGIGIGSGVGVRRDGFYGVSEETFAPEHSVDFTGFYTGGDGFGDGQVDAGGGGGDLPYAFPVDSGY

>Bradi3g26920 (BdHsf17)

MDPVAGGIVKEELLEQDGGVGAAGLPRPMEGLHEAGPPPFLTKTYDLVGDPSTDQVVSWSPAGNSFVVWDPHVFADVLLPRLFKHSNFSSFVRQLNTYGFRKVDPDRWEFANEGFLRGQRHLLKMIKRRKPPSNLPPSQQQALASCLEVGEFGHEEEIDRLKRDKNILITEVVKLRQEQQTTKGHVQAMEERLRTAEQKQAQMMGFLARAMRNPRFFQQLVQQQDKRKELEDAISKKRRRPVDHVPFYGPGIASQNEQLDSQFLFDSGVLGELSEPGMPGLENLAHNIQELGKSTADEENRDQASGLGELNNEFWAELFDDDYRDGSGQSELEGRRPEDIDELAQQLGYLSSTSPR

>Bradi3g42130 (BdHsf18)

MAAASSSAGQRGAAPTPFLAKTYQLVDDPAVDDVISWGEGGATFVVWRPAEFARDILPSCFKHNNFSSFVRQLNTYGFRKVVPDRWEFANDLFRRGEKRLLCEIHRRKVTPPTSAVTVSPAAAAIPMALPVATATTSPVLSAEEQVLSSSSSSERELPSAFPPPSCSGSGSGVGGDLGDENQRLRRENARLARELGHMKKLCNNIFALMSKYASAPLDAPAPASAGGGNCSGESPLPPPPPPTPPSLELLLSSSSPGPPADADEEKKMSAMLFGVCIGRKRMRNDDGHGVGGAAEVKPEPMDGRPSPPPPPMEPQGWPVYRPRPVYRQHLLHACCSDGHAGSNSK

>Bradi3g43710 (BdHsf19)

MEASSGAGARGGGGGGGPAPFLLKTYEMVDDPSTDAVVSWSDASDASFVVWNSPEFAARLLPTYFKHSNFSSFIRQLNTYGFRKIEPERWEFANEYFVKGQKHLLKNIYRRKPIHSHSHQPGALPDNERALFDDEIDRLAREKAALQADLWKFKQQQSGTMFQIEDLEQRVLNMEQRQGKMIAFLQQASKNPQFVNKLVMMAESSSIFTDAFHKKRRLAGLDYATETAEATSFYDEHSTTSKQEMGNLLNQHFSDKLKLGLCPAIAESNLITLSTQSSHEDNGSPHGNHPVCDRMGMECLPLVPQMMELSDTGTSICPSKNSCFTPSVNDEGLLPCHLSLTLASCSMDVDRSQACNADGSTTIDEGRDDPAEATTATTDDNQKTPVDSDKADAATQRRGDARIATEAPAAPPAVVNDKFWEQFLTERPGCSETEEASSGLSRDPSMEDKGQMEGNMKDGSEDMEQLKL

>Bradi3g44700 (BdHsf20)

MDHANNNNPATSSMDAALLLLEPKLEMEPPLHLPPPPTANHHHHLIPPPPLAVPSEPPRPLEALLQGPQLPPFLSKTYDLVSEPLLDGVISWGHAGNSFVVWDPSTFARDVLPHNFKHNNFSSFVRQLNTYGFRKVHADRWEFAHEGFLRNNKHLLKTIVRRRSSPTQQSSLQSASSIFRKAQPCSSGEPTVDPELHILKREKKALLQEVARLKQEHRQTIAHMSTLNQRLESAEDRQKQVVSFLAKLLRNPAFLRQLTMLREHKEIESSRVKRKFLKHAPHGSTDSDLQSFLLEDTDLSDGMLPGNFGLDGVEAPGDVGALVQAFNTQQDGLDFGTGAELLGTASGAAHCQDPTIGRSKGKNVLCPGLDGATSSEAECLVSLPDNMGMIPGTVLETAGKLMDADDEQIWGVDPYLQSSCCDTRQQAYGSGASDPYLMEIANKPEKFWELDFEDLDDGDLHLDKCVIGDPALQQQRGNMKP

>Bradi4g32050 (BdHsf21)

MERCGSWSDCDAAAAQAAAQKAVPAPFLTKTYQLVDDPATDHIVSWGDDRVSTFVVWRPPEFARDILPNYFKHNNFSSFVRQLNTYGFRKVVPERWEFANEFFRKGEKQLLCEIHRRKTSGSTTSPSPPPFFAPPHFPLFHHPGVSVAQHHHQQFVGDDGVIAAAAHGMGVLPFMQPHWQQREQSQSAAPVATRLLALGPSSEGGSRAANNAAAAGALMAENERLRRSNAALLQELSHMRKLYNDIIYFVQNHVRPVAPSPAAATFLQGLGLQAPARKKQPATAAAGNGLNTSGGSTTSSSSLTIADELSPPPPHHLAAEKSGGEGAGSSSAARSSAAAPTKLFGVHLSATPFGAGSKRPPSPEAELPSTPPATKPRLLLECDDLSLSVAQPCAATSSPARASS

>Bradi4g32130 (BdHsf22)

MAGAAAAQQKRGGGGRGSAGSGGGGPAPFLTKTHQMVEERGTDEVISWGEEGRSFVVWKPVELARDLLPLHFKHCNFSSFVRQLNTYGFRKVVPDRWEFANENFRRGEQSLLSGIRRRKAAATTTTPQSSKSCGSGGGVNVAFPPPLPHTLPPASASTSGNDHSSSSGASSPSRHPDLASDNQRLRKANHTLALELALARRRCEELLGFLSRFLDVRQLDLRLLMHDEDMMQGAVARERCQERQAGCEEKTVKLFGVLLKDAPARKRARCDEAAASERASIKMIRMGEPWVGVPVPPAGPAR

>Bradi4g35780 (BdHsf23)

MAEQQSAAIAAAAEGAGGEPPVPVPAALVAPADAAGQRSLPTPFLTKTYQLVEDPAVDDVISWNEDGSTFVVWRPAEFARDLLPKYFKHNNFSSFVRQLNTYGFRKIVPDRWEFANDCFRRGEKRLLCDIHRRKVVQSSAGLAAAAAAAAAGAVTVATAAIPMALPVTRSGSPEPHSSEEQVLSSNSGSAEERLPGPSGSGSGLGGGAGGGSSSGDLGEENDRLRRDNTRLTRELGQMKKLCNNIVLLMSKYAATQQPDGPASLSSVVNCSGESALAPPPPLPTAILDLMPSCSALATAAGLAVDGEPDTSARLFGVSIGQKRSRDSDDGGGREEDPRDDGGEGADVKPEQADRRPDSQERSPDGPDQHSWPIYRPKPVYRACNGPDGAGSDQDRSNSR

>Bradi5g18680 (BdHsf24)

MASPAAGTAPFLTKTYAIVDDPETDDIISWNDSGTTFVVWRRSDFERDLLPKNFKHSNFASFVRQLNTYGFKKVGVDRWEFANECFRKGEKHLLGGIQRRKGSGGAGAPASAVIPTAIALPISPTATSSGGDPPVSSSSPPRPGSGSAVSGAVAELEEEISRLRRENARLSRELARARRAFDDVRRVVTRYDHGGEEEDERPGAAGGGGKPMLFGVAIGSKRSREVDGDEEDGAEEDGGDEDEEEEDDDDERHAARRDKARRTELSDLNVLALSVRAAAAARAPDVGSRGTKNHSVSLPLGSNRATL

>LOC_Os01g39020.1 (OsHsf01)

MLKPQTPRARRAAHPNSHMASSSSSSSLCRLLIPRPTTRRFSGGGGEGGMAAAAPVKREVKPEAGEGWGGGDLGVVPPPPRPMEGLGEAGPAPFVAKTYEMVADAATDAVVSWGPGGSGASFVVWDPHALAAGVLPRFFKHANFSSFVRQLNTYGFRKVTPDRWEFANEAFLAGQKHLLKNIKRRRVSKPLVDSQLRNKASVVFGQPEAPGEVVSLKRDRAALRAEVIMLKQQYNACKSQLIAMEEMVRNIERRQQQTIGFFAKVLTNPAFVQQVLLNYVNKNGLRGAAKRQRLMENEEQHADSPLNKGMEAASVMEADVSPGSTGCGTVGKVETTPMCNFQNIENMCDDVWEELDALPETGMEQEEKAGIGSFDVEEFVGRPCGWVDDCPYLVEPMQFVEH

>LOC_Os01g43590.1 (OsHsf02)

MDGLHTELALGLIGCCGGDGQQQTAPFVAKTYQMVCDPRTDALVRWGRDNNSFVVVDPAAFSQLLLPCFFKHGNFSSFVRQLNTYVSIIQSPAPGFRKVHPDRWEFAHESFLRGQTHLLPRIVRRKKRGEGGGGGGGASCSFGGGAGEHQVAAAAASVGMSGEEEDAAEDVLAKEAALFEEVQRLRHEQTAIGEELARMSQRLQATERRPDQLMSFLAKLADDPNAVTGHLLEQAAERKRRRQHLPSHEPTVCPLPPAPPPQPPQPLLALAGAAAMDGTYWWTTEHHHHHHHQMKPMTVLPSLEPPTASCGVHQVPELGGGGVMGLTTDGEAKV EPPFPFCLLGQAFF

>LOC_Os01g53220.1 (OsHsf03)

MMGGECKVHQLQAAGDGGPGAVAPFVAKTFHMVSDPSTNAVVRWGGAGNTFLVLDPAAFSDFLLPSYFKHRNFASFVRQLNTYGFRKVDPDRWEFAHESFLRGQAQLLPRIVRKKKKGGAAPGCRELCEEGEEVRGTIEAVQRLREEQRGMEEELQAMDQRLRAAESRPGQMMAFLAKLADEPGVVLRAMLAKKEELAAAGNNGSDPCKRRRIGADTGRGGVATGGDAAEMAQSRGTVPFPFSVLGQVFY

>LOC_Os01g54550.1 (OsHsf04)

MEGGGGGGSLPPFLSKTYEMVDDPSTDAVVGWTPAGTSFVVANQPEFCRDLLPKYFKHNNFSSFVRQLNTYGFRKVDPEQWEFANEDFIKGQRHRLKNIHRRKPIFSHSSHSQGAGPLTDNERKDYEEEIERLKSDNAALSSELQNNTLKKLNMEKRMQALEEKLFVVEDQQRSLISYVREIVKAPGFLSSFVQQQDHHRKKRRLPIPISFHEDANTQENQIMPCDLTNSPAQTFYRESFDKMESSLNSLENFLREASEEFGNDISYDDGVPGPSSTVVLTELHSPGESDPRVSSPPTRMRTSSAGAGDSHSSRDVAESTSCAESPPIPQMHSRVDTRAKVSEIDVNSEPAVTETGPSRDQPAEEPPAVTPGANDGFWQQFLTEQPGSSDAHQEAQSERRDGGNKVDEMKSGDRQHLWWGKRNVEQITEKLGLLTSTEKT

>LOC_Os02g13800.1 (OsHsf05)

MTTTTAEGGGGVAPFVAKTYRMVDDPATDGVIAWGRDSNSFVVADPFAFSQTLLPAHFKHSNFSSFVRQLNTYGFRKVDPDRWEFAHVSFLRGQTHLLRRIVRRSSGGGGAKRKEEAGGCGGGGEAAAGDVDEESAVVALEVARLRREQREIEGRVAAMWRRVQETERRPKQMLAFLVKVVGDPQVLRRLVDRDNTNAAASNADDSAVHHQVKRPRLLLDSSSTTTTHGDRHLVTAAADGFYAGGCGPEAAAAAAFVPDDAVDFTGLYTGGDGFGNAVVDAGVDYPPAYAFPVVDSGY

>LOC_Os02g29340.1 (OsHsf06)

MEVAAGARGGGAGGGGGGPAPFLLKTYEMVDDPSTDAVVSWSDASDASFVVWNHPEFAARLLPAYFKHSNFSSFIRQLNTYGFRKIDPERWEFANEYFIKGQKHLLKNIHRRKPIHSHSHPPGALPDNERAIFEDEIERLSREKSNLQADLWKSKQQQSGTMNQIEDLERRVLGMEQRQTKMIAFLQQASKNPQFVNKLVKMAEASSIFTDAFNKKRRLPGLDYSIENTETTSFYDDHSSTSKQETGNLLNQHFSDKLRLGLCPAMTESNIITLSTQSSNEDNRSPHGKHPECDMMGRECLPLVPQMMELSDTGTSICPSKSSCFAPPISDEGLLTCHLSLTLASCSMDVDKSQGLNANGTTIDNPTEAATATMEKDDTIDRSFDDNQKKSADSRTADATTPRADARVASEAPAAPAAVVNDKFWEQFLTERPGCSETEEASSGLRTDTSREQMENRQAYDHSRNDREDVEQLKL

>LOC_Os02g32590.1 (OsHsf07)

MDHNTDPPPTTMVDAAAALLLEPKLEGYDDDGGGEPLQPAPFVSPLDQLMQPPRPLEALLQGPQLPPFLSKTYDLVCEPELDGVISWGHAGNSFVVWDPSAFARDVLPHHFKHNNFSSFVRQLNTYGFRKVHADRWEFAHEDFLRHSKHLLKKIVRRRSSPTQQSGLQPGSSGESGLDPELNTLRREKSALLQEVTRLKQEHLQTIEQMSTLNQRLESAEDRQKQMVSFLAKLLQNPTFLRQLKMHRQQKEIDSTRVKRKFLKHVPHGNIDSGESSSQHTGESNLDFSPTSLDLPATHSDILDLQNFLLEDGDLNLAMLPENIGLDGIEAPDDIGALVQGFDTQEELELGSGVELLEIPPASGPRGQDPTIGRSKGKNVLSPGLDATSSEADCLGSFSDNMGMLSDSMLQTAGKLMDADDDERIWGVDASSALQSSCSGTSQQAYGSLVSDPYLMEMANKPEKFWELDFQALDDGDLQLDKCVIDDPALQQQRGNMNS

>LOC_Os03g06630.1 (OsHsf08)

MEKMMPGMVKEEWPPSSPEEGEAPRPMEGLHEVGPPPFLTKTFDLVADPATDGVVSWGRAGSSFVVWDPHVFAAVFLPRFFKHNNFSSFVRQLNTYFLVRTNYLNKRSHFYSLRFQGFRKIDPDRWEFANDGFLRGQRHLLKMIKRRRPLSYLPGSQQALGTCLEVGQFGLDEEIDRLKRDKNILLAEVVKLRHKQQSTKANMRAMEERLQHAEQKQVQMMGFLARAMQNPDFFHQLIHQQDKMKGLEDTFSKKRTRSIDIVPFLNPGEVSQGDQLESTLLFDPRPFAELNDEPAKSELENLALNIQGLGKGKQDVNRTRNQPRNQASNETELTDDFWEELLNEGARDDAGIPGMERRRPRYVDALAQKLGYLSNSSQK

>LOC_Os03g12370.1 (OsHsf09)

MGSKKRSPQHPAAAAPPPAVGGGGGGEVSGDGGASTANGPVVPKPSEVAPFLTKVYDMVSDPATDNVISWAEGGGSFVIWDSHAFERDLHRHFKHSNFTSFIRQLNTYGFRKVHPDRWEWANEGFIMGQKHLLKTIKRRKKSSQESPSEIQKAPVKTAPGTENIEIGKYGGLEKEVETLKRDKALLMQQLVDLRHYQQTSNLEVQNLIERLQVMEQNQQQMMALLAIVVQNPSFLNQLVQQQQQQRRSNWWSPDGSKKRRFHALEQGPVTDQETSGRGAHIVEYLPPVPETSGQVNPVEGAICSANSQPVPSPAVATPMDMQTSNVADTLGSSEEPFADNSTLHEWDDNDMQLLFDDNLDPILPPFENDGQMGPPLSVQDYDFPQLEQDCLMEAQYNSNNPQYADVITEA

>LOC_Os03g25120.1 (OsHsf10)

MAFLVERCGGEMVVSMERSHGRSTTTAAAVTAAPAPFLSKTYQLVDDPSTDDVVSWGEDEATFVVWRPPEFARDLLPNYFKHNNFSSFVRQLNTYGFRKIVADRWEFANEFFRKGAKHLLSEIHRRKSSSCSQPQPPPPFPMHQHYPLSLFSPPTTPRSPPVGAAAAAAYHFQEEYCSSPADYAGGGGDLLAALSEDNRQLRRRNSLLLSELAHMRKLYNDIIYFLQNHVEPVAPPPLAAATSCRLVELGPSTTERRRCAASPSGDNDDDAAVRLFGVRLDDDHGKKRRVQLVQEDEGDEQGSEG

>LOC_Os03g53340.1 (OsHsf11)

MNPLRVIVKEEELDFAAAAAAAAAGEGSPSSWAVGVMDLPRPMEGLGEAGPPPFLCKTYEVVDDPGTDTVISWGFAGNSFVVWDANAFAAVLLPRYFKHSNFSSFVRQLNTYGFRKVDPDRWEFANEGFLRGKKELLKTIKRRRPPPSSPPSSSSSSSSSQHQQQPAAACLEVGQFGRDGVVNRLQRDKSVLIAEVVKLRQEQQTTRAQMQAMEERISAAEQKQQQMTVFLARAMKNPGFLQMLVDRQAGQHGARNRVLEDALSKKRRRPIEYLLTRNGETCAAGESAAMLAADGVAEPDGDTTPRGDGGGGGGGDTESFWMQLLSLGLEEKQREDGVAGGVQESNSGGADVDNDEEDDDDDVDVLVQSIYHLSPK

>LOC_Os03g58160.1 (OsHsf12)

MNYRVVNPVKVESGPSTGVANGQPPRPMDGLADGGPPPFLTKTYDMVDDPTTDAVVSWSATNNSFVVWDPHLFGNVLLPRYFKHNNFSSFVRQLNTYGFRKVDPDKWEFANEGFLRGQKHLLKSIKRRKPPNSSPSQQSLGSFLEVGHFGYEGEIDQLKRDKHLLMAEVVKLRQEQQNTKSDLQAMEQKLQGTEQKQQHMMAFLSRVMHNPEFIRQLFSQSEMRKELEEFVSKKRRRRIDQGPELDSMGTGSSPEQVSQVMFEPHDPVDSLFNGVPSDLESSSVEANGGKAQQDVASSSSEHGKIKPSNGELNEDFWEDLLHEGGLDEDTRNPAIDDMNLLSQKMGYLNSSSTKSPQ

>LOC_Os03g63750.1 (OsHsf13)

MEAAVAAAAAAAGAVTTAVAPPPGAAVSNGVATAPPPFLMKTYEMVDDPATDAVVSWGPGNNSFVVWNTPEFARDLLPKYFKHSNFSSFVRQLNTYGFRKVDPDRWEFANEGFLRGQKHLLKTINRRKPTHGNNQVQQPQLPAAPVPACVEVGKFGMEEEIEMLKRDKNVLMQELVRLRQQQQTTDHQLQTLGKRLQGMEQRQQQMMSFLAKAMHSPGFLAQFVQQNENSRRRIVASNKKRRLPKQDGSLDSESASLDGQIVKYQPMINEAAKAMLRKILKLDSSHRFESMGNSDNFLLENYMPNGQGLDSSSSTRNSGVTLAEVPANSGLPYVATSSGLSAICSTSTPQIQCPVVLDNGIPKEVPNMSAVPSVPKAVAPGPTDINILEFPDLQDIVAEENVDIPGGGFEMPGPEGVFSLPEEGDDSVPIETDEILYNDDTQKLPAIIDSFWEQFLVASPLSVDNDEVDSGVLDQKETQQGNGWTKAENMANLTEQMGLLSSHHTG

>LOC_Os04g48030.1 (OsHsf14)

MASPAAGTPPFLTKTYAMVEDPSTDETISWNDSGTAFVVWRPAEFARDLLPKHFKHSNFSSFVRQLNTYGFKKVVADRWEFANDCFRRGEKHLLGGIQRRKGSGTGGAGAAPAGGIPTAIPISSPPTSSGGEPAVSSSPPRGAAGIAAGVSGAVAELEEENARLRRENARLARELARARRVCDGVRRLVSRYDHDHGGGEEEAGEGDVKPMLFGVAIGGKRSREENGEDEEEEEEEGADEDGEDDEVEEDDEERERHAARRVPVREGKVRRTTELSDLDVLALSVRAAAAARPGGASRDRKSSVS

>LOC_Os05g45410.1 (OsHsf15)

MESSNLGGGGGGGGGGGPPPFLIKTYEMVEDAATNHVVSWGPGGASFVVWNPLDFSRDLLPKYFKHNNFSSFIRQLNTYGFRKIDPERWEFANEDFIRGHTHLLKNIHRRKPVHSHSLQNQINGPLAESERRELEEEINRLKYEKSILVADLQRQNQQQYVINWQMQAMEGRLVAMEQRQKNIVASLCEMLQRRGGAVSSSLLESDHFSKKRRVPKMDLFVDDCAAGEEQKVFQFQGIGTDAPAMPPVLPVTNGEAFDRVELSLVSLEKLFQRANDACTAAEEMYSHGHGGTEPSTAICPEEMNTAPMETGIDLQLPASLHPSSPNTGNAHLHLSTELTESPGFVQSPELPMAEIREDIHVTRYPTQADVNSEIASSTDTSQDGTSETEASHGPTNDVFWERFLTETPRSCLDESERQESPKDDVKAELGCNGFHHREKVDQITEQMGHLASAEQTLHT

>LOC_Os06g35960.1 (OsHsf16)

MAAAAGGGAAPFVWKTYRMVEDPGTDGVIGWGKGNNSFVVADPFVFSQTLLPAHFKHNNFSSFVRQLNTYGFRKVDPDRWEFAHASFLRGQTHLLRNIVRRGSAAAGGGGGGGGGKRRDASADGGGGGGDEDMTMVATEVVRLKQEQRTIDDRVAAMWRRVQETERRPKQMLAFLLKVVGDRDKLHRLVGGGGNGNGAATAAAADNGFADAARAGCGEKRARLLLDGDNTGAFGPDAVDFAGFYTGADMFPDVAVDAAAAAAGGSAGCSFAFGVDSGY

>LOC_Os06g36930.1 (OsHsf17)

MDYSTVKQEEVEVVVLDGEEEAAAAAAPVPLPAAMGVGAAVAPFLVKTFEMVEDPATDAVVSWGGAARNSFVVWDPHAFAAGLLPLHFKHANFSSFLRQLNTYGFRKVSADRWEFANEDFLGGQRHLLANIRRRRRGAGTGSTTPRAVNCGGGGGEGEVERLRRDKEALARELARLRRQQQEARAQLLDMERRVRGTERRQEQCTEFLARALRSPDVLDNIARRHAAAVERKKRRMLAAAADDDGLTFEALALAAAADTSHSTGGAVTTDMIWYELLGEEQAEIDIEVDQLVASASAAADTASEAEPWEEMGEEEVQELVQQIDCLASPSS

>LOC_Os07g08140.1 (OsHsf18)

MDDPMLNAVKEEESHGDGGGLEVVAGEDGAAAVAAGVAPRPMEGLHDAGPPPFLTKTYDMVDDAGTDAAVSWSATSNSFVVWDPHAFATVLLPRFFKHNNFSSFVRQLNTYGFRKVDPDRWEFANENFLRGQRHLLKNIKRRKPPSHTASNQQSLGPYLEVGHFGYDAEIDRLKRDKQLLMAEVVKLRQEQQNTKANLKAMEDRLQGTEQRQQQMMAFLARVMKNPEFLKQLMSQNEMRKELQDAISKKRRRRIDQGPEVDDVGTSSSIEQESPALFDPQESVEFLIDGIPSDLENSAMDAGGLVEPQDFDVGASEQQQIGPQGELNDNFWEELLNEGLVGEENDNPVVEDDMNVLSEKMGYLNSNGPTAGE

>LOC_Os07g44690.1 (OsHsf19)

MAFLVERCGEMVVSMEMGPHGGGGAAAGKPVPAPFLTKTYQLVDDPCTDHIVSWGEDDTTFVVWRPPEFARDLLPNYFKHNNFSSFVRQLNTYGFRKIVADRWEFANEFFRKGAKHLLAEIHRRKSSQPPPPPMPHQPYHHHHHLNPFSLPPPPPAYHHHHLIQEEPATTAHCTVAGDGGEGGDFLAALSEDNRQLRRRNSLLLSELAHMKKLYNDIIYFLQNHVAPVTTTTTTPSSTAMAAAQHHLPAAASCRLMELDSPDHSPPPPPPKTPATDGGDTVKLFGVSLHGRKKRAHRDDDDGVHDQGSEV

>LOC_Os08g36700.1 (OsHsf20

MEWEEESEAARQKAAAASASVVPAPFLTKTYQLVDDPATDHVVSWEDDDGGESASSFVVWRPPEFARDILPNYFKHSNFSSFVRQLNTYGFRKVVPERWEFANEFFRKGEKQLLCEIHRRKSAAATWPPFPPPPPPFFAPRHFAAGAFFRHGDGMLHGRLGALVTTTERRHWFESAALPVAPSSRLLSQLGPVIAPARRAAATPEEEALMQENHRLLRGNAALVQELAHMRKLYSDIIYFVQNHVRPVAPSPAAAAALHGLGVLRPPPAGGKAPASEVRGASGRSATSSSSLTVAEDQPTLLALRLPRTTEKIINEVSGGNGGGSTKLFGVHLSSADEQTSSGASRKRSPPQEQPPTSPAPKRTLVVEHSELRLSIVSPP

>LOC_Os08g43334.1 (OsHsf21)

MKGSRLAVKESCLPMTMPMPETFAQYSNPLRSTRAYGQLCRVPGRRRHACVDGWGQDRARTRGDDGQRRYAQCRGGWGDIWRWGGYITCGSPRETGMVNGAPSPPPPSPMVMSFGPLDSPWVKQPDTTVYPGQICAAAGGGGGMADQTAAAVVVGGGAAATMGEPSPPPPAPAAEAAGVGVGQQQRTVPTPFLTKTYQLVDDPAVDDVISWNDDGSTFVVWRPAEFARDLLPKYFKHNNFSSFVRQLNTYGFRKIVPDRWEFANDCFRRGERRLLCEIHRRKVTPPAPAATTAAVAAAIPMALPVTTTRDGSPVLSGEEQVISSSSSPEPPLVLPQAPSGSGSGGVASGDVGDENERLRRENAQLARELSQMRKLCNNILLLMSKYASTQQLDAANASSAAGNNNNNNCSGESAEAATPLPLPAVLDLMPSCPGAASAAAPVSDNEEGMMSAKLFGVSIGRKRMRHDGGGDDDHAATVKAEPMDGRPHGKDEQSAETQAWPIYRPRPVYQPIRACNGYEYDRAGSDQDECGAKPSNQDKKDLLRRWVVLISESLQLHGRQVVGAVQEWRRDRRGHRRPPAGHVQLRTCVNNLLVTGAGTLDAGREYETNSLPRPRR

>LOC_Os09g28200.1 (OsHsf22)

MERCGSWSDCEAAAAAAQKAVPAPFLTKTYQLVDDPATDHIVSWGDDRVSTFVVWRPPEFARDILPNYFKHNNFSSFVRQLNTYGFRKVVPERWEFANEFFRKGEKQLLTEIHRRKTSSASTASPSPPPFFAPPHFPLFHHPGVAAAQHHHAFVGDDGVVAAHGIGMPFPQPHWREPNLPVATRLLALGGPAPSPSSAEAGGAGRAATAAVLMEENERLRRSNTALLQELAHMRKLYNDIIYFVQNHVRPVAPSPAAAAFLQGLGMQARKKPAAANVLNNSGGSTTSSSSLTIAEEPSPPPQQQHLAGEKSGGEAGNSSAARSSAPTKLFGVHLSAAPCGAGSKRASSPEEHPPTSPATKPRLVLECDDLSLTVAPSSSSQQQLSAASSPTSTS

>LOC_Os09g28354.1 (OsHsf23)

MAAAEAAAAVGKQQQKGGGGRGGGGGGPAPFLTKTNQMVEESATDEVISWGKEGRSFVVWKPVEFARDLLPLHFKHCNFSSFVRQLNTYGFRKVVPDRWEFANGNFRRGEQGLLSGIRRRKATTPQSSKSCGSGVNVAFPPPLPPLPPEPSATTSSGNDRSSSSASSPPRADITSENEQLRKDNQTLTMELARARRHCEELLGFLSRFLDVRQLDLRLLMQEDMRAAAGGVGGEQRVQEHAREEKCVKLFGVLLDDTHGAATRKRARCEEAAASERPIKMIRIGEPWVSVPSSGPARCGGDN

>LOC_Os09g35790.1 (OsHsf24)

MAEQGAGEADAGGGEPPPAAVMTAAAEALAGQRSLPTPFLTKTYQLVEDPAVDDVISWNEDGSTFVVWRPAEFARDLLPKYFKHNNFSSFVRQLNTYGFRKIVPDRWEFANDCFRRGEKRLLCDIHRRKVVAAAAAAPPPPSPGMATAAAAVASGAVTVAAAPIPMALPVTRAGSPAHSSEEQVLSSNSGSGEEHRQASGSGSAPGGGGGGSASGGDMGEENERLRRENARLTRELGHMKKLCNNILLLMSKYAATQHVEGSAGISSIANCSGESSEAVPPPPPLPPAILDLMPSCPALATAAAAAGLAIDGEPDPSARLFGVSIGLKRTRDDAAAAADEDGGGEDQAEHGGADVKPEAADPHPAGGGGGSSTEASPESHPWPIYRPTPMYHAVRPTCNGPDRAGSDQDGSSSSQTMGPGEFDDLQKMMVVQQSNFVMHWGRSECGSGVRGFGW

>LOC_Os10g28340.1 (OsHsf25)

MDPAAAGIVKEEMLESQQQQRQEDGGAAPRPMEGLHEVGPPPFLTKTYDLVEDPATDGVVSWSRAGNSFVVWDPHVFADLLLPRLFKHNNFSSFVRQLNTYGFRKVDPDRWEFANEGFLRGQRHLLKTIKRRKPPSNAPPSQQQSLTSCLEVGEFGFEEEIDRLKRDKNILITEVVKLRQEQQATKDHVKAMEDRLRAAEQKQVQMMGFLARAMRNPEFFQQLAQQKEKRKELEDAISKKRRRPIDNVPFYDPGETSQTEQLDSPYLFDSGVLNELSEPGIPELENLAVNIQDLGKGKVDEERQNQTNGQAELGDDFWAELLVEDFTGKEEQSELDGKIDGIDELAQQLGYLSSTSPK

>AT1G32330.1 (AtHsf01)

MDVSKVTTSDGGGDSMETKPSPQPQPAAILSSNAPPPFLSKTYDMVDDHNTDSIVSWSANNNSFIVWKPPEFARDLLPKNFKHNNFSSFVRQLNTYGFRKVDPDRWEFANEGFLRGQKHLLQSITRRKPAHGQGQGHQRSQHSNGQNSSVSACVEVGKFGLEEEVERLKRDKNVLMQELVRLRQQQQSTDNQLQTMVQRLQGMENRQQQLMSFLAKAVQSPHFLSQFLQQQNQQNESNRRISDTSKKRRFKRDGIVRNNDSATPDGQIVKYQPPMHEQAKAMFKQLMKMEPYKTGDDGFLLGNGTSTTEGTEMETSSNQVSGITLKEMPTASEIQSSSPIETTPENVSAASEATENCIPSPDDLTLPDFTHMLPENNSEKPPESFMEPNLGGSSPLLDPDLLIDDSLSFDIDDFPMDSDIDPVDYGLLERLLMSSPVPDNMDSTPVDNETEQEQNGWDKTKHMDNLTQQMGLLSPETLDLSRQNP

>AT1G46264.1 (AtHsf02)

MAMMVENSYGGYGGGGGERIQLMVEGQGKAVPAPFLTKTYQLVDDPATDHVVSWGDDDTTFVVWRPPEFARDLLPNYFKHNNFSSFVRQLNTYGFRKIVPDRWEFANEFFKRGEKHLLCEIHRRKTSQMIPQQHSPFMSHHHAPPQIPFSGGSFFPLPPPRVTTPEEDHYWCDDSPPSRPRVIPQQIDTAAQVTALSEDNERLRRSNTVLMSELAHMKKLYNDIIYFVQNHVKPVAPSNNSSYLSSFLQKQQQQQPPTLDYYNTATVNATNLNALNSSPPTSQSSITVLEDDHTNHHDQSNMRKTKLFGVSLPSSKKRSHHFSDQTSKTRLVLDQSDLALNLMTASTR

>AT1G67970.1 (AtHsf03)

MVKSTDGGGGSSSSSSVAPFLRKCYDMVDDSTTDSIISWSPSADNSFVILDTTVFSVQLLPKYFKHSNFSSFIRQLNIYGFRKVDADRWEFANDGFVRGQKDLLKNVIRRKNVQSSEQSKHESTSTTYAQEKSGLWKEVDILKGDKQVLAQELIKVRQYQEVTDTKMLHLEDRVQGMEESQQEMLSFLVMVMKNPSLLVQLLQPKEKNTWRKAGEGAKIVEEVTDEGESNSYGLPLVTYQPPSDNNGTAKSNSNDVNDFLRNADMLKFCLDENHVPLIIPDLYDDGAWEKLLLLSPSRKKTKKQENIVKKGKDDLTLEEEEEDGTMELDKSYMLKLISEEMEKPDDFEFGQLTPERSRNLEILTEQMELLASNE

>AT1G77570.1

MLKSRNPNYSKILICEVCEVLGMGYNFYMRVYEVVDDASTDAIISWSESNNSFIIWNVGEFYRRILPKYVDLGTNLSRFFSNLRSHGFKIVKGRTGVLEFGHEDFIRDKLELMKKMVSDKRKARKAAKSKARKARVQVEFLFQHLQI

>AT2G26150.1 (AtHsf04)

MEELKVEMEEETVTFTGSVAASSSVGSSSSPRPMEGLNETGPPPFLTKTYEMVEDPATDTVVSWSNGRNSFVVWDSHKFSTTLLPRYFKHSNFSSFIRQLNTYGFRKIDPDRWEFANEGFLAGQKHLLKNIKRRRNMGLQNVNQQGSGMSCVEVGQYGFDGEVERLKRDHGVLVAEVVRLRQQQHSSKSQVAAMEQRLLVTEKRQQQMMTFLAKALNNPNFVQQFAVMSKEKKSLFGLDVGRKRRLTSTPSLGTMEENLLHDQEFDRMKDDMEMLFAAAIDDEANNSMPTKEEQCLEAMNVMMRDGNLEAALDVKVEDLVGSPLDWDSQDLHDMVDQMGFLGSEP

>AT2G41690.1 (AtHsf05)

MEDAGEHLRCNDNVNDEERLPLEFMIGNSTSTAELQPPPPFLVKTYKVVEDPTTDGVISWNEYGTGFVVWQPAEFARDLLPTLFKHCNFSSFVRQLNTYGFRKVTTIRWEFSNEMFRKGQRELMSNIRRRKSQHWSHNKSNHQVVPTTTMVNQEGHQRIGIDHHHEDQQSSATSSSFVYTALLDENKCLKNENELLSCELGKTKKKCKQLMELVERYRGEDEDATDESDDEEDEGLKLFGVKLE

>AT3G02990.1 (AtHsf06)

MGTVCESVATAKSSTAVMSSIPPFLSKTYDMVDDPLTDDVVSWSSGNNSFVVWNVPEFAKQFLPKYFKHNNFSSFVRQLNTYGFRKVDPDRWEFANEGFLRGQKQILKSIVRRKPAQVQPPQQPQVQHSSVGACVEVGKFGLEEEVERLQRDKNVLMQELVRLRQQQQVTEHHLQNVGQKVHVMEQRQQQMMSFLAKAVQSPGFLNQFSQQSNEANQHISESNKKRRLPVEDQMNSGSHGVNGLSRQIVRYQSSMNDATNTMLQQIQQMSNAPSHESLSSNNGSFLLGDVPNSNISDNGSSSNGSPEVTLADVSSIPAGFYPAMKYHEPCETNQVMETNLPFSQGDLLPPTQGAAASGSSSSDLVGCETDNGECLDPIMAVLDGALELEADTLNELLPEVQDSFWEQFIGESPVIGETDELISGSVENELILEQLELQSTLSNVWSKNQQMNHLTEQMGLLTSDALRK

>AT3G22830.1 (AtHsf07)

MDPSFRFIKEEFPAGFSDSPSPPSSSSYLYSSSMAEAAINDPTTLSYPQPLEGLHESGPPPFLTKTYDLVEDSRTNHVVSWSKSNNSFIVWDPQAFSVTLLPRFFKHNNFSSFVRQLNTYGFRKVNPDRWEFANEGFLRGQKHLLKNIRRRKTSNNSNQMQQPQSSEQQSLDNFCIEVGRYGLDGEMDSLRRDKQVLMMELVRLRQQQQSTKMYLTLIEEKLKKTESKQKQMMSFLARAMQNPDFIQQLVEQKEKRKEIEEAISKKRQRPIDQGKRNVEDYGDESGYGNDVAASSSALIGMSQEYTYGNMSEFEMSELDKLAMHIQGLGDNSSAREEVLNVEKGNDEEEVEDQQQGYHKENNEIYGEGFWEDLLNEGQNFDFEGDQENVDVLIQQLGYLGSSSHTN

>AT3G24520.1 (AtHsf08)

MEDDNSNNNNNNNVIAPFIVKTYQMVNDPSTDWLITWGPAHNSFIVVDPLDFSQRILPAYFKHNNFSSFVRQLNTYGFRKVDPDRWEFANEHFLRGQKHLLNNIARRKHARGMYGQDLEDGEIVREIERLKEEQRELEAEIQRMNRRIEATEKRPEQMMAFLYKVVEDPDLLPRMMLEKERTKQQQQVSDKKKRRVTMSTVKSEEEEVEEDEGRVFRVMSSSTPSPSSTENLYRNHSPDGWIVPMTQGQFGSYETGLVAKSMLSNSTSSTSSSLTSTFSLPESVNGGGGGGCGSIQGERRYKETATFGGVVESNPPTTPPYPFSLFRGGF

>AT3G51910.1 (AtHsf09)

MMNPFLPEGCDPPPPPQPMEGLHENAPPPFLTKTFEMVDDPNTDHIVSWNRGGTSFVVWDLHSFSTILLPRHFKHSNFSSFIRQLNTYGFRKIEAERWEFANEEFLLGQRQLLKNIKRRNPFTPSSSPSHDACNELRREKQVLMMEIVSLRQQQQTTKSYIKAMEQRIEGTERKQRQMMSFLARAMQSPSFLHQLLKQRDKKIKELEDNESAKRKRGSSSMSELEVLALEMQGHGKQRNMLEEEDHQLVVERELDDGFWEELLSDESLASTS

>AT3G63350.1 (AtHsf10)

MDPSSSSRARSMPPPVPMEGLQEAGPSPFLTKTFEMVGDPNTNHIVSWNRGGISFVVWDPHSFSATILPLYFKHNNFSSFVRQLNTYGFRKIEAERWEFMNEGFLMGQRDLLKSIKRRTSSSSPPSLNYSQSQPEAHDPGVELPQLREERHVLMMEISTLRQEEQRARGYVQAMEQRINGAEKKQRHMMSFLRRAVENPSLLQQIFEQKRDREEAAMIDQAGLIKMEEVEHLSELEALALEMQGYGRQRTDGVERELDDGFWEELLMNNENSDEEEANVKQD

>AT4G11660.1 (AtHsf11)

MPGEQTGETPTVAGVGGGGAGCSAGNSGGSSGCGAGGGGGGSGGGGGGGGDSQRSIPTPFLTKTYQLVEDPVYDELISWNEDGTTFIVWRPAEFARDLLPKYFKHNNFSSFVRQLNTYGFRKVVPDRWEFSNDCFKRGEKILLRDIQRRKISQPAMAAAAAAAAAAVAASAVTVAAVPVVAHIVSPSNSGEEQVISSNSSPAAAAAAIGGVVGGGSLQRTTSCTTAPELVEENERLRKDNERLRKEMTKLKGLYANIYTLMANFTPGQEDCAHLLPEGKPLDLLPERQEMSEAIMASEIETGIGLKLGEDLTPRLFGVSIGVKRARREEELGAAEEEDDDRREAAAQEGEQSSDVKAEPMEENNSGNHNGSWLELGK

>AT4G13980.1 (AtHsf12)

MNGALGNSSASVSGGEGAGGPAPFLVKTYEMVDDSSTDQIVSWSANNNSFIVWNHAEFSRLLLPTYFKHNNFSSFIRQLNTYGFRKIDPERWEFLNDDFIKDQKHLLKNIHRRKPIHSHSHPPASSTDQERAVLQEQMDKLSREKAAIEAKLLKFKQQKVVAKHQFEEMTEHVDDMENRQKKLLNFLETAIRNPTFVKNFGKKVEQLDISAYNKKRRLPEVEQSKPPSEDSHLDNSSGSSRRESGNIFHQNFSNKLRLELSPADSDMNMVSHSIQSSNEEGASPKGILSGGDPNTTLTKREGLPFAPEALELADTGTCPRRLLLNDNTRVETLQQRLTSSEETDGSFSCHLNLTLASAPLPDKTASQIAKTTLKSQELNFNSIETSASEKNRGRQEIAVGGSQANAAPPARVNDVFWEQFLTERPGSSDNEEASSTYRGNPYEEQEEKRNGSMMLRNTKNIEQLTL

>AT4G17750.1 (AtHsf13)

MFVNFKYFSFFIRTKMDGVTGGGTNIGEAVTAPPPRNPHPATLLNANSLPPPFLSKTYDMVEDPATDAIVSWSPTNNSFIVWDPPEFSRDLLPKYFKHNNFSSFVRQLNTYGFRKVDPDRWEFANEGFLRGQKHLLKKISRRKSVQGHGSSSSNPQSQQLSQGQGSMAALSSCVEVGKFGLEEEVEQLKRDKNVLMQELVKLRQQQQTTDNKLQVLVKHLQVMEQRQQQIMSFLAKAVQNPTFLSQFIQKQTDSNMHVTEANKKRRLREDSTAATESNSHSHSLEASDGQIVKYQPLRNDSMMWNMMKTDDKYPFLDGFSSPNQVSGVTLQEVLPITSGQSQAYASVPSGQPLSYLPSTSTSLPDTIMPETSQIPQLTRESINDFPTENFMDTEKNVPEAFISPSPFLDGGSVPIQLEGIPEDPEIDELMSNFEFLEEYMPESPVFGDATTLENNNNNNNNNNNNNNNNNNNNTNGRHMDKLIEELGLLTSETEH

>AT4G18870.1 (AtHsf14)

MSKNEGSLTSVSYFITTTYDMVDDLSLDSIISWSQSGKSFIIWNPEEFYNNLLQRFCFQRINTFFSFLFSHGFRKIDSGKWEFANDNFVRGQRHLINNIISDVIEQEVQYDQGMELFKAEKLFARLVKKVQDQLPPHNSYPTSKLPFPTKIYEMVDDPSSDAIISWSQSGKSFIIWNPQEFCKDHLRRLFNTLHIHFFFYKLKIFGFKKINPKKWEFANDNFVRGQRHLVEIIISNDKKKNDQLRKQDAREKKMAEAGELFKLQIEEMSDMRKKMKKAKEVKEQEVRLCKL

>AT4G18880.1 (AtHsf15)

MDENNHGVSSSSLPPFLTKTYEMVDDSSSDSIVSWSQSNKSFIVWNPPEFSRDLLPRFFKHNNFSSFIRQLNTYGFRKADPEQWEFANDDFVRGQPHLMKNIHRRKPVHSHSLPNLQAQLNPLTDSERVRMNNQIERLTKEKEGLLEELHKQDEEREVFEMQVKELKERLQHMEKRQKTMVSFVSQVLEKPGLALNLSPCVPETNERKRRFPRIEFFPDEPMLEENKTCVVVREEGSTSPSSHTREHQVEQLESSIAIWENLVSDSCESMLQSRSMMTLDVDESSTFPESPPLSCIQLSVDSRLKSPPSPRIIDMNCEPDGSKEQNTVAAPPPPPVAGANDGFWQQFFSENPGSTEQREVQLERKDDKDKAGVRTEKCWWNSRNVNAITEQLGHLTSSERS

>AT4G19630.1

MGQNFPNGLGTFYIGIYKLVEDPSSDPIISWSKSNNGFVMCNEEARIRSKILLRFNCGKLSEFLSELKYYGFTRVKKTDSGKMEFRNEDFVRGQPERLRDMMLKACRKHRAKFKAKEAAKEAAKELQRLQI

>AT4G36990.1 (AtHsf16)

MTAVTAAQRSVPAPFLSKTYQLVDDHSTDDVVSWNEEGTAFVVWKTAEFAKDLLPQYFKHNNFSSFIRQLNTYGFRKTVPDKWEFANDYFRRGGEDLLTDIRRRKSVIASTAGKCVVVGSPSESNSGGGDDHGSSSTSSPGSSKNPGSVENMVADLSGENEKLKRENNNLSSELAAAKKQRDELVTFLTGHLKVRPEQIDKMIKGGKFKPVESDEESECEGCDGGGGAEEGVGEGLKLFGVWLKGERKKRDRDEKNYVVSGSRMTEIKNVDFHAPLWKSSKVCN

>AT5G03720.1 (AtHsf17)

MSPKKDAVSKPTPISVPVSRRSDIPGSLYVDTDMGFSGSPLPMPLDILQGNPIPPFLSKTFDLVDDPTLDPVISWGLTGASFVVWDPLEFARIILPRNFKHNNFSSFVRQLNTYGFRKIDTDKWEFANEAFLRGKKHLLKNIHRRRSPQSNQTCCSSTSQSQGSPTEVGGEIEKLRKERRALMEEMVELQQQSRGTARHVDTVNQRLKAAEQRQKQLLSFLAKLFQNRGFLERLKNFKGKEKGGALGLEKARKKFIKHHQQPQDSPTGGEVVKYEADDWERLLMYDEETENTKGLGGMTSSDPKGKNLMYPSEEEMSKPDYLMSFPSPEGLIKQEETTWSMGFDTTIPSFSNTDAWGNTMDYNDVSEFGFAAETTSDGLPDVCWEQFAAGITETGFNWPTGDDDDNTPMNDP

>AT5G16820.1 (AtHsf18)

MESVPESVPSPNSNTPSIPPPVNSVPPFLSKTYDMVDDPLTNEVVSWSSGNNSFVVWSAPEFSKVLLPKYFKHNNFSSFVRQLNTYGFRKVDPDRWEFANEGFLRGRKQLLKSIVRRKPSHVQQNQQQTQVQSSSVGACVEVGKFGIEEEVERLKRDKNVLMQELVRLRQQQQATENQLQNVGQKVQVMEQRQQQMMSFLAKAVQSPGFLNQLVQQNNNDGNRQIPGSNKKRRLPVDEQENRGDNVANGLNRQIVRYQPSINEAAQNMLRQFLNTSTSPRYESVSNNPDSFLLGDVPSSTSVDNGNPSSRVSGVTLAEFSPNTVQSATNQVPEASLAHHPQAGLVQPNIGQSPAQGAAPADSWSPEFDLVGCETDSGECFDPIMAVLDESEGDAISPEGEGKMNELLEGVPKLPGIQDPFWEQFFSVELPAIADTDDILSGSVENNDLVLEQEPNEWTRNEQQMKYLTEQMGLLSSEAQRK

>AT5G43840.1 (AtHsf19

MDYNLPIPLEGLKETPPTAFLTKTYNIVEDSSTNNIVSWSRDNNSFIVWEPETFALICLPRCFKHNNFSSFVRQLNTYGFKKIDTERWEFANEHFLKGERHLLKNIKRRKTSSQTQTQSLEGEIHELRRDRMALEVELVRLRRKQESVKTYLHLMEEKLKVTEVKQEMMMNFLLKKIKKPSFLQSLRKRNLQGIKNREQKQEVISSHGVEDNGKFVKAEPEEYGDDIDDQCGGVFDYGDELHIASMEHQGQGEDEIEMDSEGIWKGFVLSEEEMCDLVEHFI

>AT5G45710.1 (AtHsf20)

MDENNGGSSSLPPFLTKTYEMVDDSSSDSVVAWSENNKSFIVKNPAEFSRDLLPRFFKHKNFSSFIRQLNTYGFRKVDPEKWEFLNDDFVRGRPYLMKNIHRRKPVHSHSLVNLQAQNPLTESERRSMEDQIERLKNEKEGLLAELQNQEQERKEFELQVTTLKDRLQHMEQHQKSIVAYVSQVLGKPGLSLNLENHERRKRRFQENSLPPSSSHIEQVEKLESSLTFWENLVSESCEKSGLQSSSMDHDAAESSLSIGDTRPKSSKIDMNSEPPVTVTAPAPKTGVNDDFWEQCLTENPGSTEQQEVQSERRDVGNDNNGNKIGNQRTYWWNSGNVNNITEKAS

>AT5G54070.1 (AtHsf21)

MTAIPNVVDIESSSSSLCQETATETVTVERGSSDSSSKPDDVVLLIKEEEDDAVNLSLGFWKLHEIGLITPFLRKTFEIVDDKVTDPVVSWSPTRKSFIIWDSYEFSENLLPKYFKHKNFSSFIRQLNSYGFKKVDSDRWEFANEGFQGGKKHLLKNIKRRSKNTKCCNKEASTTTTETEVESLKEEQSPMRLEMLKLKQQQEESQHQMVTVQEKIHGVDTEQQHMLSFFAKLAKDQRFVERLVKKRKMKIQRELEAAEFVKKLKLLQDQETQKNLLDVEREFMAMAATEHNPEPDILVNNQSGNTRCQLNSEDLLVDGGSMDVNGRIEIE

>AT5G62020.1 (AtHsf22)

MNSPPVDAMITGESSSQRSIPTPFLTKTFNLVEDSSIDDVISWNEDGSSFIVWNPTDFAKDLLPKHFKHNNFSSFVRQLNTYGFKKVVPDRWEFSNDFFKRGEKRLLREIQRRKITTTHQTVVAPSSEQRNQTMVVSPSNSGEDNNNNQVMSSSPSSWYCHQTKTTGNGGLSVELLEENEKLRSQNIQLNRELTQMKSICDNIYSLMSNYVGSQPTDRSYSPGGSSSQPMEFLPAKRFSEMEIEEEEEASPRLFGVPIGLKRTRSEGVQVKTTAVVGENSDEETPWLRHYNRTNQRVCN

>Bradi1g66590.1 (cHSP70-1)

MAGKGDGPAIGIDLGTTYSCVGVWQHDRVEIIANDQGNRTTPSYVAFTDTERLIGDAAKNQVAMNPINTVFDAKRLIGRRFSDASVQSDAKLWPFKVIPGPGDKPMIGVQYRGEDKQFSAEEISSMVLNKMKETAEAYLGTTIKNAVVTVPAYFNDSQRQATKDAGVISGLNVMRIINEPTAAAIAYGLDKKSTSVGEKNVLIFDLGGGTFDVSLLTIEEGIFEVKATAGDTHLGGEDFDNRMVNHFVQEFKRKNKKDISGNPRALRRLRTACERAKRTLSSTAQTTIEIDSLFEGIDFYTTITRARFEELNMDLFRKCMEPVEKCLRDAKMDKSTVHDVVLVGGSTRIPRVQQLLQDFFNGKELCKSINPDEAVAYGAAVQAAILTGEGNEKVQDLLLLDVTPLSQGLETAGGVMTVLIPRNTTIPTKKEQVFSTYSDNQPGVLIQVYEGERARTKDNNLLGKFELSGIPPAPRGVPQITVCFDIDANGILNVSAEDKTTGQKNKITITNDKGRLSKEDIEKMVQEAEKYKAEDEEHKKKVDAKNSLENYAYNMRNTIKDDKIASKLPEADKKKIEDAIDGAITWLDNNQLAEADEFDDKMKELEGICNPIIAKMYQGAGAEMPGGMDEDTPASTAGGSSGPGPKIEEVD

>Bradi2g23250.1 (cHSP70-2)

MAKGEGPAIGIDLGTTYSCVGVWQHDRVEIIANDQGNRTTPSYVAFTDTERLIGDAAKNQVAMNPTNTVFDAKRLIGRRFSDPSVQSDMKLWPFKVIPGPGDKPMIVVQHKGEEKQFAAEEISSMVLIKMREIAEAYLGNSIKNAVVTVPAYFNDSQRQATKDAGVIAGLNVMRIINEPTAAAIAYGLDKKATSTGEKNVLIFDLGGGTFDVSLLTIEEGIFEVKATAGDTHLGGEDFDNRMVNHFVQEFKRKHKKDISGNPRALRRLRTACERAKRTLSSTAQTTIEIDSLYEGVDFYTTITRARFEELNMDLFRKCMEPVEKCLRDAKMDKSSVHDVVLVGGSTRIPKVQQLLQDFFNGKELCKSINPDEAVAYGAAVQAAILSGEGNEKVQDLLLLDVTPLSLGLETAGGVMTTLIPRNTTIPTKKEQVFSTYSDNQPGVLIQVYEGERARTKDNNLLGKFELSGIPPAPRGVPQITVCFDIDANGILNVSAEDKTTGQKNKITITNDKGRLSKEEIEKMVQEAEKYKAEDEEHKKKVDAKNALENYAYNMRNTIKDEKIASKLGADDKKKVEDAIDGAISWLDTNQLAEADEFEDKMKELEGICNPIIAKMYQGAAPDMGGGMGMDEDAPAGGSSGAGPKIEEVD

>Bradi1g03720.1 (cHSP70-3)

MAPTKGEGPAIGIDLGTTYSCVGVWQHDRVEIIANDQGNRTTPSYVGFTDTERLIGDAAKNQVAMNPINTVFDAKRLIGRRFSDAPVQSDIKMWPYKVISGPADKPMIVVQYKGEDKQFSAEEISSMVLIKMREIAEAYLGVTIKNAVVTVPAYFNDSQRQATKDAGVIAGLNVMRIINEPTAAAIAYGLDKKATSVGEKNVLIFDLGGGTFDVSLLTIEEGIFEVKATAGDTHLGGEDFDNRLVNHFVQEFKRKHKKDISGNPRSLRRLRTSCERAKRTLSSTAQTTIEIDSLYEGVDFYSTITRARFEELNMDLFRKCMEPVEKCLRDAKMDKSTIHDVVLVGGSTRIPRVQQLLQDFFNGKELCKSINPDEAVAYGAAVQAAILSGEGNEKVQDLLLLDVTPLSLGLETAGGVMTVLITRNTTIPTKKEQVFSTYSDNQPGVLIQVFEGERTRTRDNNLLGKFELSGIPPAPRGVPQITVCFDIDANGILNVSAEDKTTGQKNKITITNDKGRLSKDDIEKMVQDAEKYKSEDEEHKKKVEAKNTLENYAYNMRNTISDEKIASKLPADDKKKIEDAVDQAIQWLDNNQLAEVDEFEDKMKELEGLCNPIIAKMYQGAGADMPGGMDEDAPAASGGAGPKIEEVD

>Bradi4g04220.1 (cHSP70-4)

MAAKGDGPAIGIDLGTTYSCVGVWQHDRVEIIANDQGNRTTPSYVAFTDSERLIGDAAKNQVAMNPINTVFDAKRLIGRRFTDSTVQSDIKLWPFKVVAGPGDKPMINVQYKGEEKQFAAEEISSMVLIKMREIAEAFLGTTVKNAVVTVPAYFNDSQRQATKDAGVIAGINVLRIINEPTAAAIAYGLDKKASSVGEKNVLIFDLGGGTFDVSLLTIEEGIFEVKATAGDTHLGGEDFDNRMVNHFVQEFKRKNKKDISGNPRALRRLRTSCERAKRTLSSTAQTTIEIDSLFEGIDFYSTITRARFEEMNMDLFRKCMEPVEKCLRDAKMDKSTVHDVVLVGGSTRIPRVQQLLQDFFNGKELCKSINPDEAVAYGAAVQAAILSGEGNEKVQDLLLLDVTPLSLGLETAGGVMTVLIPRNTTIPTKKEQVFSTYSDNQPGVLIQVYEGERTRTRDNNLLGKFELSGIPPAPRGVPQITVCFDIDANGILNVSAEDKTTGQKNKITITNDKGRLSKEEIEKMVQEAEKYKSEDEEHKKKVESKNALENYSYNMRNTIKDEKIASKLPADDKKKIEDAIDAAIQWLDTNQLAEADEFEDKMKELESLCNPIIAKMYQGAGADMEGSGMDEDTPAASGGPGPKIEEVD

>Bradi2g54570.1 (cHSP70-5)

MAKGGEGPAIGIDLGTTYSCVGVWQHDRVEIIANDQGNRTTPSYVGFTDTERLIGDAAKNQVAMNPTNTVFDAKRLIGRRFSDPSVQSDMKLWPFKVIPGPGDKPMIVVQYKGEEKQFAAEEISSMVLIKMKEIGEAYLGTTIKNAVVTVPAYFNDSQRQATKDAGVIAGLNVMRIINEPTAAAIAYGLDKKASSSGEKNVLIFDLGGGTFDVSLLTIEEGIFEVKATAGDTHLGGEDFDNRMVNHFVQEFKRKHKKDISGNPRALRRLRTACERAKRTLSSTAQTTIEIDSLYEGVDFYSTITRARFEELNMDLFRKCMEPVEKCLRDAKMDKSTVHDVVLVGGSTRIPKVQQLLQDFFNGKELCKSINPDEAVAYGAAVQAAILSGEGNEKVQDLLLLDVTPLSLGLETAGGVMTVLIPRNTTIPTKKEQVFSTYSDNQPGVLIQVFEGERARTKDNNLLGKFELSGIPPAPRGVPQITVCFDIDANGILNVSAEDKTAGLKNKITITNDKGRLSKEEIEKMVQEAERYKAEDEELKKKVDAKNALENYAYNMRNTIKDDKIAAKLSAGDKKKIEDSIDGAISWLDTNQLAEADEFEDKMKELEGICNPIIAKMYQGAGADMGGMGMGGATAMDEDSPSSRSGAGPKIEEVD

>Bradi1g66470.1 (cHSP70-6)

MASKGGSNNKGEGPAIGIDLGTTYSCVGVWQHDRVEIVANDQGNRTTPSYVAFTDTERLIGDAAKNQVAMNPTNTVFDAKRLIGRRFSDPSVQADMKLWPFKVVPGAGDKPMIVVTYKGEEKKFSAEEISSMVLTKMREIAEAFLSTTINNAVVTVPAYFNDSQRQATKDAGVIAGLNVMRIINEPTAAAIAYGLDKKATSTGEKNVLIFDLGGGTFDVSILTIEEGIFEVKSTAGDTHLGGEDFDNRMVNHFVQEFRRKNKKDISGNPRALRRLRTACERAKRTLSSTAQTTIEIDSLYEGIDFYATITRARFEELNMDLFRKCMEPVEKCLRDAKMDKTQIHDIVLVGGSTRIPKVQQLLQDFFNGKELCKSINPDEAVAYGAAVQAAILSGEGNQKVQDLLLLDVTPLSLGLETAGGVMTTLIPRNTTIPTKKEQVFSTYSDNQPGVLIQVYEGERTRTKDNNLLGKFELSGIPPAPRGVPQITVTFDIDANGILNVSAEDKTTGQKNKITITNDKGRLSKEEIERMVQEAEKYKSEDEQVRQKVEARNALENYAYNMRNTVKDDKIASKLPAEDKKKIEDSIEDAIRWLDGNQLAEAEEFEDKMKELENICNPLISKMYQGGPAGMDEDVPSGGAGAGTGGGSGGAGPKIEEVD

>Bradi1g66520.1 (cHSP70-7)

MGTVGKGEGPAIGIDLGTTYSCAAVWRPSHNRVEVIPNDQGNLTTPSCVAFTDTCRLIGEAAMNQAAMNSVNTVFDAKRLIGRRFSNASVQGDIKLWPFKVISGPSDRPMIVVQYRGEEKKFAAEEISSMVLIKMQEAAEAYLGTAVKNVVITVPIYFNDSQRQATIDAGTIAGLNVMRIINEPSAAAIAYGLDKMTVSGSVKTVLIFDLGGGTLDISIINIDMGIFRVKATSGDTHLGGEDINSRMVEHFVQDFLRRHKSDIKSNPRALMQLRTACERAKRMLSSTTQAKFEIDSLHEGIDYYGIITRARFEELNMDLFRNRIPKVQELLQDFFNGKVLCRSINPDEAVAYGAAVQAAVLSGQCNQKVQDLLLLDVTPLSLGVDIVDDFYRPGVMSVIIPRNTTIPCKNAWNYTTIFDNQTSILFPVFEGEGAMTKDNNLLGQIILGGIIPAPAGVPYIDVTFEIEANGILKVSAEDMTTGNKNSITISTDKGGLNKQEIERMIWDAKKYKSADKGSKIKKENEEGWLSKEEIERMVTKKRMIQDAEKYKSEDKKRKIKKENEGWLSKEEFERMVTKKRMIQGAENYELEDKKQIM

>Bradi1g66527.1 (cHSP70-8)

MVTKGDGPAIGIDLGTTY*CVAVWRPLHNRVEVIPNDQGNHTTPSCVAFTDTCRLIGDAAVNQASMNSVNTVFDVKRLIGRQFINASVQGDIKLWPFKVISGPSDRPMIVVQYKGGGKQFTAEEISSMVLIKMRETAEAYLDKAFNDSQRQPTIDASAIAGLNVMRIINEPSAAVIAYGLDRISGNGSVKTLLIFDLGGGTLDISIISVDMGIFTVKATSGDTHLGGEDLNNRMVEHFTQDFLRRHKSDIRSNPRALMRLRTACERAKRMLSYTAQAKFEIDSLHNGIDFCGSISRARFEELNMDLYRKCIEHVEKCLSDAKMDKSRIHDVVLVGGSSRIPKVQNLLQDFFNGKNLCNSINPDEAVAYGAAVQAAILRGECSQKVQDLILLDVTPLSLGIEVVGGIMSVVIPRTTTISFKKDQIFTTAYDNQTAVAINVYERVKVR

>Bradi1g66540.1 (cHSP70-9)

MGTMGKGDGPAIGIDLGTTYSCVAVWRPSHNRVEVIPNDQGNLTMPSCVAFTDSWRLVGDAAMNQAAMNPLNTVFDAKRLIGRRFSDASVQGDMKLWPFKVISGTSARPMIVVQYKDEEKQFAAEEISSMLLVKMREAAEAYLSTMVKNVVITVPVYFNDSQRQATIDAGAIAGLNVMRIINEPSAAAIAYGLNKMSGSSGSKTVLIFDLGGGTLDISIINIDKGIFTVKATSGDTHLGGEDFNNRMVEHFVQDVLKRHNSDIRSNPRALMRLRRACERAKRMLSSTAQAKFEIDSLHEGIDYYGIITRARFEELNMELFRKCIEHIEKCLGDAKMDKSQIHEVVLVGGSSRIPKVQQLLQDFFSGKMLCKSINPDEAVAYGAAVQAAVLSGECDQKVQDLLLLDVTPLSLRIEVVGGIMNVVIPRITTIPFKKDRIYTTIYDNQTAVSINVYEGEAALIKDNNLLGKFTLCGIPPAPRLVPKINVTFEIEANCILTVSAQDMTTGIKNSITITADKGGLSKAEVERMVQDAKKYKSDDSKRKIKKENDEGWLSKEEFERMVRDAKKRKCEDDKKQIKKIKTESGGP

>Bradi1g66550.1 (cHSP70-10)

MAAGTNGDGPAIGIDLATTYSCVAVWRPAHNRVEVIANDQGNLTTPSCVAFTDTWRLIGESAMNPSNTIFDAKRLIGRRYKDPSVQEDIKLWPFKVISGPGERPIIVVQYRGEERPFAAEEISAMVLVKMRETAEAYLGSAVKNAVITVPVYFNGSQRQDTIDAGAIAGLNVMRIINEPSAAAIADGLDRMSGSSEVKTVLIFDLGGGTLDISVIDIENGKFIVQATSGDTHLGGEDLNSRMELNMDLFRKCIEHVEKCLGDAKMDKSQIHDVVLVGGSSRIPKVQQLLQDFFDGKMLCRNINPDETVAYGAAVQAAVLSGEYQQVQDMLLLDVTPLSLGIEVVGDIMSVVIPRITTIPCKKGRIYTTEYDNQTVAAINVYEGEGALIKDNNLLGKFTLEGIPPAPRLVPKINVTFEIEMNGILKVSAEDMTTGNKNSISIKTDKGGLTKEEIERMVQDAEKYKSEDKKNLTKIKKERGGT

>Bradi1g66560.1 (cHSP70-11)

MQGWCHNLSLVLVVLTNPQSIFVTDVKRLIGRHFSDSSVQADMKLWPFKVISGPSDRPMVVVQYKEEEKQFEAEQISAMVLAKMREIAEAYLDTDVKNAVITVPVYFNDSQRQATIDAGTIAGLNVMRIINEPSAAALAYGLDKIHSSDEVKTVLIFELGGGTLDVSVVNIDPGVDIDMGVFEVKAMAGDTHLGGEDFNDPMVKHFVREFLKKFKKTDIRNNPKALRWLRAACEKAKRMLSSAAQATVEINSLHDGIDFYGVITRARFEDLNMDLFRKCIEHVEKCLGDAKMDKSINPDEAVAYGAAVQAAILSDEGGEEVRDVLLLDITPLSLGVETEGREMSTLIPRNTTIPVKKEGVYTTCSNNQTKVRIQVYEGEGAATEDNHLLGKFILTGIPKAPKGVPKINVTFVIDANCVLTVSAEDMSTGKNNKIVIAKDTGRLSTEEIERMVRDADEYNAEDEKEMQKRGGA

>Bradi4g28250.1 (Bip3)

MARATWLLGLVLAALLVVASPATGEKEQAASTAKKTSSSSGPVIGIDLGTTYSCVGVYRNGHVEIIANDQGNRITPSWVAFPDSGERLIGEAAKNQAASNPSRTVYDAKRLIGRQFSDVEVQKDMKHLPYKIVEKQGKPHVELEVKDGDVRVLSPEEVSAMVLTRMKETAEAYLGHKVRDAVITIPAYFNDAQRQATKDAGAIAGLNVVRLINEPTAAAIAYGLDKINGANNDKEKNILVFDLGGGTFDVSVLAIDNGVFEVLATNGDTHLGGEDFDQRLMDYLIKLVKRKHGKDIAGDARALSKLRRESERAKRALSNQHQVRVEVESLFDGVDLSEPLTRARFEELNSDLFRKTMAPVKKAMADAGLGKGDIDEIVLVGGSTRIPRIQQLLKDYFNGKEPSKGVNPDEAVAYGAAVQGSIVRGDVEQQVVVLDVTPLTLGIETAGGVMTSLIPRNTVVPTRKTQVFSTYQDRQTTVSIKVFEGERGMTKDNKLLGKFDLTGIAPAPRGVPQIEVTFEVDVNGILHVKAADKGTGKSEKIEITSAADRRITQEEIDRMVREAEEFAEEDKKVREKVDARNKLEAYVYNVRTTVDGEAGKGMDGGDKERVQEAAREANEWLDANPDADKDDYVEKLKELEDVCNSVFAATRGGHEDGAEDDDHDEL

>Bradi3g01477.1 (Bip1)

MDRVRGSLLLLGVLLAGSLFAFSAAKEEAKKLGTVIGIDLGTTYSCVGVYKNGHVEIIANDQGNRITPSWVGFTDSERLIGEAAKNQAAVNPERTIFDVKRLIGRKFEDKEVQRDMKLVPYKIVNRDGKPYIQVKIKDGENKVFSPEEISAMILGKMKETAEAYLGKKINDAVVTVPAYFNDAQRQATKDAGVIAGLNVARIINEPTAAAIAYGLDKKGGEKNILVFDLGGGTFDVSILTIDNGVFEVLATNGDTHLGGEDFDHRIMEYFIKLIKKKYSKDISKDNRALGKLRREAERAKRALSNQHQVRVEIESLFDGTDFSEPLTRARFEELNNDLFRKTMGPVKKAMDDAGLEKSQIHEIVLVGGSTRIPKVQQLLRDYFEGKEPNKGVNPDEAVAFGAAVQGSILSGEGGDETKDILLLDVAPLTLGIETVGGVMTKLIPRNTVIPTKKSQVFTTYQDQQTTVSIQVFEGERSMTKDCRLLGKFDLSGIPPAPRGTPQIEVTFEVDANGILNVKAEDKGTGKSEKITITNEKGRLSQEEIDRMVKEAEEFAEEDKKVKERIDARNQLETYVYNMKNTVGDKDKLADKLESEEKEKVEEALKEALEWLDENQSAEKEDYEEKLKEVEAVCNPIVSAVYQRSGGAPGGEDGAGGVDDEEHDEL

>Bradi2g06050.1 (Bip2)

MARGTSTTALLLGLFLVGFLVAPSAAAADSKGDQQPANKASGGPVIGIDLGTTYSCVGVYRNGHVEIIANDQGNRITPSWVAFTDSGERLIGEAAKNQAASNPLRTIYDAKRLIGRQYGDAEVHKDMKHLPYKIVEKRGKPHMEVEVKDGDVRTLSPEEVSAMVLTRMKETAEAFLGEPVKDAVITIPAYFNDAQRQATKDAGAIAGLNVVRLINEPTAAAIAYGLDNKAKDAKEERNVLVFDLGGGTFDVSVLTIDNGVFEVLATNGDTHLGGEDFDHRLMDYLVKLVKRKHGKDVSHDARALGKLRRECERAKRALSSQHQVRVEIESLFDGVDLSEPLTRARFEELNSDLFRKTMTPVKKAMADAGLAKGDIHEVVLVGGSTRIPKIQQFLKDYFDGKEPSKGVNPDEAVAYGAAVQGSIVRGDNAEKLVVLDVTPLTLGIETAGGVMTPLIPRGTVIPTRKTKTFTTYQDRQTTVSVVVFEGERSMTKDNKQLGKFDLTGIAPAPRGTPQIEVTFEVDVNGILHVKAADKGTGKSEKIQITSAADRRITQEEIDRMVREAEEFAEEDRKVRERVDARNRMEAYVYHVRTTVDGEAGQGMDGGDKERVREAAREASEWIDENPEADKDEYVEKLKELEDLCNPVFAAADSHKSGGGHDEAEEDDHDEL

>Bradi5g17520.1 (uHsp70-1)

MVKGEGPATRIDLGTTYSCVGMWQHDRVEIIANDQGNRTTPSYVAFTDTERLIGDAKDQLWHVRLDWFCGILKLGGSLFCRMILKLDSIARNYVPSILKLGGDFILKLGTMQCRDGMPCLSGVNKEMATHGGKIARMEKKL

>Bradi2g33682.1 (Hsp110-8)

MSVVGFDLGNESCIVGVARQRGIDVVLNEESKRETPAIVCFGDKQRFIGTAGAANSTMNPKNSISQIKRLLGRKFTDPELQHDLQSFPFHVSEGPDGFPLVHARYLGEERSFTPTQLLAMVLSNLKGIAEGNLNSAVIDCCIGIPVYFTNLQRRAVLDAATIAGLRPLRLFHETTATALAYGIYKTDLPENDQLNVAFVDVGHASMQVSIVGYKKGQLKMLSHAYDRSLGGRDFDEALFKHFAAKFKEEYKIDVYQNARACIRLRVACEKLKKMLSANPEAPMNIECLMDEKDVRGFIKRDEFEQISGPVLERVKGPLEKALAEAGLTTESVHFVEVVGSGSRVPAIMRIITEFFGKEPRRTMNASECVARGCALQCAILSPTFKVREFQVNEGFPFSIALSWKSDAQSNESQQTVVFPKGNPMPSIKALTFYRSNTFAVDVLNVDTDDLQITQKISTYTIGPFQPSNGEKAKVKVKVRLNIHGIVSLESATMLEEDEVEVPVSSASEVPKDATKMDTDDAQRDPASGNDVNMEDSKGATDTAEGAVENGAHDSEEKSVPMDTDTKVQPSKKRVKKTNVPIAELVYGTLGADELEKAVEKEYEMALQDRVMEETKEKKNSVEAYVYEMRNKLSEKYNDFVMSEDMEVLMAKLQEVEDWLYEDGEDETKGVYVAKLEELKKVGGPIEMRYKEWSERGQALEQLVYCIRSFREAALSSDQKFDHIDISEKQKVVNECSGAETWLLEKKQQQDALPKHVNPVLLVSDIKKKAEALDRFCKPIMTKPKPAPKPQTPPPVETPAPEAQTPEQQSSGAGEADEPANDGGAQDQPAAEQMDTDSAEPSSA

>Bradi5g05900.1 (Hsp110-7)

MPICLLLRKVLFRHHTRGWMTFSFLDFFSTVQPKVATVGIDFGCKNSRVAIVDSLIVWRVIIVVAKSARTTTTLDKVPEVVPSETGCSIPSYVTPIDPKDSDGYGWALQQLDRLGKCVAVGELAKRRLSRQPSDVVFNIKKLAGKQFDDHNVQEMRKRVHFSIVEGERGEAWVEICRMKFSPVEIASVIFARLKDIILMHQFHHEFKVVISVPIFFNQQQRKEIMLAGHKAGLEILQIIDEPIAAALSSTTIKEGTIVVFDMGAGSYNVSILGVSGTKIEIKTQFGNPCVGGDQFDDILLDYSVAQIRKFYSVDVCGDKYGMMLLAEAVEQAKVALSSQHEVTVSLPFIISSAKCPGDPNISISRAEFENLGVNLVKQIRDKCQTLLAEANISSNDIDEVILTGGMTRVPMIQKIIFDVFGKHQATKVNHEEAVVIGSAIQACLIVEHQRQISEDIIPLSIGIESEGIFVRVIPRHSTIPTKKTVKIPAWRGYGESLPINVYLGEHVLVQHNVFLGVVELINNQRSCQGSIYFELTFEVDKDYVVKVGGRNFGDQHEAAYDLVKPLKVFPVREIVMCKQSVDKAVKSALLDWTMHGIDFRARLIRQATYIVSTLSDVLSARKDEVPKDLFEEAAKSLTNLLMTLDGDAHVLNEKMLAAESAKLKVLQWMPPSESPCRDYSDYED

>Bradi2g46937.2 (uHsp70-2)

MAAAADPRAKTPATPSSHHLKPWVPPPRGSRVPSLLPAVSGASRDRRRSSTSSSHRRGGDASAADEEPYDAGLEDLRAKLMGHLHDAADRLRLPQAHKSHRSPAPAEPKPPLPPPPPPPPAPPQDAAAAVVAAASMPWTLRERKRRPSARGSAAAAAAQATTTAAAGTARDDGERAPFALALEAEEIEEDIYALTGARPRRRPRKRSRIVQRQLDLRKPEAQIDYPPFRLTSPHRPLLVALAARSAFPLDRWQVHPSLQLPPAVLRMTGTGDQQEGGTIAIGIDLGTSWSCVGVYRHRHGRVEIIINEQGSRTTPSCVAFSDTESLLVGEAALNQAARNPTNTVSGAKRLPGRRFSDASVRSDMNLWPFKVVAGRGDKPMIAASHRRRQKLLAGEEIAAMLLVKIKRDAESYLGGTVTNAVVTVPVSFDVLQRRATKDAFAVAGLDVLGVVHEPVAAAVAYYGLFHESGTETKNVVVFDLGGGHTSAALLTISAGKIAVEATAGDAHLGGEDFDGRMVEHFVEQLKTEYGKEDVGRSARALVRLRAACEQAKRTLSSSTWAPIEIDCLLEGVDFRTTITRDQFEDLNTDLFCRCMEPVKKCLSDAKLDRSNVHDVVLVGGSTRIPCLRRMLRDLFDGKELLRKDINPEEAVARGAAILAAAVVSRVPDSDLLDLILSDTTPRSLGVEAAGGAMAVIIPKNSTIPIRREQIIALHSQEPTSVVIPVFEGENPIARENSLLGELKLSAVHRGTQRGGSERQVSVCFDIDADGVMTVNARDRATKNANQMKFMDKGQLSKQEIERMAEEAAEYMAQDAENRDRVNAKNLLEECLYVKRRKIEAERKKANDALSGLEQMIQQVDNDQVSSAKKFREDLEVLMVEGSTVAGKLGDA

>Bradi2g30560.1 (cpHSP70-1)

MATTTFPTSTPFFVNHGSRRPSVNVRTAAAVYGRGGRRWRPLRVACEKVVGIDLGTTNSAVAAMEGGKPTIVTNAEGARTTPSVVAYTKAGDRLVGQIAKRQAVVNPENTFFSVKRFIGRKMNEVAEESKQVSYRIIRDDNGNVKLDCPAIGKQFAAEEISAQVLRKLVDDASKFLNDKVTKAVITVPAYFNDSQRTATKDAGRIAGLDVLRIINEPTAASLAYGFEKKNNETILVFDLGGGTFDVSVLEVGDGVFEVLSTSGDTHLGGDDFDKRIVDWLAGSFKNDEGIDLLKDKQALQRLTEAAEKAKMELSSLTQTNISLPFITATADGPKHIETTLTRAKFEELCSDLLDRLRTPVDNSLRDAKLSLKEIDEVILVGGSTRIPAVQDLVKKMTGKDPNVTVNPDEVVALGAAVQAGVLSGDVSDIVLLDVTPLSLGLETLGGVMTKIIPRNTTLPTSKSEVFSTAADGQTSVEINVLQGEREFVRDNKSLGSFRLDGIPPAPRGVPQIEVKFDIDANGILSVAAVDKGTGKKQDITITGASTLPKDEVEKMVEEAEKFAAEDKEKRDAIDTKNQAESVIYQTEKQLKELGDKVPGEVKGKVEGKLVELKDAVAGGTTQTIKDALAALNQEVMQLGQSLYQQQGAPGAGPTPGGDGTADSGPSEKPGDDGDVIDADFTDSK

>Bradi4g39470.1 (cpHSP70-2)

MATFTPQVSAMACGSPSSSLFVGRRRRPALQMRAPRGGRARGLAMRVACEKVVGIDLGTTNSAVAAMEAGKPTVITNAEGQRTTPSVVAYTKGGERLVGQIAKRQAVVNPENTFFSVKRFIGRKMAEVDDEAKQVSYNVLRDENGNVKLDCPAIGKQFAAEEISAQVLRKLVDDASKFLNEKITKAVVTVPAYFNDSQRTATKDAGRIAGLEVLRIINEPTAASLAYGFEKKNNETILVFDLGGGTFDVSVLEVGDGVFEVLSTSGDTHLGGDDFDKKIVDWLASTFKNDEGIDLLKDKQALQRLTEAAEKAKMELSTLTQANISLPFITATADGPKHIEATLSRAKFEELCSDLIDRLKTPVNNALKDAKLSVSNLDEVILVGGSTRIPSVQELVKKITGKDPNVTVNPDEVVSLGAAVQGGVLAGDVKDVVLLDVTPLSIGLETLGGVMTKIIPRNTTLPTSKSEVFSTAADGQTSVEINVLQGEREFVRDNKSLGSFRLDGIPPAPRGVPQIEVKFDIDANGILSVAAVDKGTGKKQDITITGASTLPKDEVERMVEEADKFAQEDKEKRDAIDTKNQADSVVYQTEKQLKELGDKVPAPVKEKVDVKLQELKDAIAGGSTPTMKAAMEALNQEVMQIGQAMYNQSSGGASGPTDAGAEPTPGAGPTGSSGNDGDVIDADFTDSN

>Bradi4g33878.1 (mtHSP70-3)

MAIGSLVASRLARSGHALATAAISQAPRSRHTTSPLLSRLGAVARAFSSKPAAADVIGIDLGTTNSCVSVMDGKTPRVIENAEGARTTPSIVAKNQNGDLLIGITASRQAVTNAQNTVRGSKRLIGRTFDDPQTQKEMKMVPYKIVRGPNGDAWVEMGGQKYSPSQIGAFVLTKMKETAESFLGKTVSKAVITVPAYFNDAQRQATKDAGRIAGLEVMRIINEPTAAALSYGMNNKEGLIAVFDLGGGTFDVSILEISNGVFEVKATNGDTFLGGEDFDAALLDYLISEYQKSDNIDLSKDKAVLQRLREAAEKAKVELSSTTQTEINLPFITADASGAKHFNITLTRSKFESLVGNLIERTRIPCVNCLKDAGISAKEIDEVLLVGGMTRVPKVQDIVSQIFNKAPSKGVNPDEAVAMGAAIQGGILRGDVKELLLLDVTPLSLGIETLGGIFTRLINRNTTIPTKKSQTFSTAADNQTQVGIKVLQGEREMATDNKLLGEFQLEGIPPAPRGMPQIEVTFDIDANGIVRVSAKDKSTGKEQDITIKSSGGLSERDIENMVKEAELHSQRDQEKKSLIDLKNSADTTIYSIEKSVSEYKDKVPSEITTEVESAVSDLRAAMAGDDLDNIKQKLEAANKAVSKIGQHMQGGGGDAGGSSGGSGGDQTPEAEYQDPKEAKM

>Bradi3g57450.1 (mtHSP70-1)

MAASLLLRAARRRELYAPLGNLTTNVQSTFATNACSRWGGFARAFSAKPIGNEVIGIDLGTTNSCVSVMEGKNAKVIENSEGTRTTPSVVAFSQKGERLVGTPAKRQAVTNPQNTFFGTKRMIGRRFDDPQTQKEMKMVPYKIVKAPNGDAWVETTDGKQYSPSQIGAFVLTKMKETAESYLGKSISKAVITVPAYFNDAQRQATKDAGRIAGLDVQRIINEPTAAALSYGTNNKEGLIAVFDLGGGTFDVSILEISNGVFEVKATNGDTFLGGEDFDNTLLEFLVSEFKRTDAIDLSKDRLALQRLREAAEKAKIELSSTAQTEINLPFITADASGAKHLNITLTRSKFESLVSGLIARTRDPCKNCLKDAGITTKEVDEVLLVGGMTRVPKVQEVVSEIFGKAPSKGVNPDEAVAMGAALQGGILRGDVKELLLLDVTPLSLGIETLGGIFTRLISRNTTIPTKKSQVFSTAADNQTQVGIRVLQGEREMATDNKLLGEFDLVGIPPAPRGLPQIEVTFDIDANGIVTVSAKDKATAKEQQITIRSSGGLSESEIEKMVREAELHSQKDQERKALIDIRNTADTTIYSIEKSLGEYREKIPAEIATEIETAVADLRSEMASDDIEKIKNKMEAANKAVSKIGQHMSGGASGGGAAGGPQEGSQGGGDQAPEAEYEEVKK

>Bradi1g77637.1 (mtHSP70-2)

MAASLLLRAVRRRDLASPLGTLTANVQSTFAANVCSRWGSLARTFSAKPTGHEVIGIDLGTTNSCVSIMEGKNPKVIENSEGTRTTPSVVAFSQKGELLVGTPAKRQAVTNPQNTFFGTKRMIGRRFDDPQTQKEMKMVPYKIVKAPNGDAWVETTDGKQYSPSQIGAFVLTKMKETAESYLGKSITKAVITVPAYFNDAQRQATKDAGRIAGLDVERIINEPTAAALSYGTNNKEGLIAVFDLGGGTFDVSILEISNGVFEVKATNGDTFLGGEDFDNTLLEFLVSEFKRTEGIDLSKDRLALQRLREAAEKAKIELSSTTQTETNLPFITADSSGAKHLNITLTRSKFESLVNNLIERTRDPCKNCLKDAGITTKDVDEVLLVGGMTRVPKVQEVVSEIFGKAPSKGVNPDEAVAMGAAIQGGILRGDVKELLLLDVTPLSLGIETLGGIFTRLINRNTTIPTKKSQVFSTAADNQTQVGVRVLQGEREMASDNKLLGEFDLVGIPPAPRGMPQIEVTFDIDANGIVTVSAKDKATGKEQQITIRSSGGLSEAEIQKMVQEAELHSQKDQERKALIDIRNTADTTIYSVEKSLGEYRDKVPAEVVSEIETAISDLRTEMASDDIEKIKSKIEVANKAVSKIGQHMSGGGEASGPPSGSQGGGDQAPEAEYEEVKK

>Bradi4g43170.1 (HSP110-1)

MATARDVHVALLVCLLLFVFSGQPQPASASKSGKRCYRFRSPPVVALDIGNTNSCIAGYVADGSDAMFQLCIPSWVAFTANGTILVGDDAQDYAAVDPASAVSGFKRLIGMRWSHMYEVVQRMAKEAPYKLVEKNLYPHIKMKTGYIVGGTARNELSTDEVMAMVIGKLREAAESYMSCSVRHAVFTVPRHYYDSPWRQTEFAGHIAGVRVARMLDEPIAAAVAHGLHRRLRNEGVALVLHVGGATTEASLMVLDDGVFDFLGGRHDAFLGGDDFDRRVVDYFTALMKRKHGRDISNDTQALAKLRTACEHAKKALSTRRQAEVVVESLGLAETLTRAEFEELNGDLFGEVVKLVHRAMVGADRELDGRSIWDAVDEVLLVGGSAVIPEIQRLVRDYFGGRKKVAVHAGVKPDEVVTLGGALLTRADAGGYPCMGVDGRRQRGYHSDWCDNRRW

>Bradi1g69700.1 (HSP110-2)

MAEQFYTVASDSETTGEEKAQQTFPDVAIGIDIGTSKCSVAVWNGHQVELLKNTRSQKGMRSYVMFKDDTLSAGVTGGATKEHAHEERDVLSGSAIFNMKRLIGRMDTDEVVQASKTLPFLVQTLGIGVRPFIAALVNNMWRSTTPEEVLAIFLLELKALVEMHYKHPVRNTILTIPVAFSRFQQTRIERACAMAGLHVLRLMPEPTAVALLYAQQQQQLLHDNMGSGIEKIALIFNMGAGYCDVAVSATAGGVSQIRALSGCTVGGEDILQNVMRHLHPNFDSLFAGHTMDRIKSMGLLRIATQDAVHKLTTQESIEINVDLGGGQKVSKVLGRAEFEQVNKMIFEECERVIKQCLHDAKLAPEDINDVILVGGCSRIPKIRSLVLGLCKKADSYASIDVLEAAVSGAALEGAIASGVTDPSGSLDLLTIQATPMNLGIRADGDTFAAIIPRNTAVPARRDMLFTTTHDNQTEALVAVYEGEGKQAEENHLLGYFKITGIPAAPKGAVEINVCMDIDAANVLRVFAGVVKPQGPATPPFIEVRMPTLDDGHGWCGQALAKMYGGNLDLAVLPKKLQP

>Bradi3g53100.1 (HSP110-3)

MAPRLLLLALVVAAVAVPPASAAVASIDLGSEWLKVAAVHLAPGRAPIAVAINEMSKRKSPALAALADGNRLAGEEAAGIAARHPSKVFARMRDLLGKPFPYVQSLAQSLFLPYDFVQDARGAAAVRADDGQVYSVEEIVAMVLHYASGIADAHVGAPVRDAVVAVPPYFGQAERRSLTQAAQLAGFNVLALINEHAGAALQYGIDKDFSNGSRHVIFYDMGSGSTYAALVYYSSYNAKEFGKTVSVNQFQVKDVRWNSKLGGIEMEMRLVNYFADQFNKQLGNGDDIRQSPKAMAKLKKQVKRTKEILSANTAAPISVESLYNDIDFRSTITREKFEELCEDLWEQALTPIKEVLVQSGMKIDDIYAVELIGGATRVPKLQAKLQEFLGRSELDKHLDADEAIVLGASLHAANLSDGIKLNRKLGMIDGSPYGFVFEINGPDYVKDESTDQVLVPRMKKMPIKLYRSVKHTKDFDVSLSYDKASELPPGVSSHKFAEYSISGLADASEKYGSRNLSAPIKANLHFSLSRSGIISLDRAEAVIEITEWAEIPKKNLTLESNATDQTLSSESGTSDSTADSKENPSSGSDANNLSNTNDEGNVRDAITEKVLKKRTFRVPLKVVEKTAGAGSILSKELYSEAKNRLEVLDKKDAERRRTAELKNNLESYIYSMKEKLEENTDLLAVSTEQERESFAEKLSEVQDWLYMDEEAQANEFQERLDQLKAIGDPILFRLSELKARPAACGSARLYLTELQKIVKNWETNKPWLPKKRVDEVVSEADKIRTWLDEKEALQKSTTLYSTPAFTSEEVYQKVLDLQDKVSSVNRIPKPKPKIEKKPPTEEESANKEKTASSESTSSESESTDTSSESDAPEKTDDSEPEAHDEL

>Bradi1g32770.1 (HSP110-4)

MSVVGFDVGNDTLVAAAARQRGIDVLLNAESKRESPAAVAFAHNARLLGPHAAGAASSHAPFSSPKRLLLLAARPALVPRDLPRLPFPVHVPADGDALVHVDHIGRRIALSPTHLLAMLLAYLKQLAEADLEAPVADCVISVPCYFTQAHRRAYLDAAAVAGLTPLRLMHDLAATALGYGLYRSDLGVAGSPTFVAFVDVGHSDTQATVVAFDPSGMKVLSHGFDADLGGRDFDEVLFEHFAEEFRDRYKIDVVGNVKASMRLRAACEKAKKVLSANAEAVVNIECLMEEKDVRGMIRREEFEKLCSQLLERVVEPCKRAMADSGVGLEKLQSVELVGSGSRVPAIARVLAEFFRREPSRTINVSECVARGCALQCAMLSPTLRVREYEVQDAIPASIGFCTNEGPISTLSSNALFRRGQPLPSVKIITLHRNSGFNLDVYYLDENELPPGTSTKIGSFQIGPFHAHTEKSKVKVKIRLNLHGLISVESAVLIEDDQRDTNSSDSMEVDHNNDVGDKSRNERPIQRQDLQIIGSIYGAMSKQELLEAQEQEYQLAYQDKLMERTKERKNALESYVYDIRNKLSERYRSFATDSEREQISVNLQQTEEWLYEEGDDETEEVYSSKLEELKKLVDPIENRCKDDEVRTQTTRELLKCIVDHRMAAKSLSAPEREAVDNECAKVEQWLREGLKLQESLPKDVDPVLWSPEIKRKEEELDMLYRNIVTSKARGHSSEDGC

>Bradi2g33676.1 (HSP110-5)

MSVVGFDLGNESCIVGVARQRGIDVVLNEESKRETPAIVCFGDKQRFIGTAGAANSTMNPKNSISQIKRLLGRKFTDPEVQHDIQSFPFRVSEGPDGFPLVHARYLGEERTFTPTQLLAMVLSNLKGIAEGNLNTAVSDCCIGIPVYFTDLQRRAVLDAATIAGLRPLRLFHETTATALAYGIYKTDLPENDQLNVAFVDIGHASMQVSIVGYKKGQLKMLSHAYDRSLGGRDFDEALFKHFAAKFKEEYKIDVYQNARACIRLRVACEKLKKMLSANPEAPMNIECLMDEKDVRGFIKRDEFEQISGPVLERVKGPLDKALAEAGLTTESVHFVEVVGSGSRVPAIMKIITEFFGKEPRRTMNASECVARGCALQCAILSPTFKVREFQVNEGFPFSVALSWKPDSPGNEAQQTVVFPKGNPIPSIKALTFYRSNTFAVDVLNVDTDDMQITQKISTYTIGPFQSSKGDKAKLKVKVRLNIHGIVSLESATMLEEEEVEVPVTSEVPKDATKMDTDDATGTDVNMQESKGATDTAEGAAENGAQDSEEKSVPMDTDTKVEPSKKKVKKTNVPVTELVYGAMSAAELEKAVEKEYEMALQDRVMEETKDKKNSVEAYVYDMRNKLSEKYNDFVMSEDMEGLRVKLQEVEDWLYEDGEDETKGVYVAKLEELKKVGDPIEMRYKEWAERGQALEQLVYCIRSFREAALSSDQKFDHIDIAEKQKVVNECSNAETWLLEKKQEQDALPKHINPVLLVSDIKKKAEALDRFCKPIMTKPKPAPKPQTPPPAENPAPEAQTPEQQSSGASEADEPANEGASQDQPAAEQMETDRAEPSSA

>Bradi1g75681.2 (HSP110-6)

MATRLLYQLGVLLLLAIAAAWTSGAAAFGLPRVSSGYCVWTSPFNTVNATAIHIGNTKSCISGYGGRLDPYTMYQFCIPSWVAFTANSTLFGEAAMNHAGVSPGTAVSGFKRLLGVRVFAGILMGELKKTAEAWLGREIMYAVVTVPADFNDAQRSHVTAAAELRGGFHAAKAVDEEVAAAAAYRLHEKRGDGKAILVFHLGGRTCHATKFRFHNGTARLLVERHDAYLGGDDFTCRIVDYFVKLIKEKHHRDISEDEGALRKLRADSEVLKKS

>LOC_Os05g38530 (cHsp70-6)

MSKGEGPAIGIDLGTTYSCVGVWQHDRVEIIANDQGNRTTPSYVAFTDTERLIGDAAKNQVAMNPTNTVFDAKRLIGRRFSDPSVQSDMKLWPFKVVPGPGDKPMIVVQYKGEEKQFAAEEISSMVLIKMREIAEAYLGSSIKNAVVTVPAYFNDSQRQATKDAGVIAGLNVMRIINEPTAAAIAYGLDKKATSSGEKNVLIFDLGGGTFDVSLLTIEEGIFEVKATAGDTHLGGEDFDNRMVNHFVQEFKRKNKKDISGNPRALRRLRTACERAKRTLSSTAQTTIEIDSLYEGIDFYTTITRARFEELNMDLFRKCMEPVEKCLRDAKMDKSSVHDVVLVGGSTRIPKVQQLLQDFFNGKELCKSINPDEAVAYGAAVQAAILSGEGNEKVQDLLLLDVTPLSLGLETAGGVMTVLIPRNTTIPTKKEQVFSTYSDNQPGVLIQVYEGERARTKDNNLLGKFELSGIPPAPRGVPQINVCFDIDANGILNVSAEDKTTGQKNKITITNDKGRLSKEEIEKMVQEAEKYKAEDEEHKKKVDAKNALENYAYNMRNTIKDEKIASKLAADDKKRIEDAIDGAISWLDTNQLAEADEFEDKMKELEGICNPIIAKMYQGPGADMAGGMDEDAPAGGSGAGPKIEEVD*

>LOC_Os01g62290 (cHsp70-1)

MAKGEGPAIGIDLGTTYSCVGVWQHDRVEIIANDQGNRTTPSYVAFTDSERLIGDAAKNQVAMNPINTVFDAKRLIGRRFSDPSVQSDMKLWPFKVIPGPGDKPMIVVQYKGEEKQFSAEEISSMVLIKMREIAEAYLGSNIKNAVVTVPAYFNDSQRQATKDAGVIAGLNVMRIINEPTAAAIAYGLDKKATSSGEKNVLIFDLGGGTFDVSLLTIEEGIFEVKATAGDTHLGGEDFDNRMVNHFVLEFKRKNKKDISGNPRALRRLRTACERAKRTLSSTAQTTIEIDSLYEGIDFYSTITRARFEELNMDLFRKCMEPVEKCLRDAKMDKSSVHDVVLVGGSTRIPKVQQLLQDFFNGKELCKSINPDEAVAYGAAVQAAILSGEGNEKVQDLLLLDVTPLSLGLETAGGVMTVLIPRNTTIPTKKEQVFSTYSDNQPGVLIQVYEGERARTKDNNLLGKFELSGIPPAPRGVPQITVCFDIDANGILNVSAEDKTTGQKNKITITNDKGRLSKEEIEKMVQEAEKYKAEDEEHKKKVDAKNALENYAYNMRNTIKDDKIASKLSADDKKKIEDAIDGAINWLDSNQLAEADEFEDKMKELESICNPIIAKMYQGAGADMGGAAGMDEDAPAGGSGAGPKIEEVD*

>LOC_Os11g47760 (cHsp70-7)

MAGKGEGPAIGIDLGTTYSCVGVWQHDRVEIIANDQGNRTTPSYVGFTDSERLIGDAAKNQVAMNPINTVFDAKRLIGRRFSDASVQSDIKLWPFKVIAGPGDKPMIVVQYKGEEKQFAAEEISSMVLIKMREIAEAYLGTTIKNAVVTVPAYFNDSQRQATKDAGVIAGLNVMRIINEPTAAAIAYGLDKKATSVGEKNVLIFDLGGGTFDVSLLTIEEGIFEVKATAGDTHLGGEDFDNRMVNHFVQEFKRKNKKDITGNPRALRRLRTACERAKRTLSSTAQTTIEIDSLYEGIDFYSTITRARFEELNMDLFRKCMEPVEKCLRDAKMDKSSVHDVVLVGGSTRIPRVQQLLQDFFNGKELCKNINPDEAVAYGAAVQAAILSGEGNEKVQDLLLLDVTPLSLGLETAGGVMTVLIPRNTTIPTKKEQVFSTYSDNQPGVLIQVYEGERTRTRDNNLLGKFELSGIPPAPRGVPQITVCFDIDANGILNVSAEDKTTGQKNKITITNDKGRLSKEEIEKMVQEAEKYKSEDEEHKKKVESKNALENYAYNMRNTIKDEKIASKLPAADKKKIEDAIDQAIQWLDGNQLAEADEFDDKMKELEGICNPIIAKMYQGAGADMAGGMDEDDAPPAGGSGAGPKIEEVD*

>LOC_Os12g38180 (uHsp70-2)

MSCVSSMSLLLLPLPMVLTRRLPVLVRRTSSSSTLVVVPLMSLFSPLRREVKATAGDTHLGGEDFDNRMVKHFVQEFKRKSKKDITGNPRPVGRLRTACEWAKRTLSPPLPRPPTIEIDSLYEGIDFYSNITCARFEELTMDLFRKCMRGYQDGQRAACTMFILVGGSTRIPRVQQLLQDFFNGKELCKNINPDEAVAYGAAVQAPILVWRRPEE*

>LOC_Os03g16920 (cHsp70-4)

MAGNKGEGPAIGIDLGTTYSCVGVWQHDRVEIIANDQGNRTTPSYVAFTDTERLIGDAAKNQVAMNPTNTVFDAKRLIGRRFSDPSVQADMKMWPFKVVPGPADKPMIVVTYKGEEKKFSAEEISSMVLTKMKEIAEAFLSTTIKNAVITVPAYFNDSQRQATKDAGVISGLNVMRIINEPTAAAIAYGLDKKAASTGEKNVLIFDLGGGTFDVSILTIEEGIFEVKATAGDTHLGGEDFDNRMVNHFVQEFKRKHKKDITGNPRALRRLRTACERAKRTLSSTAQTTIEIDSLYEGIDFYATITRARFEELNMDLFRRCMEPVEKCLRDAKMDKAQIHDVVLVGGSTRIPKVQQLLQDFFNGKELCKSINPDEAVAYGAAVQAAILSGEGNQRVQDLLLLDVTPLSLGLETAGGVMTVLIPRNTTIPTKKEQVFSTYSDNQPGVLIQVYEGERTRTKDNNLLGKFELTGIPPAPRGVPQINVTFDIDANGILNVSAEDKTTGKKNKITITNDKGRLSKEEIERMVQEAEKYKAEDEQVRHKVEARNALENYAYNMRNTVRDEKIASKLPADDKKKIEDAIEDAIKWLDGNQLAEADEFEDKMKELESLCNPIISKMYQGGAGGPAGMDEDAPNGSAGTGGGSGAGPKIEEVD*

>LOC_Os03g60620 (cHsp70-5)

MAPTKGEGPAIGIDLGTTYSCVGVWQHDRVEIIANDQGNRTTPSYVGFTDTERLIGDAAKNQVAMNPINTVFDAKRLIGRRFSDASVQSDIKMWPYKVIPGPGDKPMIVVQYKGEEKQFSAEEISSMVLIKMREIAEAYLGSTVKNAVVTVPAYFNDSQRQATKDAGVIAGLNVMRIINEPTAAAIAYGLDKKATSVGEKNVLIFDLGGGTFDVSLLTIEEGIFEVKATAGDTHLGGEDFDNRLVNHFVQEFKRKHKKDISGNPRALRRLRTSCERAKRTLSSTAQTTIEIDSLFEGVDFYSTITRARFEELNMDLFRKCMEPVEKCLRDAKMDKSTVHDVVLVGGSTRIPRVQQLLQDFFNGKELCKSINPDEAVAYGAAVQAAILSGEGNEKVQDLLLLDVTPLSLGLETAGGVMTVLIPRNTTIPTKKEQVFSTYSDNQPGVLIQVYEGERTRTRDNNLLGKFELSGIPPAPRGVPQITVCFDIDANGILNVSAEDKTTGQKNKITITNDKGRLSKEEIEKMVQDAEKYKSEDEEHKKKVDAKNSLENYAYNMRNTIQDEKIASKLPADDKKKIEDAVEQAIQWLDNNQLAEVEEFEDKMKELEGLCNPIIAKMYQGAGADMGGGMDDDAPAAGGSGAGPKIEEVD*

>LOC_Os03g16860 (cHsp70-2)

MAGKGDGPAIGIDLGTTYSCVGVWQHDRVEIIANDQGNRTTPSYVAFTDSERLIGDAAKNQVAMNPINTVFDAKRLIGRRFSDTSVQSDAKLWPFKVLPGPGDKPMIGVQYKGEEKQFSAEEISSMVLNKMKETAEAYLGSTVKNAVVTVPAYFNDSQRQATKDAGVISGLNVMRIINEPTAAAIAYGLDKKSSSVGEKNVLIFDLGGGTFDVSLLTIEEGIFEVKATAGDTHLGGEDFDNRMVNHFVQEFKRKNKKDITGNPRALRRLRTACERAKRTLSSTAQTTIEIDSLYEGIDFYTTITRARFEELNMDLFRKCMEPVEKCLRDAKMDKSTVHDVVLVGGSTRIPRVQQLLQDFFNGKELCKSINPDEAVAYGAAVQAAILTGEGNEKVQDLLLLDVTPLSQGLETAGGVMTVLIPRNTTIPTKKEQVFSTYSDNQPGVLIQVYEGERTRTKDNNLLGKFELSGIPPAPRGVPQITVCFDIDANGILNVSAEDKTTGQKNKITITNDKGRLSKEDIEKMVQEAEKYKAEDEEHKKKVDAKNSLENYAYNMRNTIKDDKIASKLPEADKKKIEDAIDGAISWLDSNQLAEAEEFEDKMKELEGVCNPIIAKMYQGAGADMAGGMDEDAPAAAGGSSGPGPKIEEVD*

>LOC_Os01g49430 (uHsp70-1)

MAETGDQHGPVIGIDLGTACSCVAVWQNGRAEIVTNEHGGRATPSYAAFTDTERLVGDAAKSQASRNPTNTVFATKRLMGRRFSDASVQDGLKLWPFKVVPGRGDKPMVAASYKGKQKLLAAEEVASMLLSKMKAEAEAYIGGPVKNAVVTVPASFDVLQRRATKHACAVAGLDVLGVIHGPAAAAVAFGIHEIAGDKNVLVFDLGGGHTSVSLLAVASGKIAVRATAGDPHLGGEDFNGRMVEHFVAQFKAEHKKDVGRNARAILRLRAACEQAKRTLSSASWAAIELERLHDGADFYSTITRDQFDELNLDLFCKCLDPIKKCLTGAKMDRSSVDDVVFVGGSTRIPRVRRLIQDLFDGKELRKDISSDEAAACGAATMASLGSDDSLVDLFLFDATPHSLGVAAAGGAMAVMIPKNTPIPVMARENTISIQPNHKKGHKQVRTLSSARSKLPGAHSKAKPGVKLSVSVCFSIDADGVLTVSARDKVNGHKNQMRVMEQSQLSKEEIERMTMEAKEYMAADEEKERIKAKNLLEEFL*

>LOC_Os03g16880 (cHsp70-3)

MGFLPGDDDGPAVGIDLGTTYSCVAVWRRGRVEVIPNDQGNLTTPSCVAFTDTWRLIGDAALNQAAMNPLNTIFDAKRLIGRRFSDVSVQGDIKLWPFKVTSGKDDRPMIVVQYRGEEKEFSAEEISAMVLFKMKETAEAYLDKTVEKAVITVPVYFNDSQRQATMDAGAVAGLDVLRIINEPTAAAVAYGLDKVVGSSDKKKRVLIFDLGGGTLDVSVLNIDPGVDIDIGIFEVKATAGDTHLGGEDFNGRMVKHLVREFLRKYKRPEIRGDQRALRRLRTACEKAKRMLSSTAQTTIEIDSLHGGVDFHATVTRAKFEELNMDLFLECMDTVKQCLRDAGTDKGTVDDVVLVGGSTRIPKVRSLLQDLFDGKALCRSINPDEAVAHGAAVKAALLNGDANEKDLRDVVLLDVTPLSLGIEVFGGGMSVLIPRNTTIPVSRQKMYTTHYDNETAVLINVNEGEGRRTKDNNPLGKFVLTGIPPAPRGVPQINVTFSIDYNGIMNVSAEDKTTGRSNSITIKSDKGRLSKEEIERMVKKAEKYKAEDEEEMKKAEGYVAP*

>LOC_Os11g08460 (cHsp70-10)

MASAPGDGKQGGGGGGPAVGIDLGTTYSCVAVWRHDRGEVIANDQGNRLTPSCVAFTADDDDSFVGDAAFNQSALNPTNTIFEVKRLIGRRFSDDSVQKDIKLWPFKVVAGQEDRPMIVVRHEGEERQFMPEEISSMVLAKMRETAEVYLGKTVTNAVITVPVYFNNAQRQATMDAGAIAGLNVMRIINEPTAAALAYGLEKMPVSNKGRMVLVFDLGGGTFDISLLNIDPGVNIDMGLFEVKATAGDTHLGGADFDNELVKHSLREFNRKHGSMDIESNQKALRRLRTACERAKRMLSSTMQTTIEVDSLHQGIDFRVTLTRSRFEELNKDLFSKCMEAMENCLRDAKVDKWSVDDVVLVGGSTRIPKVQKMLSEFFDGKELCRSINPDEAVAYGAAIQASILCGGTDDKRLVDMLLREVTPLSLGVETEDNCTMSVVIPRNTAIPTKKVKNFTTLYDNQINVSFPVYEGESANTKDNNLLGEFTLYGIPPAPKRVPSIDVTFDIDANGVLNVSAEHKVTGQKNSITITNRSGRLNKEEIDRMALEAERHKMKRIKQNEVV*

>LOC_Os11g08440 (cHsp70-8)

MAASNTTKKGGGGGGPAIGIDLGTTYSCVAVRRRYRSEAITNDQGNRITPSCVAFTAADRFVGDAAENQAALNPTNTIFEVKRLIGRRFSDKSVQEDIKLWPFKVIAGRDDRPTIVVRHEGKEKQFVPEEISAMVLSKLRDAAVAYLGEPVTDAVITVPVYFNNAQREATLDAATIAGLNVMRIINEPSAAALAYGLDKMPPASGGAGRMVLIFDLGGGTLDVSLLNIGRPGNNNSSDSGSFEFEVKAVAGDTHLGGADFNNAIVKHCINEFIRKHGVAAEGIWSNQKAIRRLRTACERAKRMLSFTTLASIEVDSLHDGIDFCGKMSRSRFEELNKELFGKCVKAVKKCLEDAKMDKNAVDDVVLVGGSSRIPKLQSMIHDFFDEKKLRRNVNPDEAVAYGAAIQASVLNGDADEADDKKQVMILRDITPLSLGIEVGLDHTMSVVIPRNTFIPTKNVRRYSTIFDNQIAVSINVFEGESASILRNNLLGKFVLSGILPAPRGVPQIDVTFEFDANGVLHVSAKDMGTGSKNNIAITNHSGRLKKEDVERMAREARSYNRTRSSLAITSGNLVIFQ*

>LOC_Os11g08445 (cHsp70-9)

MVLEEDGSFLVPFDPNASDCGDQTMAASNTSKKGGGGDGGGGGGPAIGIDLGTTYSCAAVRRHNRSEVITNDQGNRITPSCVAFTADDRFVGDAAENQAALNPTNTIFEAKRLIGRRFSSKSVQEDIKLWPFKVVAGPDDRPTIVVQHEGKEMQFVPEEISAMVLSKLRDAAVAYLGEPVTDAVITVPVYFNNAQREATLDAAAIAGLNVMRIINEPSAAAIAYGLDKMPPPPASGGGAAVRTVLIFDLGGGTLDVSLLNIGRPGNNSNSGDNGSSFEFEVKAVAGDTHLGGADFDNAMVNHCINEFIRKHDVAEEGIRSNQKAIRRLRSACERAKRLLSFTAQTSIEVDSLHDGVDFCAKMSRSRFEELNKELFGRCVKAVEKCLEDAKMDKGDVHDVVLVGGSSRIPKLQSMLHDFFQEKKLRHSVNPDEAVAYGAAIQASILNGDADDADDKKKAMILRDITPFSLGVEIYDENDHTMSVVIPRNTFIPAKNTQRYTTHRDMQTSVSIKVFEGESASTKNNYLLGEFVLSGITPAPAGVARIDETLEIDANGVLHVSAEDMGTGRKNSITITNHSGRLKKEDVERMSREARSYNRKRKRTRSSLQCCSCSNSLQPAWNYFGARRCSCLISVAAPSMFPSLTSIRGSTLTWAGSSR*

>LOC_Os11g08470 (cHsp70-11)

MQFVPEEVSAMVLSKLRDAAVAYLSEPVTDAVITVPVYFNNAQREATLDAAAIAGLNVMRIINEPSAAAIAYGLDKMPPPASGADAAGRTVLIFDLGGGTLDVSLLNIARPGNNSSSNSDNGSFEFEVKAVAGDTHLGGADFDNAMVKHCINEFIRKNDVAEDGVWSNQKAIRRLRSACERAKRLLSFTAQTSIEVDSLHDGVDFCAKMSRSRFEELNKELFGKCVKAVEKCLEDAKMDKSAVHDVVLVGGSSRIPKVQSMLHDFFQEKKLRHSVNPDEAVAYGAAIQASILNGDSDNADADADDKKRVMILRDITPLSLGVEINFDHTMSVVIPRNTFIPTKNTRRYTTLYDNQIRVSFPVFEGESASTLDNNLLGKFVLSGVLPAPRGVPQIDVTFDFDTNGVLHVFAEDMGTGSKNNNTITNHSGRLKKEDVERMSREARSYNRKRKRTRSSLQMNSGNLVILE*

>LOC_Os02g02410 (BiP1)

MDRVRGCAFLLGVLLAGSLFAFSVAKEETKKLGTVIGIDLGTTYSCVGVYKNGHVEIIANDQGNRITPSWVAFTDSERLIGEAAKNQAAVNPERTIFDVKRLIGRKFEDKEVQRDMKLVPYKIVNKDGKPYIQVKIKDGENKVFSPEEVSAMILGKMKETAEAYLGKKINDAVVTVPAYFNDAQRQATKDAGVIAGLNVARIINEPTAAAIAYGLDKKGGEKNILVFDLGGGTFDVSILTIDNGVFEVLATNGDTHLGGEDFDQRIMEYFIKLIKKKYSKDISKDNRALGKLRREAERAKRALSNQHQVRVEIESLFDGTDFSEPLTRARFEELNNDLFRKTMGPVKKAMDDAGLEKSQIHEIVLVGGSTRIPKVQQLLRDYFEGKEPNKGVNPDEAVAYGAAVQGSILSGEGGDETKDILLLDVAPLTLGIETVGGVMTKLIPRNTVIPTKKSQVFTTYQDQQTTVSIQVFEGERSMTKDCRLLGKFDLSGIPAAPRGTPQIEVTFEVDANGILNVKAEDKGTGKSEKITITNEKGRLSQEEIDRMVREAEEFAEEDKKVKERIDARNQLETYVYNMKNTVGDKDKLADKLESEEKEKVEEALKEALEWLDENQTAEKEEYEEKLKEVEAVCNPIISAVYQRTGGAPGGGADGEGGVDDEHDEL*

>LOC_Os03g50250 (BiP2)

MARDKQSALIVAAFVLLCSGCLCGVADGAKGGRKTKGPVIGIDLGTTYSCVGVYRNGHVDIVANDQGNRITPSWVAFTDDERLVGEAAKNQAALNPDRTIFDIKRLIGRRFDDEEVQRDVKYLPYKVVDKGGKPYVEVRVKAGEVKVFSPEEISAMILAKMKETAESYLGQRVTDAVVTVPAYFNDAQRQATKDAGTIAGLNVPRIINEPTAAAIAYGLDRKGAGEMTNVLVYDLGGGTFDVSVLSLDHGVFEVLATSGDTHLGGEDFDRRVMDHFIRLVKRQHGRDIGGDGRALGKLRRECERAKRALSRQHQVRVEIEALFVGVDFSETLTRAKFEELNMDLFKKTLGPVRKAIADAKLKKSDIDEIVLVGGSTRIPKVQELLKEMFDGKEPTKGINPDEAVAYGAAVQGSIISGEGGAETKDILLLDVTPLTLGIETAGGVMTKLIPRNTRIPVKKSQVFTTYEDHQTTVSIKVFEGERSLTKDCRELGRFDLSGIAPAPRGVPQIEVTFEVDENGILHVTASDKAAGRSKSITITNDKGRLSQEEIDRMVREAEEFAEEDRRVRERVDARNRLENYVYRMRSAVRDGGMAGKIGDDDRERMESALTEALEWLEDNDGGARTAEKEDYEEKLKEVEQVCGPIIKQVYKKSGDASAGAGDDDDVNEL*

>LOC_Os01g33360 (BiP6)

MARMAAAATLLLAVSGLASGLVVPADERCHSTDNAVLGIDIGATYSCVAVYCKGRVEIIPNDQGSRLTPSWVAFTDGGGRLVVGEAAKEQAVGSPGRAVHDFMRLLGKKLGDDDVQREMTRLPYAVVDMEGKPHVLVEAADGDVRVLSPEEIAAAVLAKMKKTAEAHLGRTVSSAVVAVPVYFNDAQRRAISDAGDIAGLDVMRIVSEPIAAAVAYGLDNVRSNGKRVVVFDLGGENLDVTALVADDGFFDVLATNGDGAQHQVRVEIEALLDGGVDLSETLTRAQFEELNDDLFARTMAPLRKTMADAGLEKGDINEIIHVGGSTRIPKVQQLIRDYFDGKKEIVKVNNPDETVAYGAAVIGRHVAGDDDDKPTMLGPLDLPSFLSDTISIETAGGAVTPMIPRRSRLPAERTHVFTTYLGRQTAVAINVFQGEGSTAKDNTLFGRLVLTGIPPASVWNWGWRWRPIQVTVKVDELGDIHVEATDKGGSGKSERLSIVSGEGHEHGRLSKEEIDRMIREVAEDLVEEERIVKERVDALNMLETYIVKNTAVTGGETDCEAKARAASEWLDGNPAAEKEDYEEKLKELEDACGPVHGGRARDIWTWPR*

>LOC_Os05g30480 (BiP3)

MARGATWTRRLHLHGLFLAVLLLLTLPAGSTAAAGGGGGTVIGIDLGTTYSCVGVYRNGHVEIIANDQGNRITPSWVAFTGGGERLIGEAAKNQAAANPGRTVYDAKRLIGRRFADAEVQRDMRLLPFAVVDKGGKPHVRVEVRGGDVRLLSPEEVSAMVLARMKETAEAYLGEEVTRAVVTVPAYFNDAQRQATKDAATIAGLAVERILNEPTAAALAYGVGKEGAGGKNVLVFDLGGGTFDVSVLAIDGGVYEVLATNGDTHLGGEDFDQRVMEHFVELVRRKHGRDIAGDARALGKLRRECERAKRALSIQHQVRVEVESLFDGVDLSEPLSRARFEELNNDLFRKTMAPVRKAMADARLSNADIDEIVLVGGSTRIPKVRQLLRDYFGGKQPNQGVNPDEAVAYGAAIQANIVGGDTDNKTRDMVVLDVTPLTLGLETAGGVMATLIPRNTPVPTKRAQLFSTYKDKQTTVTVKVFEGERSMTRDNRLLGRFDLAGIAPAPRGAPQIEVAFEVDADGILSVSAADRATGRSERITISGDDRKTSREEIDRMLGEAEEFADEDRRHRERAGARNSLEAYVYGVKNAVVGGEMAGAMDGGEKEKVEAAVMEAYEWLDGNQDVGKEEYEEKLRELEDVCNPVMSAVYQRSGGSRRDGDGGGDDDHDEL*

>LOC_Os08g09770 (BiP5)

MARPRRASTMQLGLFLAALLLLTPSPAGSVAAAKGGGAKSGGGGTVIGIDLGTTYSCVGVYRNGHVEIIANDQGNRITPSWVAFTDGGERLIGEAAKNQAAANPERTIYDAKRLIGRQFSDAEVQRDMKLLPFAVVDRNGKPHVRVEVKDGDVRVFSPEEVSAMVLTRMKETAEAYLGEKVTRAVVTVPAYFNDAQRQATKDAGVIAGLTVDRIINEPTAAAIAYGIDKKGAEKNVLVFDLGGGTFDVSILAIDNGVFEVLATNGDTHLGGEDFDQRLMDHFVKVIRRKHGRDITGDARALGKLRRECERAKRALSNQHQVRVEVESLFDGVDLSEPLSRARFEELNSDLFKKTMVPVRKAMADARLSKGDIDEIVLVGGSTRIPKVQQLLKDYFGGKEPNRGVNPDEAVAYGAAVQASIISGHVDENTESMILLDVAPLTLGLETAGGVMAKLIPRNTVVPTKKTQVFTTYKDKQTTVTIQVFEGERSMTRDNRLLGRFDLAGIAPAPRGAPQIEVTFEVDANGILSVLAADKATGRSEKITISGDDRKISQEEIDRMVREAEEFAEEDRRHREQVDARNSLEAYVYNIKNTLGGKMADAMEGEEKDKVEEAVREAYEWLDGNPDAGKEEYEEKLRELEDVCNPVMSAVYQRSGGGGGGAPEDGNVDDEDDHDEL*

>LOC_Os05g35400 (BiP4)

MARPRRASTATTMQLGLLLAALLLFTSSLAGSVAAAAPPPPAGAKGGGAKSGGGGGTVIGIDLGTTYSCVGVYRNDRVEIIANDQGNRITPSWVAFTDGGERLIGEAAKNQAAANPERTIYDAKRLIGRQFSDAEVQRDMKLLPFAVVDRNGKPHVRVEVKDGDVRVFSPEEVSAMVLTRMKETAEAYLGEKVTRAVVTVPAYFNDAQRQATKDAGVIAGLTVDRIINEPTAAAIAYGIDKKGAEKNVLVFDLGGGTFDVSILAIDNGVFEVLATNGDTHLGGEDFDQRLMDHFVKVIRRKHGRDIAGDARALGKLRRECERAKRALSNQHQVRVEIESLFDGVDFSEPLSRARFEELNGDLFKKTMVPVRKAMADAGLGKGDIDEIVLVGGSTRIPKVQQLLKDYFGGKEPNRGVNPDEAVAYGAAVQASIISGHVDENTESMILLDVAPLTLGLETAGGVMTKLIPRNTVVPTKKTQVFTTYKDRQTTVTIQVFEGERSMTRDNRLLGKFDLTGIAPAPRGAPQIAVTFEVDANGILSVLAADKATGRSEKITISGDDRKISQEEIDRMVREAEEFADEDRRHREQVDARNSLEAYVYNVKSTLGGKMADAMEGEEKEKVEEAVREAHEWLDGNPDAGKEEYEEKLRELEDVCNPVMSAVYQRSGGGGGAPEDGNVDDEDDHDEL*

>LOC_Os05g23740 (cpHsp70-1)

MATTTFPTSTPFFAHHGRRRPSPSVSVRTAAAVYGRGGGRRWRPLRVACEKVVGIDLGTTNSAVAAMEGGKPTIVTNAEGARTTPSVVAYTKSGDRLVGQIAKRQAVVNPENTFFSVKRFIGRKMNEVDEESKQVSYRVIRDDNGNVKLDCPAIGKQFAAEEISAQVLRKLVDDASKFLNDKVTKAVITVPAYFNDSQRTATKDAGRIAGLEVLRIINEPTAASLAYGFEKKNNETILVFDLGGGTFDVSVLEVGDGVFEVLSTSGDTHLGGDDFDKRVVDWLAGNFKNDEGIDLLKDKQALQRLTEAAEKAKMELSSLTQTNISLPFITATADGPKHIETTLTRAKFEELCSDLLDRLRTPVDNALRDAKLSFKDIDEVILVGGSTRIPAVQDLVKKMTGKDPNVTVNPDEVVALGAAVQAGVLSGDVSDIVLLDVTPLSLGLETLGGVMTKIIPRNTTLPTSKSEVFSTAADGQTSVEINVLQGEREFVRDNKSLGSFRLDGIPPAPRGVPQIEVKFDIDANGILSVSAVDKGTGKKQDITITGASTLPKDEVEKMVEEAEKFAKEDKEKRDAIDTKNQAESVIYQTEKQLKELGDKVPGDVKGKVEAKLTELKDAVAGGSTQTMKDALAALNQEVMQLGQALYSQQGAPGAGPTPGADAAAGSAGPSEKPGGEEGDVIDADFTDSQ*

>LOC_Os12g14070 (cpHsp70-2)

MASFTSQLGAMACGAAPSTSPLAARRSGQLFVGRKPAAASVQMRVPRAGRARGVAMRVACEKVVGIDLGTTNSAVAAMEGGKPTVITNAEGQRTTPSVVAYTKGGERLVGQIAKRQAVVNPENTFFSVKRFIGRKMAEVDDEAKQVSYHVVRDDNGNVKLDCPAIGKQFAAEEISAQVLRKLVDDASKFLNDKITKAVVTVPAYFNDSQRTATKDAGRIAGLEVLRIINEPTAASLAYGFEKKNNETILVFDLGGGTFDVSVLEVGDGVFEVLSTSGDTHLGGDDFDKKVVDWLASNFKKDEGIDLLKDKQALQRLTEAAEKAKMELSTLSQTNISLPFITATADGPKHIETTLSRAKFEELCSDLIDRLKTPVTNALRDAKLSVDNLDEVILVGGSTRIPSVQELVKKITGKDPNVTVNPDEVVSLGAAVQGGVLAGDVKDVVLLDVTPLSLGLETLGGVMTKIIPRNTTLPTSKSEVFSTAADGQTSVEINVLQGEREFVRDNKSLGSFRLDGIPPAPRGVPQIEVKFDIDANGILSVAAIDKGTGKKQDITITGASTLPKDEVERMVEEADKFAQEDKEKRDAIDTKNQADSVVYQTEKQLKELGDKVPAPVKEKVDAKLNELKEAIAGGSTQSMKDAMAALNEEVMQIGQAMYNQQPNAGAAGPTPGADAGPTSSGGKGPNDGDVIDADFTDSN*

>LOC_Os09g31486 (mtHsp70-3)

MAIGSLIASRLARSTGHALASAASQAPMARHAAASPLLSRLGSVARAFSSKPAAADVIGIDLGTTNSCVSVMEGKTPRVIENAEGARTTPSIVAKNQNGDLLVGITASRQAVTNAQNTVRGSKRLIGRTFDDPQTQKEMKMVPYKIVRGPNGDAWVEMGGQQYSPSQIGAFVLTKMKETAEAFLGKTVSKAVITVPAYFNDAQRQATKDAGRIAGLEVMRIINEPTAAALSYGMNNKEGLIAVFDLGGGTFDVSILEISNGVFEVKATNGDTFLGGEDFDGALLDYLVSEFKKSDNIDLSKDKLALQRLREAAEKAKVELSSTMQTEINLPFITADATGAKHFNITLTRSKFESLVQSLIERTRIPCVNCLKDAGVSAKDIDEVLLVGGMTRVPKVQDIVSQIFNKTPSKGVNPDEAVAMGAAIQGGILRGDVKELLLLDVTPLSLGIETLGGIFTRLINRNTTIPTKKSQVFSTAADNQTQVGIKVLQGEREMATDNKLLGEFQLEGIPPAPRGMPQIEVTFDIDANGIVKVSAKDKSTGKEQEITIKSSGGLSESDIEKMVREAELHSQKDQERKSLIDLKNSADTTIYSIEKSVSEYKDKVPAEVTNEIQSAVSDLRAAMAEDDLEKIKQKLEAANKAVSKIGEHMQQGGGGGSGGGSSSSSGGDQTPEAEYQDAAKEAKM*

>LOC_Os02g53420 (mtHsp70-1)

MAASLLLRAARRRDLASPLGTLTANAQSAYSANICSQWGSFARAFSVKPTGNEVIGIDLGTTNSCVSVMEGKNPKVIENSEGTRTTPSVVAFNQKGERLVGTPAKRQAVTNPQNTFFGTKRLIGRRFEDPQTQKEMKMVPYKIVKAPNGDAWVETTDGKQYSPSQIGAFVLTKMKETAESYLGKTVSKAVITVPAYFNDAQRQATKDAGRIAGLDVQRIINEPTAAALSYGTNNKEGLIAVFDLGGGTFDVSILEISNGVFEVKATNGDTFLGGEDFDNTLLEFLVSEFKRSEAIDLAKDRLALQRLREAAEKAKIELSSTAQTEINLPFITADASGAKHLNITLTRSKFESLVNSLIERTREPCKNCLKDAGITTKEVDEVLLVGGMTRVPKVQEIVSEIFGKSPSKGVNPDEAVAMGAAIQGGILRGDVKELLLLDVTPLSLGIETLGGIFTRLINRNTTIPTKKSQVFSTAADNQTQVGIRVLQGEREMATDNKLLGEFDLVGIPPAPRGMPQIEVTFDIDANGIVTVSAKDKSTGKEQQITIRSSGGLSEAEIQKMVQEAELHSQKDQERKALIDIRNNADTTIYSVEKSLGEYRDKIPAEVATEIETAIADLRSVMTSDDIEKIKANIEAANKAVSKIGQHMSGGGGGAGGSETGGSQGGGEQAPEAEYEEVKK*

>LOC_Os03g02260 (mtHsp70-2)

MAASLLLRAVRRRDLASPLGTLTANVQSKCAANVCSRWAGFARTFSAKATGNEVIGIDLGTTNSCVSVMEGKNPKVIENSEGTRTTPSVVAFNQKGELLVGTPAKRQAVTNPQNTFFGTKRLIGRRFDDPQTQKEMKMVPYKIVKALNGDAWLETTDGKQYSPSQIGAFVLTKMKETAESYLGKSVSKAVITVPAYFNDAQRQATKDAGRIAGLDVQRIINEPTAAALSYGTNNKEGLIAVFDLGGGTFDVSILEISNGVFEVKATNGDTFLGGEDFDNTLLEFLVSEFKRTEGIDLSKDRLALQRLREAAEKAKIELSSTAQTEINLPFITADSSGAKHLNITLTRSKFESLVNSLIERTRDPCKSCLKDAGITTKDVDEVLLVGGMTRVPKVQEVVSEIFGKAPSKGVNPDEAVAMGAAIQGGILRGDVKDLLLLDVTPLSLGIETLGGIFTRLINRNTTVPTKKSQVFSTAADNQTQVGIKVLQGEREMAADNKLLGEFDLVGIPPAPRGMPQIEVTFDIDANGIVTVSAKDKATGKEQQITIRSSGGLSEAEIQKMVHEAELHSQKDQERKALIDIRNTADTTIYSIEKSLGEYRDKIPAEVASEIETAIADLRNEMASDDIEKIKSKIEAANKAVSKIGQHMSGGGSGGSQAGSQGGGDQAPEAEYEEVKK*

>LOC_Os12g05760 (Hsp110-8)

MARHILAAAAATALVLLCCFHLPATAMMADTIRYGPPRVPHRLGTVIAVDLGNTNSCVAGYGDEADAPPLFRLCIPTSVAFTGDGDALVGEAAKNHPAAISGFKRLLGTRFGSPEVRRAAEHLPYKIVDWCTMAHIEVNAGAGGAARSVYASDVASMVIAELKARAEARLAGGGKKVHNAVVTVPYYFSDGPREAAMNAARMAGLTTVRIIDEPTAAAVSHGLHHGRLRDGGNVLVLHVGGGTSAATVLTYDNAVFEAVASRHDAHLGGDDFDARIAGRFSQLIKRDHGGGVDDIAPAKLKSQCELAKRTLSSHDVAQVNLHATNLANGAFSFSGSLTRAQFEELNHDLFEKVISLVDAAMAEARRAVAGFDVIDEVVLVGGSTKIPRIRELIKNYFAGKEATVKATASIGGGAVVVVEPEEAVVHGGGLLSHPMEDGYHCMGAGGRRQIGSPMDRCYHEF*

>LOC_Os05g51360 (Hsp110-5)

MARRRLVLLLLIIAAAGAAAFGIRRDAPKAYCFYGIEWTGVPYLPGTAAAIYIGNTNSCIAGYHSPPDTATSYRFCIPSWVAFTANATTLCGQPAVDHSAAISGFKRLIGLQPGDPHAKRVAQIAPYKLGEKIGRCSIQVQLDDGAKCRVEDFLPEDVAGILIAHLKSTAEAHLGHRIDNAVVTVPGHFNGNQRQEVSSGSTEYGGFRYVSVVDEQVAAAAAHGLHEDRGDGKVILVFHLGGRTAHATKFVIRDGTPSLIALRHDPFLGGDDFTARVVDHMADLIKDKHGGRDVRADAAALRRLTAECERAKKALSYQQETVVTMRLDDDDDLFSEPLTRSKLEELIGDLVGRAVDLVESCDASGGDVVGVDEILLVGGSTRIPMVRDLVKDYFHGKEASNEKGVEPDEAVIRGALLLSHPHQARYLDPCYDYWHSR*

>LOC_Os06g10990 (Hsp110-6)

MGRRRLLLSDLLLLLLIAIAVAGAAAAIAIPRAAPAFGVETGWPPEHCLRCFAPPDAPFVLGAAAAIHLGNTNSCIAGYDDDDAPLGAKRSYYQFCIPSWVALAHDNGTVISGEAAMNRAALSPSTAVSAFMRLLHRRVEDDVVKREIELVPYKFTKMLGWVSVQLDTDAEFSVDHLAGILISHLKHTAEAHLGRHINNAVITLPSRLSYSADGRQVLSSAAKEYSGFRAVKVVDEHIAAAAAYGHHTKQGDRKAILVFHLGGRTSHATIFKFVDGTARLIATRAHHFLGGDDFTARIVDHMVEHIKEQHGRDVRQEEKAMVRLRVACEHAKKALSEQQETLVQMDSLLDDGAVFSATLTRAKFEELNHDLLDRAMALVKEVVVTTGGVEVVDEVLVVGGSARIPKVRQLVKDYFNGNGNGTHPNSRGCKGPVDVEPEDAVLHGAALLSRPLPVAEGTAAARSIGSVGGI*

>LOC_Os03g11910 (Hsp110-3)

MTEQLYTVASDSETTGEDKSQMSFPDVAIGIDIGTSNCSVAVWTGHQVELLKNTRNQKGMRSYVMFKDDSLSAGVTGGATREHGHEERDILSGSAIFNMKRLIGRMDTDEVVQASKSLPFLVQTLGIGVRPFIAALVNNMWRSTTPEEVLAIFLLELKALVEMHLKHPVRNAVLTIPVAFSRFQQTRIERACAMAGLHVLRLMPEPTAIALLYAQQQQQLLHDNMGSGIEKIALIFNMGAGYCDAAVAATAGGVSQIRALSGSTVGGEDILQNVMRHLMPDFDSLYAGQTMDRIKSIGLLRMATQDAIHKLATQEHVEINVDLGAGHKVSKILDRGEFEKVNQSIFEKCEGIIKQCLADAKLTPEDINDVILVGGCSRIPKIRSVVLGLCKKDDSYSGIDPLEAAVSGAALEGAIASGVSDPSGSLDLLTIQATPMNLGIRADGGNFAAIIPRNTAVPARRDMLFTTTHDNQTEALIAVYEGEGNQAEDNHLLGYFKITGIPAAAKGTVEINVCMDIDAGNVLRVFAGVVKPQGQAVPPFIEVRMPTLDDGHGWCGQALAKMYGSTLDLATIPKKLHP*

>LOC_Os02g48110 (Hsp110-2)

MAPPRTSGLLLLLAAVVAAAAVVLVPPAEAAVASIDLGSEWLKVAAVHLAPGRVPIAVAINEMSKRKSPALAALADGNRLAGEEASGITARHPSKVFARARDLLAKPFPYVRSVAEALFLPYDLVPDARGAAAVRADDGQVYTVEEIVAMVLHYAAGLADAHVGAPVRDAVVAVPPYFGQAERRALTQAAQLAGVNVLALINEHAGAALQYGIDKDFSNESRHVIFYDMGAGSTYAALVYYSAYKAKEFGKTVSVNQFQVKDVRWDSKLGGLEMEMRLVNYFADQFNKQLGNGVDIRQSPKAMAKLKKQVKRTKEILSANTAAPISVESLYNDLDFRSTITREKFEELCEELWEQALTPVKEVLAHSGMKIDDIYAVELIGGATRVPKLQAKLQEFLGRSDLDKHLDADEAIVLGASLHAANLSDGIKLNRKLGMIDGSTYGFVFEINGPDYVKDESTDQLLVPRMKKLGIKMFRSIRHTKDFDVSISYEKASELPPGVTSHKFVEYSVSGLTDASEKYSSRNLSAPIKANLHFSLSRSGIISLDRAEAVIEITEWVEVPKKNLTLESNSTSQTLSSEGGAANDTSDSKENVSSDGDANKSSAPIDESNAQDIVTEKVLKKRTFRVPLKVVEKMAGAGSILSKELYSEAKTRLEALDKKDAERRRTAELKNNLESYIYSMKEKLEENTEILTVSTEQERESFAEKLNEVQDWLYMDGEDAQANEFKERLDQLKAIGDPILFRLSELKARPTACENARLYLAELQKIVKNWDSNKPWLPKKRVDEVVSEAEKVKTWLEEKEAIQKSTPVYSPPAFTSEEVYEKVLDLQDKVSSVNRIPKPKPKIEKKPPKEEESANKEKTDSSESESKEAESTETSSESAAPEESQSEPQKTDDLEPEAHDEL*

>LOC_Os06g46600 (Hsp110-7)

MSVVGFDVGNDTLVAAAARQRGIDVLLNAESNRESPAAVAFSHNARLLGPHAAGAASSHAPFSSIKRLLLLAGRPTLLPRRGGDLSRLPFPVEASSADGGGGVLVHVDHIGRRIALSPTQLLAMLLGYLRQLAEADLEAPVSDCVISVPCYFTQAQRQAYLDAAAVAGLRPLRLMHDLAATALGYGLYRSDLGGPGGPTYVAFVDVGHCDTQVAVVAFDVSGMKVLSHRFDADLGGRDFDEVLFEHFAEEFRDKYKIDVTGNVKASMRLRAACEKAKKVLSANAEAVVNIECLMEEKDVRGMIRREEFEKLCAGLLERVVEPCKKAMEGSRIGFDRLHSVELVGSGSRVPAIARILAGFFRREPSRTLNASECVARGCALQCAMLSPTFRVREYEVQDAIPSSIGFCTSEGPISTLPSNALFQRGHPLPSVKVVTLHKNSKFKLDAFYVDENELPPGTSTKIGAFQIGPFQAHTEKSKVKVRIRLNLHGLVSVESAALIDDDQSDAHSADSMEVDSNGEMGQQVDKSRSERLIQLPIVQSIYGAMSNQELLEAQEQESQLAYQDKLMERTKERKNALESYVYDTRNKLSERYRSFATDSEREEISLSLQQTEDWLYEEGDDETEAVYNSKLEELKRLVDPIENRCKDEEVRGQATRDLLKFILDHKTAAKSLPTPEQEAVDSECTKAEQWLRERSQLQESLPKNVDPALWSHEIKKKEHELDMFYRNIVRYKGSPARADSSGGSDHMHTTDRD*

>LOC_Os01g08560 (Hsp110-1)

MSVVGFDLGNESCIVAVARQRGIDVVLNEESKRETPAIVCFGDKQRFIGTAGAASSTMNPKNSVSQIKRLLGRKYSDPELQRDIAAFPFRVSEGPDGFPLVHARYLGEERVFTPTQLMAMVLSNLKGIAESNLNTAVVDCCIGIPVYFTDLQRRAVLDAATIAGLCPLRLFHETTATALAYGIYKTDLPENDQLNVAFVDVGHASMQVCIAGYKKGQLKILSHAYDRSLGGRDFDEVLFKHFAAKFKDEYKIDVYQNARACIRLRVACEKLKKVLSANPESPMHIECLMDEKDVRGFIKREEFEKISASILERVKGPLEKALAEAGLTTENVHFVEVVGSGSRVPAIIKILTDFFGKEPRRTMNASECVARGCALECAILSPTFKVREFQVNDGFPFSIAMSWKPDSQNGDNQQTVVFPKGNPLPSVKALTFYRSNTFQVDVTYVDTGDLQISPKISTYTVGPFNPGKGDKAKLKVKVRLNIHGVVTVESATMLEEEEVEVPVAATTEPPKDSAKMETDDAPNEAASGTDVNMQEAKAPADAAADGAENGAPNSEEKSVPMETDAKVEPSKKKVKKTNVPVAELVYGALGTTELQKAVEKEYEMALQDRVMEETKDKKNAVESYVYDMRNKLYDKYNDFVTAEDKEAFIAKLQEVEDWLYEDGEDETKGVYVAKLEELKKVGGPIEARYKEWMDRGPSIDQLAYCINSFRDAALSKDPKFDHIEMEEKQKVINQCSEAEVWLREKIQQQDALPKHANPVLLSSDLKKKAETVDRFCKPIMMKPKPAPKPQTPPQTPPTETPAGGAQTPEQQPQGAEAAGEASEGGASESTGEQMETDKPEGTEAA*

>LOC_Os05g08840 (Hsp110-4)

MSVVGFDVGNESGIVAVARQRGIDVVLNEESKRETPAVVCFGDKQRFIGTAGAASSTMNPRNSVSQIKRLLGRAFADPELQRDLASFPFRVSEGPDGFPLVHARYLGEDRAFTPTQLLAMVLSNLKGIAEGNLNAAVFDCCIGIPAYFTDLQRRAVADAAAIAGLRPLRLFHETTATALAYGIYKTDLPEKEWLNVAFIDVGHASMQVSIVGYKKGQLNMLSHAYDRSLGGRDFDEVLFKHFAEKFKDEYKIDVYQNARACVRLRVACEKLKKMLSANPEAPLNIECLMDEKDVRGFIKREEFEQISSPVLQRVKAPLEKALAEAGLTTENVHFVEVVGSGSRVPAIIRIITEFFGKEPRRTMNASECVARGCALQCAVLSPTFKVREFEVNDGFPFSIALSCKPDSENTESEQTIVFPKGSPVPSAKTVTFYRSNTFAVDVVSVDADDLQMAKKISSYTIGPFQSSKPEKAKVNVKACLNIHGIVSIESAMMLEEEVDVPVATTNETLKDDTKMDTDDALGDPASGTDENMQESKCSADATHGAAENGKPDSEEISAPMDTDAKVEPLIKNVKKIDVPVSGLVYGALGSEELVKASENEYEMALQDRVMEETKEKKNAVEAYVYDMRNKLYDKYNDFVMSEYKEGFIAKLQEVEDWLYEDGEDETKGVYIAKLEELKKVGDPIEIRYKEWAERSSSINQLVHCINGFKEVALSNSQAFDHIDMSEKQKVLDECSEAEIWLIEKQQQQDALPKHADPVLLISDMKKKAEALDRSCRPIMSKPKPAPKPQTPPPPTPPTESPTTPEPQTPEQQQQSNGAGEAEEPTSEGGAQDQEPTAEQMDTDKPDGWAEPSA*

>AT1G11660

MSVVGFDVGNENCVIAVAKQRGIDVLLNDESNRENPAMVSFGEKQRFMGAAAAASATMHPKSTISQLKRLIGRKFREPDVQNDLRLFPFETSEDSDGGIQIRLRYMGEIQSFSPVQILGMLLSHLKQIAEKSLKTPVSDCVIGIPSYFTNSQRLAYLDAAAIAGLRPLRLMHDSTATALGYGIYKTDLVANSSPTYIVFIDIGHCDTQVCVASFESGSMRVRSHAFDRNLGGRDFDEVLFNHFALEFKEKYNIDVYTNTKACVRLRASCEKVKKVLSANAEAQLNIECLMEEKDVRSFIKREEFEQLSAGLLERLIVPCQKALADSGLSLDQIHSVELVGSGSRIPAISKMLSSLFKRELGRTVNASECVARGCALQCAMLSPVFRVRDYEVQDSYPFAIGFSSDKGPINTPSNELLFPKGQIFPSVKVLTLHRENTFQLEAFYANHNELSPDIPTQISSFMIGPFHISHGEAARVKVRVQLNLHGIVTIDSATLIEYHKENITSEEMISEENHQSSAMKDGSLDPSSGSIGNEPKAIKRMEIPVVANVSGALTKDELSEAKQRENSLVEQDLKMESTKDKKNALESFVYEMRDKMLNTYRNTATESERECIARNLQETEEWLYEDGDDESENAYIEKLNDVKKLIDPIENRFKDGEERVQASKDLLKTIADNRMAAESLPPPRKNAVLDECHKAERWLHEKTTEQESLPKDANPELQSAEIRRKADALNATCKYIGKSNSPPAKPEHNGSYGSRKSDDMELD

>At1g09080 (BIP3)

MIFIKENTAKMTRNKAIACLVFLTVLDFLMNIGAALMSSLAIEGEEQKLGTVIGIDLGTTYSCVGVYHNKHVEIIANDQGNRITPSWVAFTDTERLIGEAAKNQAAKNPERTIFDPKRLIGRKFDDPDVQRDIKFLPYKVVNKDGKPYIQVKVKGEEKLFSPEEISAMILTKMKETAEAFLGKKIKDAVITVPAYFNDAQRQATKDAGAIAGLNVVRIINEPTGAAIAYGLDKKGGESNILVYDLGGGTFDVSILTIDNGVFEVLSTSGDTHLGGEDFDHRVMDYFIKLVKKKYNKDISKDHKALGKLRRECELAKRSLSNQHQVRVEIESLFDGVDFSEPLTRARFEELNMDLFKKTMEPVKKALKDAGLKKSDIDEIVLVGGSTRIPKVQQMLKDFFDGKEPSKGTNPDEAVAYGAAVQGGVLSGEGGEETQNILLLDVAPLSLGIETVGGVMTNIIPRNTVIPTKKSQVFTTYQDQQTTVTINVYEGERSMTKDNRELGKFDLTGILPAPRGVPQIEVTFEVDANGILQVKAEDKVAKTSQSITITNDKGRLTEEEIEEMIREAEEFAEEDKIMKEKIDARNKLETYVYNMKSTVADKEKLAKKISDEDKEKMEGVLKEALEWLEENVNAEKEDYDEKLKEVELVCDPVIKSVYEKTEGENEDDDGDDHDEL

>At1g16030 (HSP70B)

MATKSEKAIGIDLGTTYSCVGVWMNDRVEIIPNDQGNRTTPSYVAFTDTERLIGDAAKNQVALNPQNTVFDAKRLIGRKFSDPSVQSDILHWPFKVVSGPGEKPMIVVSYKNEEKQFSPEEISSMVLVKMKEVAEAFLGRTVKNAVVTVPAYFNDSQRQATKDAGAISGLNVLRIINEPTAAAIAYGLDKKGTKAGEKNVLIFDLGGGTFDVSLLTIEEGVFEVKATAGDTHLGGEDFDNRLVNHFVAEFRRKHKKDIAGNARALRRLRTACERAKRTLSSTAQTTIEIDSLHEGIDFYATISRARFEEMNMDLFRKCMDPVEKVLKDAKLDKSSVHDVVLVGGSTRIPKIQQLLQDFFNGKELCKSINPDEAVAYGAAVQAAILTGEGSEKVQDLLLLDVAPLSLGLETAGGVMTVLIPRNTTVPCKKEQVFSTYADNQPGVLIQVYEGERARTRDNNLLGTFELKGIPPAPRGVPQINVCFDIDANGILNVSAEDKTAGVKNQITITNDKGRLSKEEIEKMVQDAEKYKAEDEQVKKKVEAKNSLENYAYNMRNTIKDEKLAQKLTQEDKQKIEKAIDETIEWIEGNQLAEVDEFEYKLKELEGICNPIISKMYQGGAAAGGMPTDGDFSSSGAAGGPKIEEVD

>At1g56410 (HSP70T-1)

MAGKGEGPAIGIDLGTTYSCVGVWQHDRVEIIANDQGNRTTPSYVAFTDSERLIGDAAKNQVAMNPVNTVFDAKRLIGRRFSDASVQSDMKFWPFKVTPGQADKPMIFVNYKGEEKQFAAEEISSMVLIKMREIAEAYLGSSIKNAVVTVPAYFNDSQRQATKDAGVIAGLNVLRIINEPTAAAIAYGLDKKATSVGIKNVLIFDLGGGTFDVSLLTIEEGIFEVKATAGDTHLGGEDFDNRMVNHFVQEFKRKNKKDISGDARALRRLRTACERAKRTLSSTAQTTVEVDSLFEGIDFYSPITRAKFEEMNMDLFRKCMEPVMKCLRDSKMDKSMVHDVVLVGGSTRIPKVQQLLQDFFNGKELCKSINPDEAVAYGAAVQAAILSGEGNEKVQDLLLLDVTPLSLGIETIGGVMTTLIQRNTTIPAKKEQEFTTTVDNQPDVLIQVYEGERARTIDNNILGQFVLSGIPPAPRGIPQFTVCFDIDSNGILNVSAEDKATGKKNKITITNDKGRLSKDDIEKMVQEAEKYKSEDEEHKKKVEAKNGLENYAYNVGNTLRDMGEKLPAADKKKFEDSIEEVIQWLDDNQLAEADEFEHKMKELESVWSTIITKMYQG

>At1g79920 (HSP70-15)

MSVVGFDFGNENCLVAVARQRGIDVVLNDESNRETPAIVCFGDKQRFIGTAGAASTMMNPKNSISQIKRLIGRQFSDPELQRDIKSLPFSVTEGPDGYPLIHANYLGEIRAFTPTQVMGMMLSNLKGIAEKNLNTAVVDCCIGIPVYFTDLQRRAVLDAATIAGLHPLHLIHETTATALAYGIYKTDLPENDQLNVAFIDIGHASMQVCIAGFKKGQLKILSHAFDRSLGGRDFDEVLFNHFAAKFKDEYKIDVSQNAKASLRLRATCEKLKKVLSANPMAPLNIECLMAEKDVRGVIKREEFEEISIPILERVKRPLEKALSDAGLTVEDVHMVEVVGSGSRVPAMIKILTEFFGKEPRRTMNASECVSRGCALQCAILSPTFKVREFQVHESFPFSISLAWKGAATDAQNGGTENQQSTIVFPKGNPIPSVKALTFYRSGTFSIDVQYSDVNDLQAPPKISTYTIGPFQSSKGERAKLKVKVRLNLHGIVSVESATLLEEEEVEVSVTKDQSEETAKMDTDKASAEAAPASGDSDVNMQDAKDTSDATGTDNGVPESAEKPVQMETDSKAEAPKKKVKKTNVPLSELVYGALKTVEVEKAVEKEFEMALQDRVMEETKDRKNAVESYVYDMRNKLSDKYQEYITDSEREAFLANLQEVEDWLYEDGEDETKGVYVAKLEELKKVGDPVEVRYKESLERGSVIDQLGYCINSYREAAVSNDPKFDHIELAEKQKVLNECVEAEAWLREKQQQQDTLPKYATPALLSADVKSKAEALDKFCRPIMTKPKPAAKAEAPQAKGGEQADEGKSEPEQPASAEAMETENPAEGST

>At1g79930 (HSP70-14)

MSVVGFDFGNENCLVAVARQRGIDVVLNDESNRETPAIVCFGDKQRFIGTAGAASTMMNPKNSISQIKRLIGRQFSDPELQRDIKSLPFSVTEGPDGYPLIHANYLGEKRAFTPTQVMGMMLSNLKGIAEKNLNTAVVDCCIGIPVYFTDLQRRAVLDAATIAGLHPLRLIHETTATALAYGIYKTDLPESDQLNVAFIDIGHASMQVCIAGFKKGQLKILSHAFDRSLGGRDFDEVLFNHFAAKFKDEYKIDVSQNAKASLRLRATCEKLKKVLSANPLAPLNIECLMDEKDVRGVIKREEFEEISIPILERVKRPLEKALSDAGLTVEDVHMVEVIGSGSRVPAMIKILTEFFGKEPRRTMNASECVSRGCALQCAILSPTFKVREFQVHESFPFSISLAWKGAASEAQNGGAENQQSTIVFPKGNPIPSVKALTFYRSGTFSVDVQYSDVNDLQAPPKISTYTIGPFQSSKGERAKLKVKVRLNLHGIVSVESATLLEEEEVEVPVTKEHSEETTKMDSDKASAEAAPASGDCDVNMQDAKDTSDATGTDNGVPESAEKPVQMETDSKAEAPKKKVKKTNVPLSELVYGALKTVEVEKAVEKEFEMALQDRVMEETKDRKNAVESYVYDMRNKLSDKYQEYITDSEREAFLANLQEVEDWLYEDGEDETKGVYVAKLEELKKVGDPVEVRYKESLERGSVIDQLGYCINSYREAAMSTDPKFDHIELAEKQKVLNECVEAEAWLRGKQQQQDTLPKYATPALLSADVKSKAEALDKFCRPIMTKPKPVAKAEAPQAKGGEQADEGKSEPEQPASAEPMETENPAEGST

>At2g32120 (HSP70T-2)

MAEAAYTVASDSENTGEEKSSSSPSLPEIALGIDIGTSQCSIAVWNGSQVHILRNTRNQKLIKSFVTFKDEVPAGGVSNQLAHEQEMLTGAAIFNMKRLVGRVDTDPVVHASKNLPFLVQTLDIGVRPFIAALVNNAWRSTTPEEVLAIFLVELRLMAEAQLKRPVRNVVLTVPVSFSRFQLTRFERACAMAGLHVLRLMPEPTAIALLYAQQQQMTTHDNMGSGSERLAVIFNMGAGYCDVAVTATAGGVSQIKALAGSPIGGEDILQNTIRHIAPPNEEASGLLRVAAQDAIHRLTDQENVQIEVDLGNGNKISKVLDRLEFEEVNQKVFEECERLVVQCLRDARVNGGDIDDLIMVGGCSYIPKVRTIIKNVCKKDEIYKGVNPLEAAVRGAALEGAVTSGIHDPFGSLDLLTIQATPLAVGVRANGNKFIPVIPRNTMVPARKDLFFTTVQDNQKEALIIIYEGEGETVEENHLLGYFKLVGIPPAPKGVPEINVCMDIDASNALRVFAAVLMPGSSSPVVPVIEVRMPTVDDGHGWCAQALNVKYGATLDLITLQRKM

>At3g09440 (HSP70-3)

MAGKGEGPAIGIDLGTTYSCVGVWQHDRVEIIANDQGNRTTPSYVAFTDSERLIGDAAKNQVAMNPINTVFDAKRLIGRRFTDSSVQSDIKLWPFTLKSGPAEKPMIVVNYKGEDKEFSAEEISSMILIKMREIAEAYLGTTIKNAVVTVPAYFNDSQRQATKDAGVIAGLNVMRIINEPTAAAIAYGLDKKATSVGEKNVLIFDLGGGTFDVSLLTIEEGIFEVKATAGDTHLGGEDFDNRMVNHFVQEFKRKNKKDISGNPRALRRLRTACERAKRTLSSTAQTTIEIDSLFDGIDFYAPITRARFEELNIDLFRKCMEPVEKCLRDAKMDKNSIDDVVLVGGSTRIPKVQQLLVDFFNGKELCKSINPDEAVAYGAAVQAAILSGEGNEKVQDLLLLDVTPLSLGLETAGGVMTVLIQRNTTIPTKKEQVFSTYSDNQPGVLIQVYEGERARTKDNNLLGKFELSGIPPAPRGVPQITVCFDIDANGILNVSAEDKTTGQKNKITITNDKGRLSKDEIEKMVQEAEKYKSEDEEHKKKVDAKNALENYAYNMRNTIRDEKIGEKLAGDDKKKIEDSIEAAIEWLEANQLAECDEFEDKMKELESICNPIIAKMYQGGEAGGPAAGGMDEDVPPSAGGAGPKIEEVD

>At3g12580 (AtHSP70)

MAGKGEGPAIGIDLGTTYSCVGVWQHDRVEIIANDQGNRTTPSYVAFTDSERLIGDAAKNQVAMNPTNTVFDAKRLIGRRYSDPSVQADKSHWPFKVVSGPGEKPMIVVNHKGEEKQFSAEEISSMVLIKMREIAEAFLGSPVKNAVVTVPAYFNDSQRQATKDAGVISGLNVMRIINEPTAAAIAYGLDKKASSVGEKNVLIFDLGGGTFDVSLLTIEEGIFEVKATAGDTHLGGEDFDNRMVNHFVQEFKRKNKKDITGNPRALRRLRTACERAKRTLSSTAQTTIEIDSLFEGIDFYTTITRARFEELNMDLFRKCMEPVEKCLRDAKMDKSSVHDVVLVGGSTRIPKVQQLLQDFFNGKELCKSINPDEAVAYGAAVQAAILSGEGNEKVQDLLLLDVTPLSLGLETAGGVMTVLIPRNTTIPTKKEQIFSTYSDNQPGVLIQVYEGERARTKDNNLLGKFELSGIPPAPRGVPQITVCFDIDANGILNVSAEDKTTGQKNKITITNDKGRLSKEEIEKMVQEAEKYKAEDEEHKKKVDAKNALENYAYNMRNTIKDEKIASKLDAADKKKIEDAIDQAIEWLDGNQLAEADEFEDKMKELESLCNPIIARMYQGAGPDMGGAGGMDDDTPAGGSGGAGPKIEEVD

>At4g16660

MGKIFSWLVVLLSLISLVPVPSESAVLSVDLGSEWVKVAVVNLKRGQSPISVAINEMSKRKSPALVAFQSGDRLLGEEAAGITARYPNKVYSQLRDMVGKPFKHVKDFIDSVYLPFDIVEDSRGAVGIKIDDGSTVYSVEELLAMILGYASNLAEFHAKIPVKDMVVSVPPYFGQAERRGLIQASQLAGVNVLSLVNEHSGAALQYGIDKDFANGSRHVIFYDMGSSSTYAALVYYSAYSEKEYGKTVSVNQFQVKDVRWDLGLGGQSMEMRLVEHFADEFNKQLGNGVDVRKFPKAMAKLKKQVKRTKEILSANTAAPISVESLHDDRDFRSTITREKFEELCKDLWERSLTPLKDVLKHSGLKIDDISAVELIGGATRVPKLQSTIQEFIGKQQLDKHLDADEAIVLGSALHAANLSDGIKLKRRLGIVDGSPYGFLVELEGPNVKKDESTKQQLVPRMKKLPSKMFRSFVLDKDFDVSLAYESEGILPPGTTSPVFAQYSVSGLADASEKYSSRNLSAPIKANLHFSLSRSGILSLDRGDAVIEITEWVDVPKKNVTIDSNTTTSTGNATDENSQENKEDLQTDAENSTASNTTAEEPAVASLGTEKKLKKRTFRIPLKVVEKTVGPGAPFSKESLAEAKIKLEALDKKDRERRRTAELKNNLESYIYATKEKLETPEFEKISTQEERKAFVEKLDEVQDWLYMDGEDANATEFEKRLDSLKAIGSPISFRSEELTARPVAIEYARKYLTELKEIIKEWETNKTWLPKEKIDEVSKEAEKVKSWLDKNVAEQEKTSLWSKPVFTSTEVYAKVFTLQDKVTKVNKIPKPKPKIEKVTKTENTTKEEEQSKSSDEAAKEEESHDEL

>At4g24280 (cpHSC70-1)

MASSAAQIHVLGGIGFASSSSSKRNLNGKGGTFMPRSAFFGTRTGPFSTPTSAFLRMGTRNGGGASRYAVGPVRVVNEKVVGIDLGTTNSAVAAMEGGKPTIVTNAEGQRTTPSVVAYTKSGDRLVGQIAKRQAVVNPENTFFSVKRFIGRKMNEVDEESKQVSYRVVRDENNNVKLECPAINKQFAAEEISAQVLRKLVDDASRFLNDKVTKAVITVPAYFNDSQRTATKDAGRIAGLEVLRIINEPTAASLAYGFDRKANETILVFDLGGGTFDVSVLEVGDGVFEVLSTSGDTHLGGDDFDKRVVDWLAAEFKKDEGIDLLKDKQALQRLTEAAEKAKIELSSLTQTNMSLPFITATADGPKHIETTLTRAKFEELCSDLLDRVRTPVENSLRDAKLSFKDIDEVILVGGSTRIPAVQELVRKVTGKEPNVTVNPDEVVALGAAVQAGVLAGDVSDIVLLDVTPLSIGLETLGGVMTKIIPRNTTLPTSKSEVFSTAADGQTSVEINVLQGEREFVRDNKSLGSFRLDGIPPAPRGVPQIEVKFDIDANGILSVSAVDKGTGKKQDITITGASTLPKDEVDQMVQEAERFAKDDKEKRDAIDTKNQADSVVYQTEKQLKELGEKIPGEVKEKVEAKLQELKDKIGSGSTQEIKDAMAALNQEVMQIGQSLYNQPGAGGPGAGPSPGGEGASSGDSSSSKGGDGDDVIDADFTDSQ

>At4g37910 (mtHSC70-1)

MASVALLRSFRRREVQMASVSAFKSVSANGKNSMFGKLGYLARPFCSRPVGNDVIGIDLGTTNSCVSVMEGKTARVIENAEGSRTTPSVVAMNQKGELLVGTPAKRQAVTNPTNTIFGSKRLIGRRFDDPQTQKEMKMVPYKIVKAPNGDAWVEANGQKFSPSQIGANVLTKMKETAEAYLGKSINKAVVTVPAYFNDAQRQATKDAGKIAGLDVQRIINEPTAAALSYGMNNKEGVIAVFDLGGGTFDVSILEISSGVFEVKATNGDTFLGGEDFDNTLLEYLVNEFKRSDNIDLTKDNLALQRLREAAEKAKIELSSTTQTEINLPFITADASGAKHLNITLTRSKFEGLVGKLIERTRSPCQNCLKDAGVTIKEVDEVLLVGGMTRVPKVQEIVSEIFGKSPCKGVNPDEAVAMGAAIQGGILRGDVKDLLLLDVVPLSLGIETLGAVFTKLIPRNTTIPTKKSQVFSTAADNQMQVGIKVLQGEREMAADNKVLGEFDLVGIPPAPRGMPQIEVTFDIDANGITTVSAKDKATGKEQNITIRSSGGLSDDEINRMVKEAELNAQKDQEKKQLIDLRNSADTTIYSVEKSLSEYREKIPAEIASEIETAVSDLRTAMAGEDVEDIKAKVEAANKAVSKIGEHMSKGSGSSGSDGSSGEGTSGTEQTPEAEFEEASGSRK

>At5g02490 (HSP70-2)

MAGKGEGPAIGIDLGTTYSCVGVWQHDRVEIIANDQGNRTTPSYVAFTDSERLIGDAAKNQVAMNPVNTVFDAKRLIGRRFSDASVQSDRQLWPFTIISGTAEKPMIVVEYKGEEKQFAAEEISSMVLIKMREIAEAFLGTTVKNAVVTVPAYFNDSQRQATKDAGVIAGLNVLRIINEPTAAAIAYGLDKKATSVGEKNVLIFDLGGGTFDVSLLTIEEGIFEVKATAGDTHLGGEDFDNRMVNHFVQEFKRKNKQDITGQPRALRRLRTACERAKRTLSSTAQTTIEIDSLYGGADFYSPITRARFEEMNMDLFRKCMEPVEKCLRDAKMDKSTVHEIVLVGGSTRIPKVQQLLQDFFNGKELCKSINPDEAVAYGAAVQAAILSGEGNEKVQDLLLLDVTPLSLGLETAGGVMTTLIQRNTTIPTKKEQVFSTYSDNQPGVLIQVFEGERARTKDNNLLGKFELSGIPPAPRGVPQITVCFDIDANGILNVSAEDKTTGKKNKITITNDKGRLSKEDIEKMVQEAEKYKSEDEEHKKKVEAKNALENYAYNMRNTIRDEKIGEKLPAADKKKVEDSIEEAIQWLDGNQLGEADEFEDKMKELESVCNPIIAKMYQGGAGGEAGGPGASGMDEDEAPPASGGAGPKIEEVD

>At5g02500 (HSP70-1)

MSGKGEGPAIGIDLGTTYSCVGVWQHDRVEIIANDQGNRTTPSYVAFTDSERLIGDAAKNQVAMNPVNTVFDAKRLIGRRFSDSSVQSDMKLWPFKIQAGPADKPMIYVEYKGEEKEFAAEEISSMVLIKMREIAEAYLGVTIKNAVVTVPAYFNDSQRQATKDAGVIAGLNVMRIINEPTAAAIAYGLDKKATSVGEKNVLIFDLGGGTFDVSLLTIEEGIFEVKATAGDTHLGGEDFDNRMVNHFVQEFKRKSKKDITGNPRALRRLRTSCERAKRTLSSTAQTTIEIDSLYEGIDFYSTITRARFEELNMDLFRKCMEPVEKCLRDAKMDKSTVHDVVLVGGSTRIPKVQQLLQDFFNGKELCKSINPDEAVAYGAAVQGAILSGEGNEKVQDLLLLDVTPLSLGLETAGGVMTTLIPRNTTIPTKKEQVFSTYSDNQPGVLIQVYEGERARTKDNNLLGKFELSGIPPAPRGVPQITVCFDIDANGILNVSAEDKTTGQKNKITITNDKGRLSKDEIEKMVQEAEKYKSEDEEHKKKVEAKNALENYAYNMRNTIQDEKIGEKLPAADKKKIEDSIEQAIQWLEGNQLAEADEFEDKMKELESICNPIIAKMYQGAGGEAGGPGASGMDDDAPPASGGAGPKIEEVD

>At5g09590 (mtHSC70-2)

MATAALLRSIRRREVVSSPFSAYRCLSSSGKASLNSSYLGQNFRSFSRAFSSKPAGNDVIGIDLGTTNSCVAVMEGKNPKVIENAEGARTTPSVVAFNTKGELLVGTPAKRQAVTNPTNTVSGTKRLIGRKFDDPQTQKEMKMVPYKIVRAPNGDAWVEANGQQYSPSQIGAFILTKMKETAEAYLGKSVTKAVVTVPAYFNDAQRQATKDAGRIAGLDVERIINEPTAAALSYGMTNKEGLIAVFDLGGGTFDVSVLEISNGVFEVKATNGDTFLGGEDFDNALLDFLVNEFKTTEGIDLAKDRLALQRLREAAEKAKIELSSTSQTEINLPFITADASGAKHFNITLTRSRFETLVNHLIERTRDPCKNCLKDAGISAKEVDEVLLVGGMTRVPKVQSIVAEIFGKSPSKGVNPDEAVAMGAALQGGILRGDVKELLLLDVTPLSLGIETLGGVFTRLITRNTTIPTKKSQVFSTAADNQTQVGIRVLQGEREMATDNKLLGEFDLVGIPPSPRGVPQIEVTFDIDANGIVTVSAKDKTTGKVQQITIRSSGGLSEDDIQKMVREAELHAQKDKERKELIDTKNTADTTIYSIEKSLGEYREKIPSEIAKEIEDAVADLRSASSGDDLNEIKAKIEAANKAVSKIGEHMSGGSGGGSAPGGGSEGGSDQAPEAEYEEVKK

>At5g28540 (BIP1)

MARSFGANSTVVLAIIFFGCLFALSSAIEEATKLGSVIGIDLGTTYSCVGVYKNGHVEIIANDQGNRITPSWVGFTDSERLIGEAAKNQAAVNPERTVFDVKRLIGRKFEDKEVQKDRKLVPYQIVNKDGKPYIQVKIKDGETKVFSPEEISAMILTKMKETAEAYLGKKIKDAVVTVPAYFNDAQRQATKDAGVIAGLNVARIINEPTAAAIAYGLDKKGGEKNILVFDLGGGTFDVSVLTIDNGVFEVLSTNGDTHLGGEDFDHRVMEYFIKLIKKKHQKDISKDNKALGKLRRECERAKRALSSQHQVRVEIESLFDGVDFSEPLTRARFEELNNDLFRKTMGPVKKAMDDAGLQKSQIDEIVLVGGSTRIPKVQQLLKDFFEGKEPNKGVNPDEAVAYGAAVQGGILSGEGGDETKDILLLDVAPLTLGIETVGGVMTKLIPRNTVIPTKKSQVFTTYQDQQTTVSIQVFEGERSLTKDCRLLGKFDLNGIPPAPRGTPQIEVTFEVDANGILNVKAEDKASGKSEKITITNEKGRLSQEEIDRMVKEAEEFAEEDKKVKEKIDARNALETYVYNMKNQVNDKDKLADKLEGDEKEKIEAATKEALEWLDENQNSEKEEYDEKLKEVEAVCNPIITAVYQRSGGAPGGAGGESSTEEEDESHDEL

>At5g42020 (BIP2)

MARSFGANSTVVLAIIFFGCLFAFSTAKEEATKLGSVIGIDLGTTYSCVGVYKNGHVEIIANDQGNRITPSWVGFTDSERLIGEAAKNQAAVNPERTVFDVKRLIGRKFEDKEVQKDRKLVPYQIVNKDGKPYIQVKIKDGETKVFSPEEISAMILTKMKETAEAYLGKKIKDAVVTVPAYFNDAQRQATKDAGVIAGLNVARIINEPTAAAIAYGLDKKGGEKNILVFDLGGGTFDVSVLTIDNGVFEVLSTNGDTHLGGEDFDHRIMEYFIKLIKKKHQKDISKDNKALGKLRRECERAKRALSSQHQVRVEIESLFDGVDLSEPLTRARFEELNNDLFRKTMGPVKKAMDDAGLQKSQIDEIVLVGGSTRIPKVQQLLKDFFEGKEPNKGVNPDEAVAYGAAVQGGILSGEGGDETKDILLLDVAPLTLGIETVGGVMTKLIPRNTVIPTKKSQVFTTYQDQQTTVSIQVFEGERSLTKDCRLLGKFDLTGVPPAPRGTPQIEVTFEVDANGILNVKAEDKASGKSEKITITNEKGRLSQEEIDRMVKEAEEFAEEDKKVKEKIDARNALETYVYNMKNQVSDKDKLADKLEGDEKEKIEAATKEALEWLDENQNSEKEEYDEKLKEVEAVCNPIITAVYQRSGGAPGAGGESSTEEEDESHDEL

>At5g49910 (cpHSC70-2)

MASSAAQIHILGGIGFPTSSSSSSTKNLDNKTNSIPRSVFFGNRTSPFTTPTSAFLRMGRRNNNASRYTVGPVRVVNEKVVGIDLGTTNSAVAAMEGGKPTIVTNAEGQRTTPSVVAYTKSKDRLVGQIAKRQAVVNPENTFFSVKRFIGRRMNEVAEESKQVSYRVIKDENGNVKLDCPAIGKQFAAEEISAQVLRKLVDDASRFLNDKVTKAVITVPAYFNDSQRTATKDAGRIAGLEVLRIINEPTAASLAYGFERKSNETILVFDLGGGTFDVSVLEVGDGVFEVLSTSGDTHLGGDDFDKRVVDWLASTFKKDEGIDLLKDKQALQRLTEAAEKAKIELSSLTQTNMSLPFITATADGPKHIETTLTRGKFEELCSDLLDRVRTPVENSLRDAKLSFKDIDEVILVGGSTRIPAVQDLVRKLTGKEPNVSVNPDEVVALGAAVQAGVLSGDVSDIVLLDVTPLSLGLETLGGVMTKIIPRNTTLPTSKSEVFSTAADGQTSVEINVLQGEREFVRDNKSIGSFRLDGIPPAPRGVPQIEVKFDIDANGILSVSASDKGTGKKQDITITGASTLPKDEVDTMVQEAERFAKEDKEKRDAIDTKNQADSVVYQTEKQLKELGEKIPGPVKEKVEAKLQELKEKIASGSTQEIKDTMAALNQEVMQIGQSLYNQPQPGGADSPPGGEASSSSDTSSSAKGGDNGGDVIDADFTDSN
