# Supplementary figures and images for: Genome-wide survey of heat shock factors and heat shock protein 70s and their regulatory network under abiotic stresses in Brachypodium distachyon
Source: PLoS One. 2017 Jul 6;12(7):e0180352. doi: 10.1371/journal.pone.0180352 (PMC5500289; doi:10.1371/journal.pone.0180352)

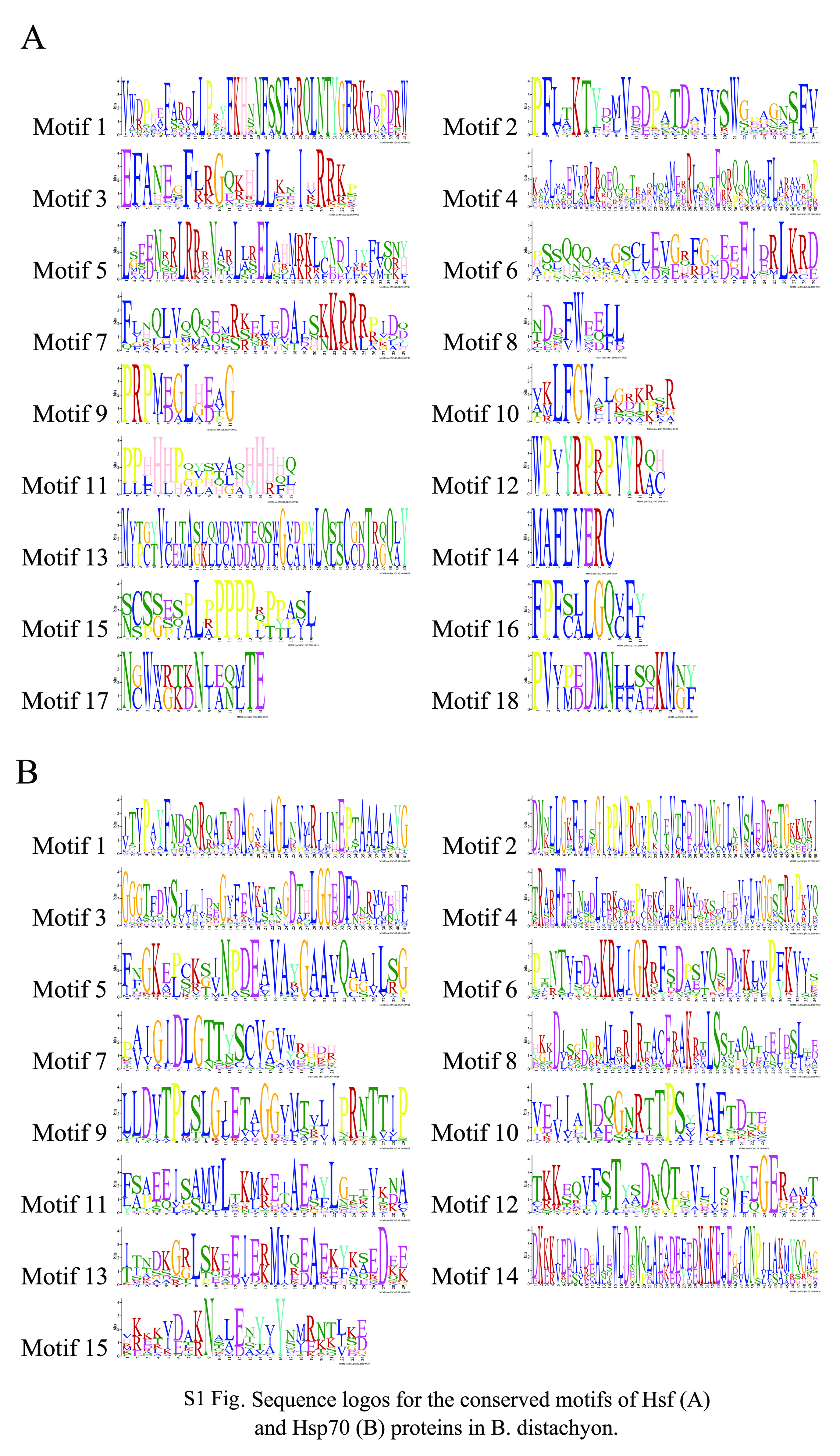

Supplement: S1 Fig — (TIF) [file pone.0180352.s006.tif]

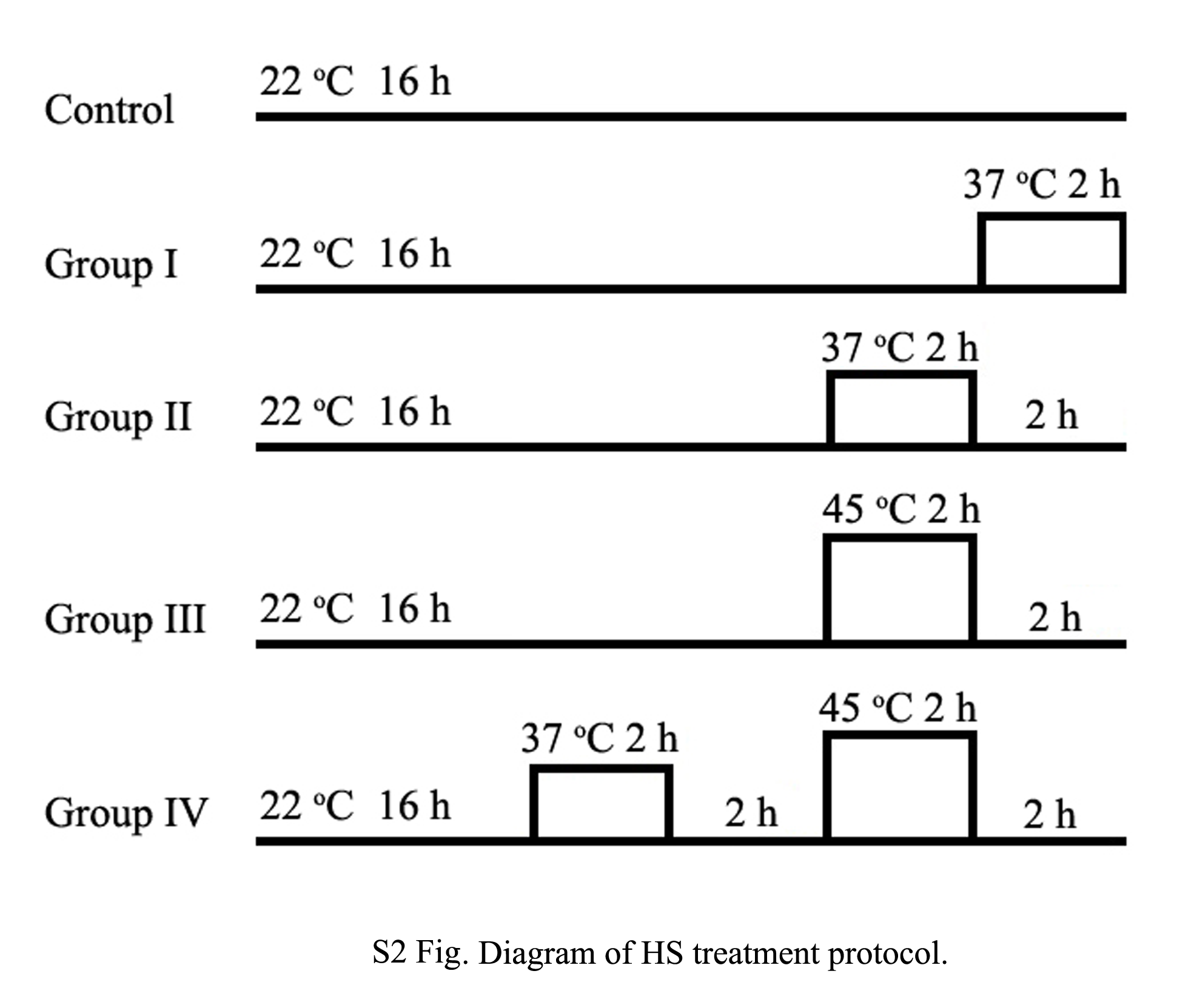

Supplement: S2 Fig — (TIF) [file pone.0180352.s007.tif]

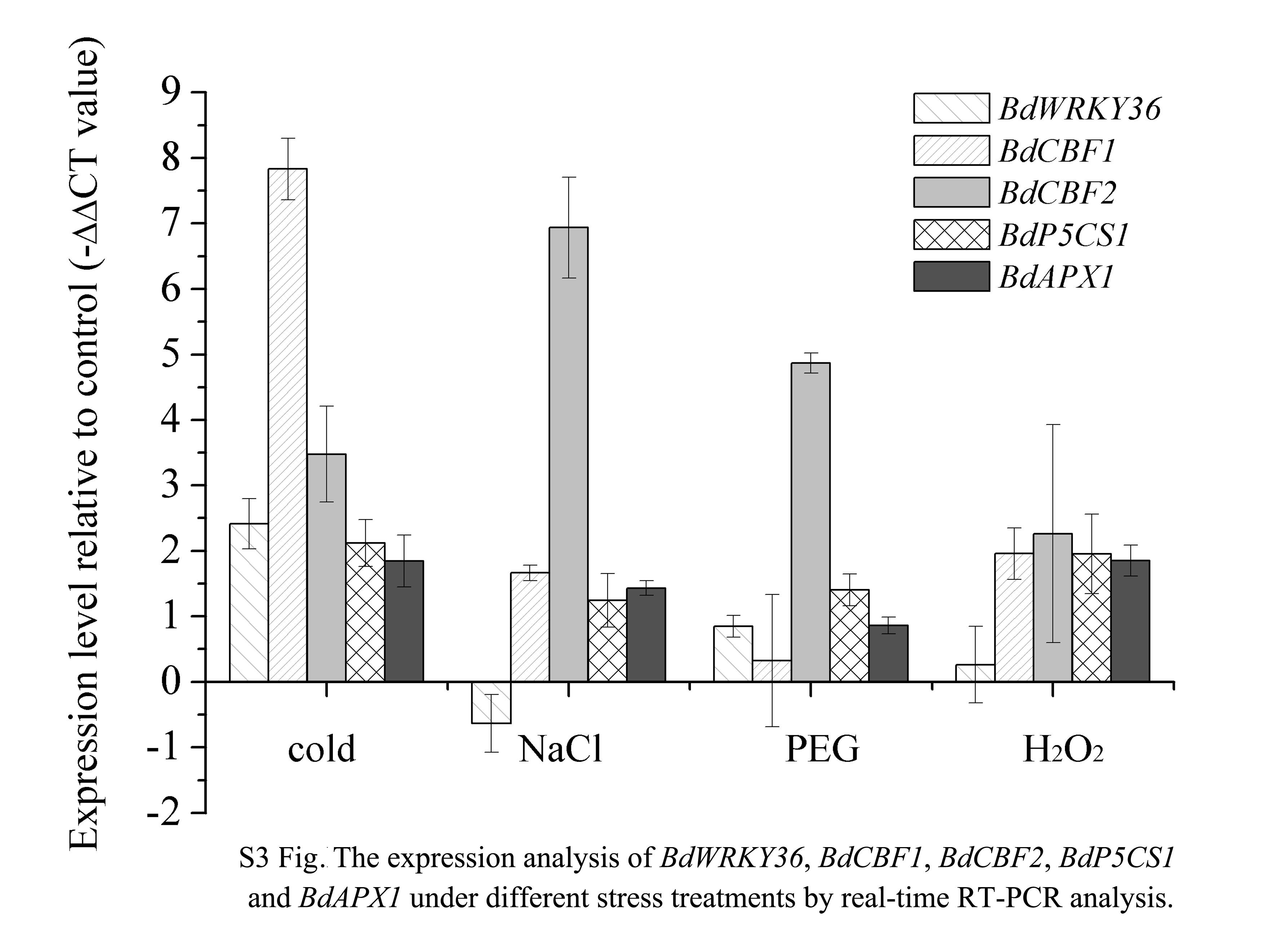

Supplement: S3 Fig — (TIF) [file pone.0180352.s008.tif]

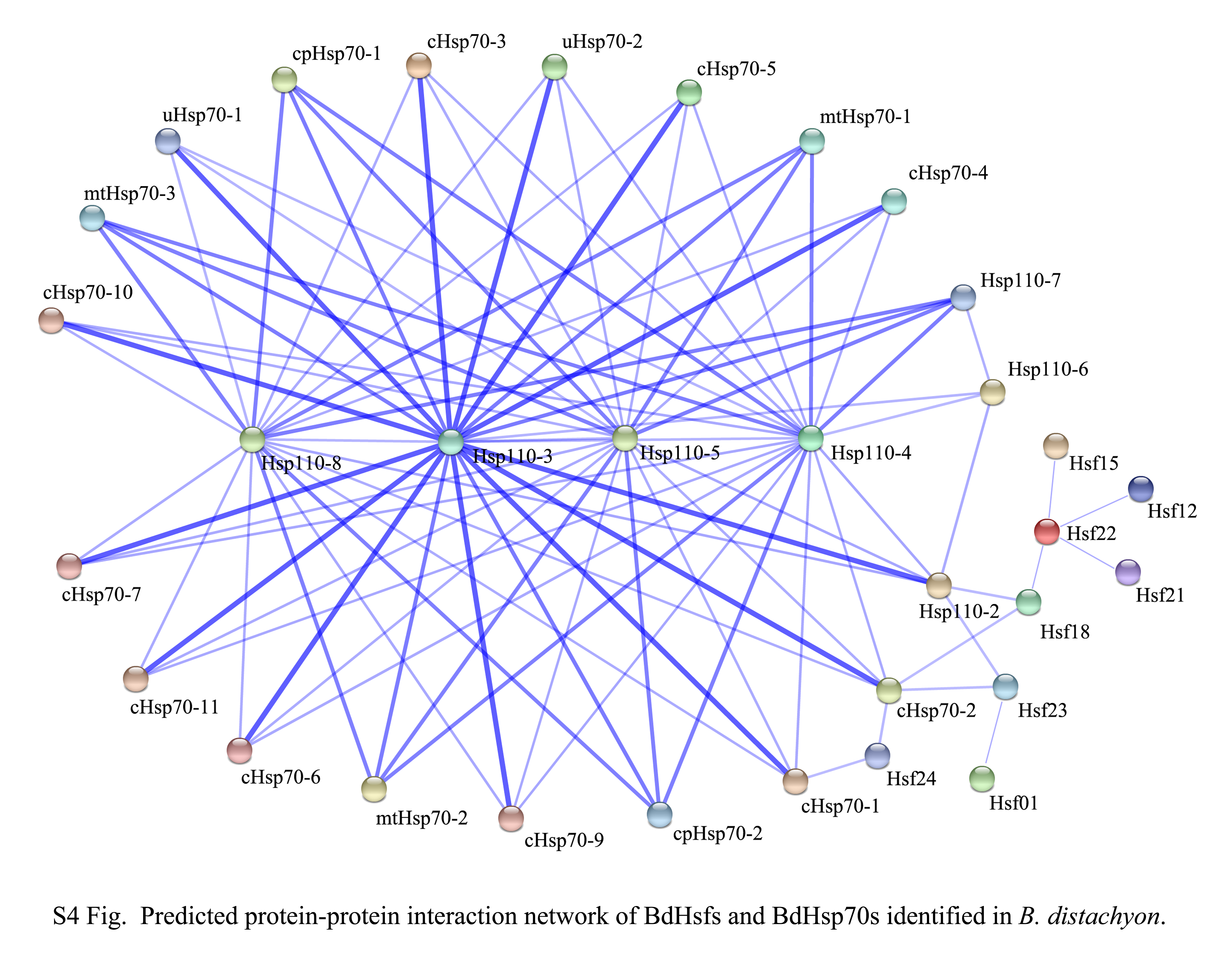

Supplement: S4 Fig — (TIF) [file pone.0180352.s009.tif]
